# Regulation of genes affecting body size and innate immunity by the DBL-1/BMP-like pathway in *Caenorhabditis elegans*

**DOI:** 10.1186/1471-213X-10-61

**Published:** 2010-06-07

**Authors:** Andrew F Roberts, Tina L Gumienny, Ryan J Gleason, Huang Wang, Richard W Padgett

**Affiliations:** 1 Waksman Institute, Department of Molecular Biology and Biochemistry, Cancer Institute of New Jersey, Rutgers University, Piscataway, NJ 08854-8020, USA; 2 Current Address: International Life Sciences Institute (ILSI) Research Foundation, Washington D.C. 20005, USA; 3 Current Address: Department of Molecular and Cellular Medicine, Texas A&M Health Science Center, College Station, TX 77843-1114, USA

## Abstract

**Background:**

Bone morphogenetic proteins (BMPs) are members of the conserved transforming growth factor β (TGFβ superfamily, and play many developmental and homeostatic roles. In *C. elegans*, a BMP-like pathway, the DBL-1 pathway, controls body size and is involved in innate immunity. How these functions are carried out, though, and what most of the downstream targets of this pathway are, remain unknown.

**Results:**

We performed a microarray analysis and compared expression profiles of animals lacking the SMA-6 DBL-1 receptor, which decreases pathway signaling, with animals that overexpress DBL-1 ligand, which increases pathway signaling. Consistent with a role for DBL-1 in control of body size, we find positive regulation by DBL-1 of genes involved in physical structure, protein synthesis and degradation, and metabolism. However, cell cycle genes were mostly absent from our results. We also identified genes in a *hedgehog*-related pathway, which may comprise a secondary signaling pathway downstream of DBL-1 that controls body size. In addition, DBL-1 signaling up-regulates pro-innate immunity genes. We identified a reporter for DBL-1 signaling, which is normally repressed but is up-regulated when DBL-1 signaling is reduced.

**Conclusions:**

Our results indicate that body size in *C. elegans *is controlled in part by regulation of metabolic processes as well as protein synthesis and degradation. This supports the growing body of evidence that suggests cell size is linked to metabolism. Furthermore, this study discovered a possible role for *hedgehog*-related pathways in transmitting the BMP-like signal from the hypodermis, where the core DBL-1 pathway components are required, to other tissues in the animal. We also identified the up-regulation of genes involved in innate immunity, clarifying the role of DBL-1 in innate immunity. One of the highly regulated genes is expressed at very low levels in wild-type animals, but is strongly up-regulated in Sma/Mab mutants, making it a useful reporter for DBL-1/BMP-like signaling in *C. elegans*.

## Background

Diverse cellular responses to TGFβ superfamily members are a hallmark of this family, with responses specified by cell type, time, or location within a TGFβ member gradient [1,2]. In *C. elegans*, a BMP-like family member, DBL-1 (Dpp and BMP-like), regulates not only body size, but also innate immunity and aspects of male tail development. Animals with reduced pathway signaling are small, while increased signaling results in long animals [3,4]. Animals with defective DBL-1 are also more susceptible to bacterial or fungal infection, and DBL-1 is highly up-regulated upon infection [5,6].

Body size and male tail development are separable by dose, as a weak *sma-6 *receptor mutant or a weak *sma-4*/SMAD mutant affects body size but not male tails [7]. Furthermore, TGFβ pathway regulators also differentiate between body size and male tail development. For instance, *sma-11/kin-29*, *bra-1*, and *lon-2*/glypican affect body size but not male tails. MAB-23/DOUBLESEX transcription factor, on the other hand, affects DBL-1 male tail development independently of body size [8]. The LIN-31 forkhead transcription factor may also play a role in DBL-1 mediated male tail development. *lin-31 *mutant males have crumpled spicules similar to *dbl-1 *mutant males, and forkhead transcription factors are known to be Smad co-factors [9,10].

How is specificity achieved? We performed a microarray experiment comparing populations of mRNAs from animals with increased or decreased DBL-1/BMP signaling. We discovered that transcriptional control of body size in *C. elegans *acts through the regulation of metabolism, protein synthesis/degradation, and structural genes, and not obviously by cell cycle genes. Furthermore, we have identified a subset of the hedgehog-related genes (warthogs) as targets of the DBL-1 pathway, and propose that they act as downstream transducers of DBL-1 signaling for body size determination. In addition, we now better understand the role that DBL-1 plays in innate immunity, as our results show that genes known to be involved in innate immunity, namely lysozymes, lipase, and lectins, are regulated by the DBL-1 signaling pathway. A large number of other intestinally expressed genes, a primary site of innate immunity, are also highly regulated by DBL-1, suggesting a broader role for DBL-1 in the intestinal innate immune response. Finally, we created a fluorescent biomarker for DBL-1 activity, and showed that the reporter accurately identified known DBL-1 signaling components.

## Results and Discussion

### Microarray hybridization and analyses of gene expression profiles

We compared gene expression in *C. elegans *strain BW1940 overexpressing *ctIs40*, an integrated transgene carrying wild-type *dbl-1*, and a strain lacking functional DBL-1 Type I receptor, LT186 *sma-6(wk7) *[4,7]. *sma-6(wk7)*, which encodes a stop codon at Y72 predicted to truncate the protein prematurely in its extracellular domain, has reduced *sma-6 *RNA expression levels [7]. BW1940 animals are longer than the wild type, and LT186 animals are smaller than normal. These strains have not been tested for response to an immune challenge, however *dbl-1(nk3) *animals are more susceptible to infection by pathogenic bacteria [5,11-13].

We performed our microarray analyses with the Affymetrix *C. elegans *whole genome GeneChip array, which represents over 22,000 unique transcripts (Affymetrix, CA, #900383). Five independent experiments were averaged and analyzed. About 2400 genes show a change in expression at the 95% confidence level (<12% of total transcripts in the array), with about 1800 transcripts showing up-regulation of transcription in response to BMP signaling (positive regulation) and about 800 showing a down-regulation of transcription (negative regulation). 276 genes are regulated within a 99.9% confidence interval, with 186 positively regulated and 90 negatively regulated genes (1.2% of total unique genes in the array)(Additional file 1). None of the genes in the 99.9% confidence interval show a change in expression less than 1.5-fold (Additional file 2).

From our microarray results, we find that BW1940 *ctIs40 (dbl-1(+))*has about twice the amount of *dbl-1 *transcript as LT186 *sma-6(wk7)*, which is consistent with it being overexpressed (Table 1). To validate the results of the microarray experiment, we performed qPCR on 27 genes that were highly up-regulated or down-regulated in our microarray analyses. We compared the ratio of expression of the two experimental genotypes in the qPCR and the microarray experiments to determine if the difference in levels showed the same trend. All but two samples showed the same trend (Table 1).

**Table 1:** Quantitative PCR results.

Gene name	qPCR	Microarray	Agreement
*dbl-1*		2.12	

*sma-6*		4.94	

K07C6.3	0.79	0.41	YES

H12I13.4	0.80	0.80	YES

C25D7.6	0.91	0.28	YES

Y69H2.9	0.19	0.31	YES

C42C1.8	0.37	0.40	YES

T09F5.9	5.62	6.78	YES

F11A6.2	6.79	10.12	YES

Y19D10A.7	5.22	55.30	YES

T10H10.2	0.24	0.16	YES

K02E2.8	2.12	52.80	YES

C29F3.2	5.77	1.48	YES

C29F3.5	9.96	5.095	YES

C05A9.1	1.88	3.77	YES

W09B7.2	5.90	6.42	YES

R02E12.6	0.72	0.33	YES

F44A2.1	0.13	0.12	YES

F01G10.3	0.33	0.25	YES

F21F8.4	0.16	0.44	YES

T21E8.1	8.49	25.64	YES

Y38E10A.5	4.21	12.19	YES

F56A4.2	13.65	11.45	YES

F35C5.9	5.92	5.88	YES

F55G11.4	15.78	5.79	YES

T11F9.4	2.60	3.72	YES

F59A7.2	0.88	0.40	YES

F55B12.4	1.23	0.27	NO

F15E11.10	1.02	9.57	NO

The numeric values shown under each genotype tested represent the relative difference of BW1940 to LT186, with each value normalized to an internal standard. A value of 1 represents identical expression to the standard.

### Regulation of body size genes

How cell and organismal size is controlled is an old question that has been studied at the molecular level in yeast and only sporadically in multicellular organisms [14-16]. Body size is defined at the cellular level by cell number (a result of proliferation and cell death) and cell size [14,17,18]. Besides environmental factors and hormones, TGFβ superfamily signaling pathways have also been clearly implicated in controlling cell and body size in *C. elegans*, *D. melanogaster *[19-21], and in vertebrates [22,23]. Furthermore, because of TGFβ superfamily pathways' roles in cell growth and proliferation, they are commonly associated with uncontrolled cell growth in cancers [24].

This study addresses the mechanisms by which body size is executed in a multicellular organism. *C. elegans *is the only model multicellular organism where the cell number is defined: 959 somatic cells in adult hermaphrodites and 1021 somatic cells in adult males [25]. By removing the cell number variable, our results focus on how cell size differences are achieved through our BMP-like signaling pathway.

Metabolic genes were enriched in our panel of highly up-regulated genes, including energy generation, protein expression, nucleotide synthesis, carbohydrate metabolism, amino acid metabolism and biosynthesis (Table 2). Additionally, we observed a small but consistent up-regulation of ribosomal proteins. Ribosomal proteins have been shown experimentally to be important for cell size regulation in yeast [26], *Drosophila *[27,28], and *Arabidopsis *[29]. Protein synthesis and degradation genes were also enriched (Additional file 3), including ubiquitinylation pathway proteins, suggesting that not only are increased amounts of protein required in the longer animal, but also increased protein turnover machinery.

**Table 2:** Microarray results associated with coregulated gene groups.

Gene List	Representation Factor	P-value	Regulated genes	# in group
**Up-regulated**				

Mount 8	1.9	<1.6e-14	153	803

Mount 20	1.6	<0.007	27	160

Mount 23	4.6	<4.1e-29	67	143

Mount 24	2	<1.8e-04	28	133

Mount 27	4.8	<2.9e-20	43	87

Mount 30	1.9	<0.071	7	36

Mount 31	4.3	<1.5e-05	11	25

Amino Acid Metabolism	1.3	<0.179	14	104

Biosynthesis	1.3	<0.012	65	478

Carbohydrate Metabolism	1.5	<0.040	19	121

Cell Structure	1.4	<0.042	31	219

Cell biology	1.8	<0.077	8	44

Collagen	1.5	<0.16	28	179

Energy Generation	2.1	<3.0e-04	25	117

Intestine	3.6	<0.041	3	8

Nucleotide Synthesis	2.2	<0.004	14	62

Proteases	2.1	<2.6e-04	25	116

Protein Expression	2.2	<9.9e-14	90	390

RNA binding	2.7	<2.6e-13	59	209

**Down-regulated**				

Mount 7	3.1	<2.3e-28	115	810

Mount 11	3.3	<1.2e-24	90	587

DNA Repair Genes	5.5	<3.3e-06	11	44

Germ Line Enriched	3.6	<1.2e-24	83	508

Meiosis	3.8	<0.019	4	23

Mitosis	2.7	<0.003	10	80

Oocyte-enriched	1.8	<0.008	21	258

Specific functional groups were found to be over-represented in our 99.0% significant group using Stanford Microarray Database web tools [57]. A representation factor of 1.0 would be expected in a randomly generated list of genes. Higher values show enrichment for genes in that functional group. P values represent the likelihood of achieving that enrichment by chance. Mount 7 contains germline enriched, oocyte, mitosis, and meiosis genes, Mount 8 contains intestinal genes, proteases, carboxylesterases, lipases, and antibacterial proteins. Mount 11 contains germline enriched, oocyte, meiosis, mitosis, retinoblastoma enriched complex. Mount 20 contains germline enriched, biosynthesis, protein expression, and heat shock genes. Mount 23 contains protein expression and energy generation genes. Mount 24 contains amino acid metabolism, lipid metabolism, and fatty acid oxidation genes. Mount 27 contains amino acid metabolism and energy generation genes. Mount 30 contains protein expression genes, and Mount 31 is not characterized. In addition to Mountains, gene expression is also clustered by biofunctional groups. Number of genes in the group represent the number of genes from several experiments that show coregulation of expression for a particular mountain [57].

Structural genes are also up-regulated by DBL-1 signaling (Additional file 4). Many non-dauer specific collagens and other extracellular matrix genes have increased gene expression at the 95th percentile with increased DBL-1 signaling. Intracellular structural genes, like actins, myosins, and tubulins also show positive changes. However, whether these drive body size changes or are a response to the need for more structural proteins by larger cells remains uncertain.

Germline genes comprise the largest category of genes down-regulated by DBL-1 signaling in our data set. These categories include mitotic and meiotic genes as well as DNA repair genes and oocyte specific genes (Table 2). Recently, the DBL-1 signaling pathway was shown to negatively regulate reproductive aging [30]. Pathway mutants appear to extend the reproductive span of older hermaphrodites by improving oocyte quality, not by affecting ovulation rate, early progeny number, or brood size. The model proposed is that DBL-1 normally modulates reproductive rates in response to environmental stress, and that loss of DBL-1 constitutively extends reproductive aging. Somatic life span was largely independent from germline health span. Our results indicate that the mechanism by which this phenomenon acts is through transcriptional regulation of germline-specific genes. We tested if altered regulation of germline genes affected brood size. To test this idea, we picked single L4 animals to plates and allowed them to lay eggs. The parental hermaphrodites were transferred and the eggs were counted every eight hours until no more eggs were laid. DBL-1 overexpressing animals (BW1940) had an identical brood size (272 eggs on average, n = 10) to wild type. *sma-6 *animals show a significantly smaller brood size (p = .002), averaging only about 122 eggs. This brood size is similar to those of other mutant strains that have loss of DBL-1 pathway gene function [30]. Furthermore, eggs and embryos from mutants in the DBL-1 pathway are of normal size and the gonad from DBL-1 overexpressing animals is not proportionally bigger (our unpublished observations and [31]). Our results suggest that increased DBL-1 pathway signaling does not greatly affect the germline but loss of signaling does, by increasing expression of normally repressed germline-specific genes.

Cell cycle genes appear to be largely unaffected at the transcriptional level by DBL-1 signaling at the L4 stage. Other TGFβ superfamily members have been implicated in cell cycle regulation and cell proliferation, not only during development and homeostasis, but also during cancer progression [24,32]. In *C. elegans*, body size is dissociated from cell proliferation and number; however it is associated with endoreduplication in the polyploid hypodermal cells. Long animals with increased DBL-1 signaling have increased ploidy in hypodermal cells, and small animals with decreased DBL-1 signaling have reduced ploidy at later stages [3,31,33,34]. This indicates that some cell cycle genes are regulated by DBL-1, perhaps post-transcriptionally or at a level that does not reach significance in our analyses. Further, cell cycle genes may be altered at later stages of development. DBL-1 signaling does not affect the organism's maturation time or number of somatic cells, but pathway mutants do have reduced brood sizes, as indicated above. This could be an indication of cell cycle regulation in the adult gonad [4,7,35].

Another similar but distinct published analysis has produced overlapping results. Mochii et al. (1999) screened an arrayed filter of *C. elegans *cDNAs (representing 7584 genes) for differences in regulation between *dbl-1(lf)*, *sma-2(lf)*, *lon-2(lf)*, and wild-type populations of third larval-stage animals [36]. Their results showed 20 genes (22 clones) that were both highly down-regulated in *dbl-1(lf) *and *sma-2(lf) *animals and significantly up-regulated in *lon-2(lf) *animals. Of those 20 genes, we find 14 are also highly regulated in our screen (Additional file 5). Included in this subset are the DBL-1 receptor gene, *sma-6*, and *lon-1*, a downstream transcriptional target of DBL-1 signaling [7,35]. Our microarray data for *lon-1 *indicates there may be regulation, similar to what was previously reported, but variation between the data sets puts this result below the 95% confidence. In our previous study of *lon-1*, we reported a difference in expression of LON-1 protein between *lon-1 *and *sma-6 *of about 30% [35]. This level of change would not be detected with confidence in a microarray experiment.

Taken together these results suggest that the ultimate effects of DBL-1 signaling on body size in *C. elegans *may be accomplished through changes in regulating a broad range of genes involved in metabolism and structure.

### Hedgehog superfamily signaling is a downstream regulator of DBL-1 signaling

We identified three hypodermal *wrt *genes and patched receptor genes in our array. *wrt-1 *and *wrt-8 *were significantly up-regulated, 10-fold and 8-fold respectively. *wrt-4 *was up-regulated but just below statistical significance in our experiments, but was up-regulated significantly in Liang et al (2007), using a different set of TGFβ transducers [37]. Nematodes do not have *hedgehog *genes, but bioinformatic analysis shows there are several genes that have a conserved Hint domain (autoprocessing domain, similar to the intein domain in *hedgehog*) but a different N-terminal ligand domain (similar in size to the *hedgehog *domain, but with no sequence similarity). These genes are called *warthog *to show their relationship to *hedgehog *[38-40]. The three *warthog *genes that are regulated by DBL-1 are exclusively expressed in the hypodermis [38]. All three *warthog *genes are related to each other in both the *wart *and Hint domains, with *wrt-4 *and *wrt-8 *being most similar to each other (~55% identity between *wrt-4 *and *wrt-8 *and about 30% identity between *wrt-1 *and the other two).

We obtained gene knockouts from the nematode genome consortium (National BioResource Project; http://www.shigen.nig.ac.jp/c.elegans/index.jsp) for the three *wrt *genes in order to test the hypothesis that they affect body size. We made and measured double and triple mutant combinations of animals. Measurements of the *wrt *mutants singly and in combination with each other show that they are smaller than wild-type animals (~89%, see Table 3). The double and triple mutant combinations of these three genes do not show a further reduction of body size, suggesting that all three operate in the same pathway. The partial reduction in size observed with the triple mutant, compared to loss of *dbl-1 *pathway function, could be explained by the existence of several other *warthog *genes that show low levels of expression in the hypodermis [37,38]. A Sma body size for the *wrt *genes has also been reported in RNAi experiments [41]. Additional evidence that these genes are linked to body size comes from our injection experiments. Overexpression of *wrt-1 *is mostly lethal, but animals that escaped this terminal phenotype are Sma. Likewise, a partial genomic fragment of *wrt-1 *fused to GFP is also mostly lethal, with escapers presenting a Sma phenotype. A genomic *wrt-8:gfp *fusion (containing part of the ligand domain fused to *gfp*, driven by 2180 bp of *wrt-8 *promoter sequence) was injected and the resulting transgenic animals are Sma. These overexpression phenotypes suggest that proper levels of WRT-1 and WRT-8 are required for normal body size morphology.

**Table 3:** Body lengths of *warthog *mutants.

Genotype	% length of N2	n	P-value
wild type (N2)	100 ± 2	10	

*sma-6(wk7)*	66 ± 4	21	<.001

*wrt-1(tm1417)*	89 ± 4	17	0.290

*wrt-4(tm1911)*	89 ± 4	18	0.014

*wrt-8(tm1585)*	87 ± 4	20	0.080

*wrt-1(tm1417); wrt-4(tm1911)*	85 ± 3	17	0.020

*wrt-1(tm1417); wrt-8(tm1585*	89 ± 4	12	0.036

*wrt-8(tm1585); wrt-4(tm1911)*	85 ± 4	21	0.061

*wrt-1(tm1417); wrt-8(tm1585); wrt-4(tm1911)*	87 ± 4	17	0.036

Body size measurements of single, double, and triple mutant combinations. n represents number of animals measured 48 hours after the L4 stage. The P-value is the probability that the tested strain length is the same as the wild type.

Liang et al (2007) compared expression patterns between *dbl-1(lf)*, *sma-9(lf)*, and wild-type animals [37]. *sma-9 *encodes a predicted co-transcription factor for the DBL-1 pathway Smads [42]. They found 31 genes are commonly regulated by *sma-9 *and *dbl-1*. Only one, *wrt-1*, is down-regulated in both *dbl-1(lf) *and *sma-9(lf) *microarrays relative to the wild type. This supports our microarray results showing significant up-regulation of *wrt-1 *when *dbl-1 *is overexpressed.

While DBL-1 affects the body size of animals living in reproductively favorable conditions, *C. elegans *has another BMP superfamily member, DAF-7, that regulates an alternative life stage called dauer, a facultative diapause that animals enter in response to harsh environmental conditions [43,44]. DBL-1 and DAF-7 use the same Type II receptor, DAF-4. We reasoned that DBL-1 and DAF-7 might use similar but distinct mechanisms or signaling pathways to regulate their distinct effects. To address this, we compared our results to those from a microarray experiment that compared non-dauer larvae at around the L2 molt to same stage (L2d) animals entering dauer due to loss of function of DAF-7 or the DAF-7 Smads DAF-8 and DAF-14 [45]. The dauer analysis showed that *dbl-1 *is down-regulated in dauering animals, and also identifies several genes related to Hedgehog (Hh) by a common Hog domain, as well as Patched (Hh receptor) genes. Consistent with the down-regulation of *dbl-1 *in animals entering dauer, they also found that *wrt-1 *and *wrt-8 *were also down-regulated. Seven patched genes were also significantly down-regulated in the dauer study, while we found another, *ptr-24*, to be 1.3-fold (P = 0.014) up-regulated. This indicates that DBL-1 and DAF-7 are using similar mechanisms (*wrt *signaling pathways) to regulate distinct biological outcomes.

### Regulation of male spicule development

Because we used a hermaphrodite population in our studies, we expected to exclude most genes highly regulated by DBL-1 during male tail development. That is largely the case, since most are expressed male-specifically. One that was identified, *lin-31*, also has roles in hermaphrodite development [46]. LIN-31 is a forkhead transcription factor, which in other systems is a Smad co-factor [9]. LIN-31 is implicated in DBL-1-mediated male tail development, as *lin-31 *mutant males have crumpled spicules like those exhibited by *dbl-1 *pathway mutant males [9]. We show a transcriptional effect of DBL-1 on *lin-31*, as it is 1.5-fold (P = 0.036) up-regulated by pathway signaling. This indirectly supports the hypothesis that DBL-1 acts through LIN-31 in affecting spicule development.

### Regulation of immunity

TGFβ superfamily members play a role in immune responses in mammals [47]. DBL-1 is up-regulated in microarrays analyzing *C. elegans *innate immunity, and *dbl-1(lf) *animals succumb sooner than the wild type to infection by pathogenic bacteria and yeast [5,6,48,49]. While the DBL-1 pathway is required solely in the hypodermis for its body size role, all receptors and Smads are more strongly expressed in the intestine and/or pharynx, primary sites for the *C. elegans *immune response [50]. A plausible explanation for DBL-1 pathway expression in the intestine is that it transcriptionally regulates genes required for an immune response. In our microarray study, animal populations were bleached to not only stage them but also to control for possible contamination responses unrelated to genotype. We identified several families of genes known to be involved in the immune response, including lysozymes, lectins, and lipase, as well as *npr-1 *(Additional file 5) [5,11,12]. Other genes with intestinal expression are also enriched (Table 2).

When we compared our results to data obtained from two other microarray analyses analyzing immune response to pathogenic bacterial infection, we identified a remarkable overlap between their highly regulated genes and a subset of ours [5,11]. Mallo et al. analyzed the *C. elegans *transcriptional response to *S. marcescens *infection [5]. They identified seven genes with an induction of greater than 2-fold, including a lipase, lectins, and lysozymes, which are involved in immune responses in other animals [5,11]. Of those seven, three were identified in our screen as highly up-regulated (Additional file 5). They also found that all of the lysozyme genes they tested (*lys-1, -7, and -8*) were induced in infected animals by microarray and by northern analyses. We found that these three *lys *genes were also highly up-regulated in our microarray, as were *lys-2 *and *lys-9*, which were not represented by cDNAs in the previous study. Wong et al. also identified lipase, lectin, and lysozyme gene up-regulation when they compared animals fed on standard OP50 *E. coli *to pathogenic *E. caratovora*, *E. faecalis*, and *P. luminescens*-fed animals [11]. They also identified aspartyl proteases and saposin as highly up-regulated. These were identified in our analysis as highly up-regulated (Additional file 5). A directed analysis of immune response in *C. elegans *by Alper et al. demonstrated DBL-1 regulation of *lys-1*, *-7*, and *-8 *as well as the lectin *clec-85 *[12].

### Development of a DBL-1 pathway fluorescent reporter

**Figure 1:**
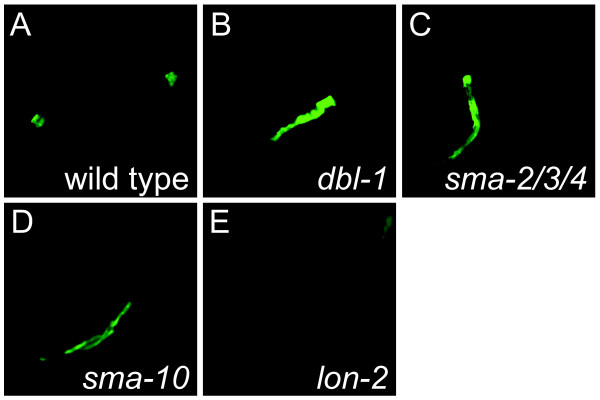
**Expression of the SPP-9P::GFP transcriptional reporter in Sma/Mab pathway mutants**. The SPP-9P::GFP reporter was crossed into various Sma/Mab pathway mutants to determine the effect of the mutation on the expression level of the reporter. All exposures were of equal duration. A) Repression of the SPP-9P::GFP reporter in a wild-type animal shows little expression in the central region of the intestine, B) SPP-9P::GFP expression in a *dbl-1*, C) the triple mutant *sma-2,3,4*, D) *sma-10 *mutants show strong expression throughout the center of the intestine, and E) SPP-9P::GFP expression in a *lon-2 *mutant that overexpresses the pathway shows little expression, similar to the reporter repression seen in wild-type animals.

To create a reporter for DBL-1 signaling, we tested six of the highest regulated genes for efficacy as a reporter for the Sma/Mab pathway (T25C12.2, T09F5.9, F35C5.9, Y38E10A.5, W09B7.2, T10H10.2, F11A6.2). We drove green fluorescent protein (GFP) expression from these genes' promoters and compared GFP expression levels in wild-type and *sma-6(wk7) *animals. We showed that the promoter for an immune-response gene, *spp-9*/saposin (T25C12.2) showed the greatest difference in response to altered DBL-1 pathway levels (0.1-fold regulated, P = 0.002) [51,52]. GFP from the *spp-9 *promoter is weakly expressed in the intestine of wild-type, OP50-fed animals (Fig. 1). However, in the *sma-6(wk7) *background, this promoter is up-regulated, as seen by increased intestinal fluorescence (Fig. 1). This marker exhibited no change when placed in the background of collagen mutants, which affect body size independently of DBL-1 (data not shown). There are a number of putative Smad binding sites in the promoter region of *spp-9*, which suggests it may be a direct target, but binding to these sites has not been validated. This strain helps validate our mutants identified from genetic screens, but also provides a screenable marker for future studies.

## Conclusions

Our results show how DBL-1 pathway signaling in the hypodermis leads to body size changes by regulating transcription of genes involved in metabolism, protein synthesis and degradation, but not significantly by cell cycle genes. We identified a proposed downstream signal transduction pathway in the Hh-related *wrt *signaling pathway, which may relay the DBL-1 pathway signal out of the hypodermis to neighboring cells to regulate body size. We have discovered a mechanism for DBL-1 in the intestinal innate immune response: to promote transcription of many genes directly involved in immunity. A fluorescent biomarker for DBL-1 pathway signaling was generated and will provide the basis for future studies of how DBL-1 signaling is regulated.

## Methods

### *C. elegans *strains

*C. elegans *strains were cultured using standard methods at 20°C [53]. All mutants used in this paper were derived from the wild-type Bristol strain N2. *wrt-1(tm1417)*, *wrt-4 (tm1911)*, and *wrt-8(tm1585) *were isolated by the National BioResource Project http://www.shigen.nig.ac.jp/c.elegans/index.jsp. *rrf-3(pk1426) *is described in [54]. *wrt-1(tm1417) *is created by a deletion that removes 616 bp DNA, encoding a protein truncated after amino acid 116 with a short (15 amino acid) missense transcript thereafter. This transcript removes the C-terminal portion of the *wrt-1 *Wart domain and its Hog domain. *wrt-8(tm1585) *is caused by a 1256 bp deletion and encodes a protein truncated after 32 amino acids with four additional amino acids of missense transcript. This removes most of the *wrt-8 *Wart domain. *wrt-4(tm1911) *is a 912 bp deletion that removes exons 4 and 5. Strain LT186 contains a molecular null of the receptor gene, *sma-6(wk7) *[7]. BW1940 is a strain that contains an integrated *dbl-1*-overexpressing transgene *ctIs40 *(ZC421 cosmid + pTG96 (*sur-5::gfp*)) [4]. Microinjection of DNAs into the gonad syncytia of *C. elegans *hermaphrodites to create transgenic animals was performed by standard microinjection procedures [55,56] and resulted in *wkEx52 [spp-9p::gfp]*, *wkEx65 *[*wrt-8p*::partial *wrt-8:gfp *+ pRF4 (*rol-6(su1006)*)] and *wkEx66 *[*wrt-1p::gfp *+ pRF4(*rol-6(su1006)*)]. Expression of pRF4 was used to select for transgenic animals.

### RNA isolation

A large population of animals was bleached for eggs. The eggs were then allowed to hatch overnight in M9 media without food in order to synchronize the population at L1. Animals were then plated to NGM plates containing OP50 *E. coli*. Animals were scored visually for the L4 stage and washed off plates using M9 or 0.1 M NaCl solution, then pelleted and dissolved with TRIzol^® ^reagent (Invitrogen Life Technologies, Gaithersburg, MD). We chose the L4 stage to analyze because body length differences are apparent at this stage, it is easy to stage multiple populations at this developmental age, and there will be no possible confounding of results by developing embryos, which are present in adults. After several rounds of vortexing and freeze thaw cycles using liquid nitrogen, the solution was extracted using chloroform, leaving an aqueous solution containing the RNA. The RNA was precipitated using isopropanol, and the pellet was then purified using the RNeasy^® ^kit (Qiagen Inc., Valencia, CA).

### Preparation of Labeled Copy RNA

Total RNA was extracted from each sample and prepared for hybridization according to the Affymetrix GeneChip^® ^Expression Analysis Technical Manual (Affymetrix, 2001). Briefly, RNA was extracted from frozen tissue using the RNeasy^® ^Mini kit (Qiagen Inc, Valencia, CA). Sample was further purified and concentrated with an RNeasy MinElute Cleanup column (Qiagen Inc, Valencia, CA). A 200 ng aliquot of each RNA sample was loaded in an RNA 6000 Nano Chip and run on a Bioanalyzer (Agilent Technologies, Palo Alto, CA). The Nano Chip separates the sample via capillary electrophoresis (Agilent Technologies, Palo Alto, CA), and the quality of each sample was determined by evaluating the relative amounts of 28 S and 18 S ribosomal peaks.

Five mg of total RNA was used as a template for complementary DNA (cDNA) synthesis with the Superscript Choice System kit (Invitrogen Life Technologies, Gaithersburg, MD). First strand synthesis was primed with a T7-(dT)_24 _oligonucleotide primer containing a T7 RNA polymerase promoter sequence on the 5' end (Genset Oligos, La Jolla, CA). Second strand products were cleaned with the GeneChip^® ^Sample Cleanup Module (Affymetrix, CA) and used as a template for *in vitro *transcription (IVT) with biotin-labeled nucleotides (Bioarray High Yield RNA Transcript Labeling Kit (Enzo Diagnostics, Farmindale, NY). 20 mg of the product was heated at 94°C for 35 minutes in fragmentation buffer provided with the Cleanup Module (Affymetrix) in order to produce fragments that were 35-200 base pairs in length.

### Array Hybridization

Fragmented samples were submitted to the University of Florida's joint Shands Cancer Center/Interdisciplinary Center for Biotechnology Research (ICBR) Microarray Core Facility (Gainesville, FL). A 15 μg aliquot of fragmented cRNA was hybridized for 16 hr at 45°C to an Affymetrix *C. elegans *GeneChip^®^. After hybridization, each array was stained with a streptavidin-phycoerythrin conjugate, washed (Molecular Probes, Eugene, Oregon), and visualized with a GeneArray™ scanner (Agilent Technologies, Palo Alto, CA). Images were inspected visually for hybridization artifacts. In addition, quality assessment metrics were generated for each scanned image. Microarray core facility staff evaluated these metrics based empirical data from pervious hybridizations and on the signal intensity of internal standards that were present in the hybridization cocktail. Samples that did not pass quality assessment were eliminated from further analyses.

### Generation of Expression Values

Microarray Suite Version 5 software (Affymetrix, Santa Clara, CA) was used to generate .cel files. Probe Profiler™software (v1.3.11) (Corimbia Inc, Berkeley, CA) was used to convert .cel file intensity data into quantitative estimates of gene expression. All expression values were globally scaled to 100 using Probe Profiler™software that was developed specifically for the Affymetrix GeneChip^® ^system. The software identified informative probe pairs, and down-weighted the signal value of probe pairs that were subject to differential cross-hybridization effects or that consistently produced no signal. The software also detected and corrected for saturation artifacts, outliers and chip defects.

In addition to expression levels, Probe Profiler™generated a probability level associated with the genes' presence or absence. Genes not expressed in at least 2 of the 11 samples (p < .05) (BW1940: n = 5 and LT186: n = 6) were considered absent. Absent genes were removed from the data set and not included in further analyses.

### Data Analysis

A modified t-test was performed on the gene expression values (BW1940: n = 5 and LT186: n = 6) with Probe Profiler™(Corimbia Inc., Berkeley, CA). For each analysis, the genes that had a significant treatment effect (p = 0.05, 0.01 or 0.001) were identified. The expression values of these genes were normalized on a gene-by-gene basis by first subtracting from each expression value the mean expression value across all arrays, and then dividing standard deviation of values for that gene. In this way a distribution with mean 0 and standard deviation 1 was created for each gene. Hierarchical clustering, K-Means clustering and PCA was performed on the normalized data and visualized with Gene Linker Gold software. All filtering and normalization was performed with AnalyzeIt Tools, a software package developed by the Interdisciplinary Center for Biotechnology Research (ICBR) at the University of Florida.

### Array Data Submission

Array data has been submitted to the Gene Expression Omnibus with accession number GSE15527 http://www.ncbi.nlm.nih.gov/geo/.

### cDNA and qRT-PCR

cDNA libraries were constructed from the RNA of BW1940 *ctIs40 *and LT186 *sma-6(wk7) *strains using the iScript cDNA synthesis kit (BioRad). SYBR Green PCR reactions were carried out using a Rotor-Gene RG3000 and the IQ SYBR Green supermix (BioRad). Genes of interest were amplified. A standard curve was used to determine accurate comparisons of transcription levels. Each experimental transcript was compared to an internal control (T11G6.1, histidyl-tRNA synthetase), which showed no significant deviation in our microarray data, in order to obtain a relative expression value. Values from three replicates were averaged to determine the overall level of transcription (Table 1).

### Body length measurements

Animals were picked at the L4 stage and photographed as young adults around 48 hours later. Images from individual animals were captured from dissecting microscopes using a QImaging Retiga 1300 cooled color digital camera system and QCapture2 software (Quantitative Imaging Corporation, Burnaby, Canada). Lengths of animals were determined by using Image-Pro Plus measurement software (Media Cybernetics, Inc., Silver Spring, MD).

### Reporter Constructs

Reporter constructs were generated using approximately 3 kb of DNA upstream of the gene of interest. These promoter sequences were amplified by PCR and cloned into the GFP vector pPD95.75 [55]. After sequencing to verify cloning, the plasmids were injected with marker pRF4 into N2 wild-type young adult hermaphrodites using standard DNA transformation techniques [55,56]. Transformed F1 animals were isolated and lines were obtained from transgenic F2 progeny.

## List of abbreviations

BMP: bone morphogenetic protein; L2d: second larval stage entering dauer; L4: fourth larval stage; (lf): loss of gene function; IVT: *in vitro *transcription; TGFβ: Transforming Growth Factor-beta superfamily.

## Authors' contributions

AFR participated in the design of the experiments, performed the wet lab experiments, assisted with the analysis of the data, and helped draft the manuscript. TLG analyzed the microarray data, performed some genetic experiments, and helped draft the manuscript. RJG participated in image acquisition, analyses of the *warthog *mutations, and assisted in the preparation of the manuscript. HW participated in the molecular cloning and analyses of molecular constructs. RWP conceived the study, participated in the design and data analyses, and helped draft the manuscript. All authors have read and approved the final manuscript.

## Supplementary Material

Additional file 1 **Data Summary of regulated genes**. The number of regulated genes scored at confidence intervals of 95%, 99% and 99.9%. Click here for file

Additional file 2 **Genes highly regulated at the 95% confidence interval**. The 99.9% most highly up- and down-regulated are annotated according to WormBase Release WS211. Other annotations primarily come from the Affymetrix microarray spreadsheet. Some manual cross-referencing was required from the Dauer Metabolic Database http://dauerdb.org/ to correlate labels from the microarray with WormBase Release WS211. Click here for file

Additional file 3 **Protein synthesis and degradation genes highly regulated at 95% confidence or above**. A summary list of protein synthesis and degradation genes regulated by the Sma/Mab pathway at the 95% confidence level. Click here for file

Additional file 4 **Structural Genes highly regulated at 95% confidence or above**. A summary list of structural genes regulated by the Sma/Mab pathway at the 95% confidence level. Click here for file

Additional file 5 **Comparisons with similar microarray experiments**. A summary list of comparisons among similar microarray experiments. Mochii et al refers to reference [36], Liang et al refers to reference [37], and Mallo et al refers to reference [5]. Click here for file
