# Supplementary material for: Regulation of genes affecting body size and innate immunity by the DBL-1/BMP-like pathway in Caenorhabditis elegans
Source: BMC Dev Biol. 2010 Jun 7;10:61. doi: 10.1186/1471-213X-10-61 (PMC2894779; doi:10.1186/1471-213X-10-61)
Supplement: Additional file 1 — Data Summary of regulated genes. The number of regulated genes scored at confidence intervals of 95%, 99% and 99.9%. [file 1471-213X-10-61-S1.PDF]

**Stringency**	**99.9%**	**99%**	**95%**
Up-regulated	186	630	1699
Down-regulated	90	300	738
**Total**	**276**	**930**	**2437**
