# Supplementary material for: Regulation of genes affecting body size and innate immunity by the DBL-1/BMP-like pathway in Caenorhabditis elegans
Source: BMC Dev Biol. 2010 Jun 7;10:61. doi: 10.1186/1471-213X-10-61 (PMC2894779; doi:10.1186/1471-213X-10-61)
Supplement: Additional file 2 — Genes highly regulated at the 95% confidence interval. The 99.9% most highly up- and down-regulated are annotated according to WormBase Release WS211. Other annotations primarily come from the Affymetrix microarray spreadsheet. Some manual cross-referencing was required from the Dauer Metabolic Database http://dauerdb.org/ to correlate labels from the microarray with WormBase Release WS211. [file 1471-213X-10-61-S2.PDF]

Mean of BW1940 (DBL-1++)	Mean of LT186 (SMA-6-)	P-value	BW:LT log 2	BW/LT fold difference	Sequence Description	Gene Title/ Description/ KOG
8.5	0.1	0.001	5.9	85.000	R09E12.6	transposon
7.2	0.1	0.038	6	72.000	Y51A2A.1	clec-247 - (C-type LECtin)
55.3	1	0	5.9	55.300	Y19D10A.7	insulin receptor-like
79.2	1.5	0.008	5.7	52.800	K02E2.8	Uncharacterized protein
187.2	7.3	0.01	4.7	25.644	T21E8.1	pgp-6 - (P-GlycoProtein related)
18.2	0.8	0.008	4.5	22.750	C06E1.11	Uncharacterized protein
36	2.7	0.006	3.8	13.333	C41H7.7	clec-3 - (C-type LECtin)
7.6	0.6	0.022	3.7	12.667	T05A8.2	Uncharacterized protein
167.1	13.7	0	3.6	12.197	Y38E10A.5	clec-4 - (C-type LECtin)
38	3.2	0	3.6	11.875	Y38C9B.1	cyp-29A3 - (CYtochrome P450 family)
861.5	75.2	0	3.5	11.456	F56A4.2	lectin (C-type)
22.3	2	0.01	3.5	11.150	ZC376.4	Homeobox domain
198.5	19.6	0	3.3	10.128	F11A6.2	scrm-4 - (SCRaMblase (phospholipid scramblase))
48.1	4.9	0.04	3.3	9.816	Y58A7A.5	Uncharacterized protein
215.5	22.5	0	3.3	9.578	F15E11.10	srbc-15 - (Serpentine Receptor, class BC (class B-like))
383.9	40.9	0.013	3.2	9.386	F35C5.9	clec-66 - (C-type LECtin)
383.9	40.9	0.013	3.2	9.386	ZK1290.12	wrt-1 - (WaRThog (hedgehog-like family))
80	10	0	3	8.000	R01H10.8	cnk-1 - (Connector/eNhancer of KSR) with SAM, PDZ, and PH domains
134.8	17	0.023	3	7.929	K09F5.2	vit-1 (yolk protein gene)
108.9	14.7	0.014	2.9	7.408	C29F3.2	wrt-8 - (WaRThog (hedgehog-like family))
245.7	36.2	0	2.8	6.787	T09F5.9	clec-47 - (C-type LECtin)
131.7	20.5	0	2.7	6.424	W09B7.2	Uncharacterized protein
47.3	7.7	0.008	2.6	6.143	Y43D4A.6	Checkpoint kinase and related serine/threonine protein kinases
257.8	43.8	0	2.6	5.886	R09H10.5	EGF domains
257.8	43.8	0	2.6	5.886	F35C5.9	clec-66 - (C-type LECtin)
404.2	69.7	0	2.5	5.799	F55G11.4	Uncharacterized protein
68.2	13.3	0.001	2.4	5.128	F36G9.12	oac-20 - (O-ACyltransferase homolog)
101.9	20	0.003	2.4	5.095	C29F3.5	clec-230 - (C-type LECtin)
77.7	15.7	0	2.3	4.949	C32D5.2	sma-6 - (SMAll) serine-threonine kinase
28.4	5.8	0.033	2.3	4.897	F08H9.5	clec-227 - (C-type LECtin)
14.2	2.9	0.004	2.3	4.897	F56A12.2	Uncharacterized protein
34.6	7.4	0.004	2.2	4.676	Y69A2AR.25	Uncharacterized protein
20.4	4.4	0.014	2.2	4.636	F14B6.6	Galactosyltransferase

202	45.2	0.026	2.2	4.469	F15E11.12	Uncharacterized protein
35.7	8.5	0.015	2.1	4.200	Y73F8A.1	pkd-2 - (human PKD2 (Polycystic Kidney Disease) related)
12.5	3.1	0.004	2	4.032	K08F9.1	glucose transporter
12.9	3.2	0	2	4.031	Y43F8B.5	scl-21 - (SCP-Like extracellular protein), defense-related protein
38.3	9.6	0	2	3.990	T01C8.3	Uncharacterized protein
63.3	16.1	0	2	3.932	C07G3.2	irg-1 - (Infection Response Gene), predicted riboflavin biosynthesis protein
445.9	115.6	0.037	2	3.857	F15E11.15	Uncharacterized protein
8.1	2.1	0.02	1.9	3.857	F57G4.5	transposon
31.1	8.1	0.012	1.9	3.840	F17B5.1	Thioredoxin, nucleoredoxin and related protein
157.9	41.8	0.003	1.9	3.778	C05A9.1	pgp-5 - (P-GlycoProtein related)
77.1	20.7	0.037	1.9	3.725	T11F9.4	aat-6 - (Amino Acid Transporter)
56	15.2	0	1.9	3.684	F55G11.5	dod-22 - (Downstream Of DAF-16 (regulated by DAF-16))
71.7	20.1	0.01	1.8	3.567	K04C1.4	myosin light chain
9.9	2.8	0.033	1.8	3.536	C41H7.5	Uncharacterized protein
9.7	2.8	0.008	1.8	3.464	F37B1.5	gst-16 - (Glutathione S-Transferase)
8.5	2.5	0.039	1.8	3.400	F40G9.4	btb-1 - (BTB (Broad/complex/Tramtrack/Bric a brac) domain protein)
97.5	29.1	0	1.7	3.351	K08D8.5	Uncharacterized protein
9	2.7	0.018	1.8	3.333	Y39A3B.4	srd-66 - (Serpentine Receptor, class D (delta))
50.9	15.5	0.014	1.7	3.284	K10C2.1	Serine carboxypeptidases (lysosomal cathepsin A)
50.9	15.5	0.014	1.7	3.284	K01A2.3	Uncharacterized protein
102.1	31.3	0	1.7	3.262	F35C5.9	clec-66 - (C-type LECtin)
477.2	146.7	0	1.7	3.253	F01D5.1	Secreted surface protein
437.3	136.1	0	1.7	3.213	F55G11.2	Uncharacterized protein
83.7	26.2	0.01	1.7	3.195	T05E12.7	srh-237 - (Serpentine Receptor, class H)
14.6	4.6	0.004	1.7	3.174	W09B7.1	C2H2-type Zn-finger protein
24.1	7.6	0	1.7	3.171	C18D4.6	Uncharacterized protein
40.2	12.7	0.006	1.7	3.165	F35C5.6	clec-63 - (C-type LECtin)
40.2	12.7	0.006	1.7	3.165	F35C5.8	clec-65 - (C-type LECtin)
40.2	12.7	0.006	1.7	3.165	K01A2.3	Uncharacterized protein
186.4	59	0.014	1.7	3.159	F48G7.5	Secreted surface protein
56.2	17.8	0	1.7	3.157	K08D8.5	Uncharacterized protein
308.4	98.1	0.006	1.7	3.144	B0365.6	clec-41 - (C-type LECtin)
9.7	3.1	0.011	1.6	3.129	K01G12.1	transposon
43.8	14	0	1.7	3.129	F52F10.4	oac-32 - (O-ACyltransferase homolog)
41.5	13.4	0.002	1.6	3.097	F36D3.9	cpr-2 - (Cysteine PRotease related)
98.3	32.2	0.018	1.6	3.053	Y46C8AL.2	clec-174 - (C-type LECtin)
18.3	6	0.021	1.6	3.050	C32B5.13	Cysteine proteinase Cathepsin L
10.2	3.4	0.029	1.6	3.000	C49G7.5	irg-2 - (Infection Response Gene)

16.1	5.4	0	1.6	2.981	C39B5.7	fbxa-12 - (F-box A protein)
61.6	20.7	0	1.6	2.976	Y70C5A.2	Uncharacterized protein
10.6	3.6	0.038	1.6	2.944	Y34D9A.2	Uncharacterized protein
851.4	290.6	0	1.6	2.930	C32H11.12	dod-24 - (Downstream Of DAF-16 (regulated by DAF-16))
269	91.9	0.048	1.6	2.927	F41E6.9	Protein involved in vacuolar protein sorting
40.3	13.9	0	1.5	2.899	Y38E10A.4	clec-8 - (C-type LECtin)
111.2	38.4	0	1.5	2.896	T07D4.3	rha-1 - (RNA HelicAse)
38.1	13.2	0.004	1.5	2.886	K12C11.3	Uncharacterized protein
532	184.7	0	1.5	2.880	K03A1.5	sur-5 - (SUppressor of activated let-60 Ras), acetyl-coenzyme A synthetase
13.5	4.7	0.007	1.5	2.872	F07G6.8	Uncharacterized protein
52.3	18.3	0.002	1.5	2.858	T16G12.1	Puromycin-sensitive aminopeptidase and related aminopeptidases
494.4	173.8	0.007	1.5	2.845	M02F4.7	clec-265 - (C-type LECtin)
37.9	13.5	0	1.5	2.807	K08D8.4	Uncharacterized protein
360.3	128.8	0.001	1.5	2.797	F56D6.2	clec-67 - (C-type LECtin)
93.8	33.6	0.006	1.5	2.792	C29F9.3	Uncharacterized protein
178.6	64.9	0.001	1.5	2.752	C03H5.1	clec-10 - (C-type LECtin)
8.2	3	0.041	1.5	2.733	4R79.2	GTP-binding protein SEC4, small G protein superfamily, and related Ras family G[illegible]
253.9	93.5	0	1.4	2.716	F08H9.7	clec-56 - (C-type LECtin)
9.5	3.5	0.022	1.4	2.714	R11G11.14	Triglyceride lipase-cholesterol esterase
96.9	35.8	0.001	1.4	2.707	C46F2.1	Uncharacterized protein
8.1	3	0.043	1.5	2.700	T24A6.13	srbc-68 - (Serpentine Receptor, class BC (class B-like))
226.2	84	0.002	1.4	2.693	F19B2.5	Helicase-like transcription factor HLTF/DNA helicase RAD5, DEAD-box superfami
54.8	20.6	0.05	1.4	2.660	Y38E10A.16	nspe-5 - (Nematode Specific Peptide family, group E)
8.5	3.2	0.039	1.4	2.656	T08H4.2	Uncharacterized protein
8.5	3.2	0.043	1.4	2.656	F49F1.10	Galectin, galactose-binding lectin
28.9	10.9	0.039	1.4	2.651	F07E5.4	Uncharacterized protein
8.2	3.1	0.047	1.4	2.645	Y48G1BM.6	Uncharacterized protein
49.8	18.9	0.011	1.4	2.635	F10G2.3	clec-7 - (C-type LECtin)
9.4	3.6	0.032	1.4	2.611	F14B6.6	Galactosyltransferase
26.1	10	0.046	1.4	2.610	C10C5.2	Uncharacterized protein
97.9	37.6	0.002	1.4	2.604	ZC317.5	srg-68 - (Serpentine Receptor, class G (gamma))
21.3	8.2	0	1.4	2.598	Y39G8B.5	TANK binding protein kinase TBK1
16.6	6.4	0.001	1.4	2.594	C41H7.1	Uncharacterized protein
146.2	56.4	0.004	1.4	2.592	B0353.1	Uncharacterized protein
20.4	7.9	0	1.4	2.582	C16C4.5	math-15 - (MATH (meprin-associated Traf homology) domain containing)
51.4	20	0.044	1.4	2.570	C53D5.5	gamma-glutamyltranspeptidase
47.6	18.8	0	1.3	2.532	F37C12.10	Uncharacterized protein
305.2	120.9	0.004	1.3	2.524	F56D6.1	clec-68 - (C-type LECtin)

337.5	134.1	0.001	1.3	2.517	K07E8.3	sdz-24 - (SKN-1 Dependent Zygotic transcript), single-stranded DNA-binding replic
73.3	29.3	0.001	1.3	2.502	Y38E10A.14	Uncharacterized protein
14.4	5.8	0.012	1.3	2.483	Y71A12B.2	Uncharacterized protein
133.2	53.8	0.012	1.3	2.476	T24E12.1	Uncharacterized protein
18.3	7.4	0.028	1.3	2.473	B0348.2	Uncharacterized protein
195.4	79.2	0.001	1.3	2.467	C03H5.1	clec-10 - (C-type LECtin)
18.7	7.6	0.046	1.3	2.461	C49A1.4	eya-1 - (EYA (Drosophila eyes absent) homolog), haloacid dehalogenase-like hydr
36.9	15.1	0.04	1.3	2.444	F44D12.9	Uncharacterized protein
13.4	5.5	0.034	1.3	2.436	R13H4.1	nph-4 - (NePHronophthisis (human kidney disease) homolog)
82.5	33.9	0.018	1.3	2.434	T28D6.2	tba-7 - (TuBulin, Alpha)
454.3	186.7	0.003	1.3	2.433	T05E12.6	Uncharacterized protein
222.6	91.5	0.014	1.3	2.433	C08E3.1	Uncharacterized protein
17.5	7.2	0	1.3	2.431	T08E11.4	math-41 - (MATH (meprin-associated Traf homology) domain containing)
339.2	140.7	0	1.3	2.411	F40F8.7	pqm-1 - (ParaQuat (Methylviologen) responsive)
20.9	8.7	0.001	1.3	2.402	K02E7.2	transposon
235.3	98	0	1.3	2.401	T16G12.1	Puromycin-sensitive aminopeptidase and related aminopeptidase
13.2	5.5	0.004	1.3	2.400	F36G9.14	fbxa-99 - (F-box A protein)
24.7	10.3	0	1.3	2.398	F53F1.6	Uncharacterized protein
23.1	9.7	0	1.3	2.381	C33D12.2	Predicted membrane protein, contains two CBS domains
17.8	7.5	0.001	1.2	2.373	W04A8.6	Uncharacterized protein
735.6	310.3	0	1.3	2.371	F37B4.7	folt-2 - (FOLate Transporter family)
67.2	28.5	0.003	1.2	2.358	F54B11.11	Uncharacterized protein
54.4	23.1	0.005	1.2	2.355	T04F8.7	Uncharacterized protein
23.5	10	0	1.2	2.350	Y38E10A.4	clec-8 - (C-type LECtin)
27.9	11.9	0	1.2	2.345	ZK896.5	Uncharacterized protein
54.6	23.3	0.001	1.2	2.343	Y46C8AL.17	clec-71 - (C-type LECtin)
893	381.3	0	1.2	2.342	Y22F5A.5	lys-2 - (LYSozyme)
31.1	13.3	0	1.2	2.338	R03A10.3	Molybdenum cofactor sulfurase
226.2	96.9	0.005	1.2	2.334	K10D11.1	dod-17 - (Downstream Of DAF-16 (regulated by DAF-16))
10.7	4.6	0.018	1.2	2.326	T03E6.5	srh-269 - (Serpentine Receptor, class H)
14.6	6.3	0.03	1.2	2.317	Y48A6C.1	predicted pseudogene
16.2	7	0.034	1.2	2.314	F35C5.7	clec-64 - (C-type LECtin)
16.6	7.2	0.006	1.2	2.306	F35E12.2	Uncharacterized protein
10.6	4.6	0.023	1.2	2.304	F39H12.2	Uncharacterized protein
1091.9	475.5	0	1.2	2.296	C14C6.5	Uncharacterized protein
389.3	170	0.017	1.2	2.290	F55A12.7	apm-1 - (AdaPtin, Mu/medium chain (clathrin associated complex))
450.9	197	0	1.2	2.289	Y105C5B.15	Purple acid phosphatase
227.6	99.5	0.013	1.2	2.287	K01A2.3	Uncharacterized protein

227.6	99.5	0.013	1.2	2.287	C08E3.13	Uncharacterized protein
15.3	6.7	0.005	1.2	2.284	T04F3.2	KAZAL and TY domains
419	183.5	0.018	1.2	2.283	F37B4.2	ifc-1 - (Intermediate Filament, C)
13.2	5.8	0.048	1.2	2.276	H02K04.1	lec-229 - (C-type LECtin)
61	26.9	0	1.2	2.268	ZK856.5	Uncharacterized protein
30.1	13.3	0.009	1.2	2.263	C41H7.7	clec-3 - (C-type LECtin)
13.8	6.1	0.008	1.2	2.262	Y39G8B.2	Aldo/keto reductase family proteins
1406.9	622	0	1.2	2.262	C14C6.5	Uncharacterized protein
346.6	153.7	0	1.2	2.255	K01A2.3	Uncharacterized protein
346.6	153.7	0	1.2	2.255	F35C5.8	clec-65 - (C-type LECtin)
47.2	21	0	1.2	2.248	Y69A2AR.13	Uncharacterized protein
11	4.9	0.021	1.2	2.245	F28C12.7	sra-22 - (Serpentine Receptor, class A (alpha))
262.7	117.6	0.013	1.2	2.234	Y105C5A.13	Uncharacterized protein
23.2	10.5	0.046	1.2	2.210	Y22D7AR.12	Protein tyrosine phosphatase, contains WSN domain
11.7	5.3	0.032	1.1	2.208	C07A12.5	spr-3 - (Suppressor of PResenilin defect)
64.7	29.4	0.004	1.1	2.201	K11E4.2	Src homology domain 2
35.1	16	0	1.1	2.194	Y51H1A.2	RUN domain-containing protein
44.3	20.2	0.043	1.1	2.193	T09H2.1	cyp-34A4 - (CYtochrome P450 family)
235.6	107.6	0	1.1	2.190	F07H5.9	pho-13 - (intestinal acid PHOsphatase)
53.2	24.3	0	1.1	2.189	T05F1.11	Thioredoxin, nucleoredoxin and related protein
59.9	27.4	0.03	1.1	2.186	H41C03.3	Branching enzyme
146	66.8	0	1.1	2.186	F57A10.1	str-9 - (Seven TM Receptor)
33	15.1	0	1.1	2.185	F22A3.5	ceh-60 - (C. Elegans Homeobox)
16.8	7.7	0.001	1.1	2.182	F19C7.4	lysosomal carboxypeptidase
142.5	65.4	0.006	1.1	2.179	K08D8.3	Uncharacterized protein
12.4	5.7	0.03	1.1	2.175	T01B6.1	Uncharacterized protein
232.1	106.9	0.028	1.1	2.171	C01F6.6	tag-60 - ortholog of Drosophila SIP1 and of human PDZK1
10.2	4.7	0.035	1.1	2.170	F15A4.8	chitinase
14.1	6.5	0.021	1.1	2.169	F22E5.4	sri-47 - (Serpentine Receptor, class I)
326.2	150.4	0.001	1.1	2.169	F49E11.10	scl-2 - (SCP-Like extracellular protein), testis-specific protein TPX-1 like
235.7	108.9	0.026	1.1	2.164	M01H9.1	trx-3 - (ThioRedoXin)
26.9	12.5	0.037	1.1	2.152	W03A5.6	Uncharacterized protein
143.1	66.6	0.001	1.1	2.149	ZC190.1	cln-3.3 - (human CLN (neuronal ceroid lipofuscinosis) related)
9.6	4.5	0.049	1.1	2.133	C39E6.6	npr-1 - (NeuroPeptide Receptor family)
30.7	14.4	0.001	1.1	2.132	Y41D4B.17	Uncharacterized protein
83.1	39	0	1.1	2.131	ZK370.2	sma-2 - (SMAll), Smad protein
351.3	165.1	0.009	1.1	2.128	T19D12.1	Uncharacterized protein
47	22.1	0.001	1.1	2.127	T25F10.2	dbl-1 - (DPP/BMP-Like), TGF-beta-like growth factor

66.1	31.1	0.002	1.1	2.125	ZK896.5	Uncharacterized protein
18.8	8.9	0.012	1.1	2.112	Y45G12C.9	
45.2	21.4	0.001	1.1	2.112	Y38H6C.5	Zinc finger, CCHC class
158.8	75.2	0	1.1	2.112	M01F1.5	glucose transport protein
40.4	19.2	0.002	1.1	2.104	F35C8.2	
18.3	8.7	0.012	1.1	2.103	AH9.1	transmembrane domain of family 1 of G-protein coupled receptors
111.8	53.4	0.03	1.1	2.094	Y58A7A.3	
140.9	67.3	0.004	1.1	2.094	D2045.8	TNF-alpha induced Protein B12
58.6	28	0	1.1	2.093	F26D10.12	Lectin C-type domain
177.3	84.8	0.007	1.1	2.091	C30G12.2	Alcohol dehydrogenase
28.9	13.9	0	1.1	2.079	T09F5.11	
47.6	23	0.029	1.1	2.070	K05F6.10	
169.9	82.3	0.015	1.1	2.064	F46C3.3	
88.3	42.8	0.002	1.1	2.063	C45G7.3	
19.7	9.6	0.001	1	2.052	F55G11.3	
374.2	182.6	0.032	1	2.049	F45H11.2	ubiquitin
12.7	6.2	0.014	1	2.048	F38E11.4	
728.8	356.7	0.005	1	2.043	T10H9.5	ABC transporter
738.4	361.9	0.023	1	2.040	T05E12.5	
24	11.8	0	1	2.034	C11G10.2	
35.1	17.3	0.036	1	2.029	Y9C9A.8	
219.6	108.4	0.011	1	2.026	F35E8.8	Glutathione S-transferases.
178.7	88.4	0.009	1	2.021	M28.4	
32.5	16.1	0.001	1	2.019	F57G4.6	
43.8	21.7	0	1	2.018	C11E4.5	
24.6	12.2	0.02	1	2.016	C05B5.2	
12.5	6.2	0.017	1	2.016	R09D1.11	chitinase
146.1	72.9	0.017	1	2.004	F19C7.2	lysosomal carboxypeptidase
59.3	29.6	0.02	1	2.003	Y56A3A.27	DNA topoisomerase III
34.4	17.2	0.002	1	2.000	C35861_rc	
18	9	0.002	1	2.000	C52E2.5	
17.4	8.7	0.047	1	2.000	Y43F8C.6	
168.3	84.2	0.04	1	1.999	F01D5.2	
494.5	247.5	0.001	1	1.998	F23B2.11	lysosomal carboxypeptidase
18.7	9.4	0.021	1	1.989	R11G11.12	zinc finger protein
40.7	20.5	0.004	1	1.985	W07G1.4	
13.5	6.8	0.012	1	1.985	Y26D4A.10	
88.6	44.7	0.013	1	1.982	F46B3.8	

64.8	32.7	0.009	1	1.982	C01B4.6	aldose 1-epimerase
74.5	37.6	0.005	1	1.981	C08F11.13	
72.9	36.8	0.006	1	1.981	F57G4.5	
18.4	9.3	0.001	1	1.978	Y60A3A.10	
14	7.1	0.012	1	1.972	T07H3.6	
740.2	375.7	0.002	1	1.970	ZK6.10	
172.2	87.5	0.001	1	1.968	C14C6.2	
24.4	12.4	0.001	1	1.968	C16C4.4	
17.1	8.7	0.009	1	1.966	T28A11.11	glutathione S-transferase
47.5	24.2	0.013	1	1.963	F52B11.6	ins-34
15.5	7.9	0.016	1	1.962	ZK666.1	
146.9	75.1	0	1	1.956	K05F1.10	
253.1	129.4	0.001	1	1.956	C35D10.14	C-type lectin domain
12.9	6.6	0.019	1	1.955	F49C5.2	
32.8	16.8	0.008	1	1.952	T01B4.1	potassium channel protein
18.1	9.3	0.033	1	1.946	C02B4.1	TSP type-1 repeats (13)
48.6	25	0.002	1	1.944	Y19D10A.16	
17.1	8.8	0.016	1	1.943	C06E8.1	
1810.4	932	0.007	1	1.942	F15E11.14	
108.4	56	0	1	1.936	T28D6.7	
68.1	35.2	0	1	1.935	C01B9.1	
108.8	56.3	0.013	1	1.933	Y39G8B.7	
987.2	511.5	0	1	1.930	Y94H6A.D	
221.2	114.8	0.011	1	1.927	F08A8.2	Acyl-coenzyme A oxidase, peroxisomal (palmitoyl-CoA oxidase (AOX))
43	22.4	0.001	0.9	1.920	F44F1.5	
11.1	5.8	0.046	0.9	1.914	F40F11.4	
39	20.4	0.009	0.9	1.912	F48B9.1	
32.1	16.8	0.014	0.9	1.911	Y65B4A.B	
316.6	165.7	0	0.9	1.911	T10B5.7	lipase
34.9	18.3	0.048	0.9	1.907	Y34B4A.E	
13.9	7.3	0.015	0.9	1.904	K01A2.7	col-69 - (COLlagen)
21.5	11.3	0.01	0.9	1.903	M02B1.1	srf-3 - (SuRFace antigenicity abnormal)
21.1	11.1	0.001	0.9	1.901	Y39G8C.1	
102	53.7	0.003	0.9	1.899	W03D8.8	
216.6	114.3	0.001	0.9	1.895	Y57G11C.24	eps-8 - (EPS (human endocytosis) related) epidermal growth factor receptor kinas
33.5	17.7	0	0.9	1.893	T08G3.3	
77.7	41.1	0.001	0.9	1.891	E02H9.5	glycosyl hydrolase
58.2	30.8	0.002	0.9	1.890	C10A4.4	

133.9	71.1	0.022	0.9	1.883	Y49C4A.8	
15.4	8.2	0.012	0.9	1.878	Y48A6B.4	fipr-17
90.6	48.3	0.001	0.9	1.876	F55C12.3	
950.2	506.8	0.006	0.9	1.875	ZK6.11	
84.9	45.3	0	0.9	1.874	F42G4.5	
55.8	29.8	0.032	0.9	1.872	Y17G7B.11	
21.9	11.7	0.006	0.9	1.872	F48F5.4	
159.5	85.3	0.003	0.9	1.870	F11C1.3	membrane glycoprotein
25	13.4	0.017	0.9	1.866	Y54G9A.4	
94.4	50.6	0.014	0.9	1.866	T17A3.8	
32.4	17.4	0.039	0.9	1.862	DC2.4	
54	29	0.003	0.9	1.862	Y54G2A.Q	
12.8	6.9	0.027	0.9	1.855	Y110A2AL.5	
253.4	136.6	0.001	0.9	1.855	W07G1.3	
49.5	26.7	0.008	0.9	1.854	Y69H2.13	
41.7	22.5	0.01	0.9	1.853	Y26D4A.2	
126.8	68.7	0.014	0.9	1.846	T11B7.2	
44.1	23.9	0.01	0.9	1.845	Y51H4A.2	
26	14.1	0	0.9	1.844	H05C05.1	
60.1	32.6	0.011	0.9	1.844	Y45G12C.3	
45.5	24.7	0.005	0.9	1.842	C06B3.2	
22.4	12.2	0.042	0.9	1.836	C48A7.1	
27.9	15.2	0	0.9	1.836	K03B8.2	zinc metalloprotease
42	22.9	0.012	0.9	1.834	M01G5.4	
100.5	54.8	0.011	0.9	1.834	C32F10.4	
92.4	50.4	0.006	0.9	1.833	Y57A10A.14	
56.6	30.9	0.006	0.9	1.832	K09E9.1	
179	98	0.015	0.9	1.827	T06A1.5	
367.8	201.4	0.004	0.9	1.826	Y60A3A.18	
561.8	307.7	0	0.9	1.826	EGAP2.3	pho-1- lysosomal and prostatic acid phosphatases
32.3	17.7	0.002	0.9	1.825	H23L24.5	pme-4 - (Poly(ADP-ribose) Metabolism Enzyme)
23.9	13.1	0.005	0.9	1.824	C10A4.5	
689.3	378	0.002	0.9	1.824	ZK6.7	lipase
47.8	26.3	0.003	0.9	1.817	M162.2	
92.8	51.1	0.004	0.9	1.816	T12B5.10	
19.6	10.8	0.003	0.9	1.815	C25D7.4	
71.5	39.4	0.025	0.9	1.815	T04B8.4	
22.5	12.4	0.002	0.9	1.815	T06E6.4	

104.3	57.5	0.016	0.9	1.814	AU116301_rc	
31	17.1	0.004	0.9	1.813	C31A11.5	
114.2	63	0	0.9	1.813	F37C4.6	phytoene desaturase
94.2	52	0.012	0.9	1.812	K07C11.8	
1377.4	762.6	0.021	0.9	1.806	Y19D10B.A	
12.1	6.7	0.043	0.9	1.806	W06G6.7	
13	7.2	0.038	0.9	1.806	H38K22.4	
89.9	49.8	0.001	0.9	1.805	W05E10.1	multidrug resistance protein, Predicted transporter/transmembrane protein
21.3	11.8	0.011	0.9	1.805	C52E2.4	
136.6	75.7	0.042	0.9	1.804	F53C11.1	
75.5	41.9	0.02	0.9	1.802	Y25C1A.2	
202.6	112.5	0	0.9	1.801	C01G10.9	Initiation factor 2 subunit
31.5	17.5	0.003	0.9	1.800	T08E11.3	
19.6	10.9	0.003	0.8	1.798	W03D2.6	
16.7	9.3	0.036	0.9	1.796	K02E7.1	
58.7	32.7	0.009	0.9	1.795	ZK666.3	Lectin C-type domain short and long forms, von Willebrand factor type A domain
14	7.8	0.032	0.9	1.795	E03H4.8	beta' coatomer protein like
184.1	102.7	0.006	0.8	1.793	Y116F11B.3	peptidase
110.9	61.9	0.014	0.8	1.792	F54B8.4	
22.9	12.8	0.001	0.8	1.789	Y119D3B.B	
32.9	18.4	0.008	0.8	1.788	H14E04.4	
369.4	206.6	0.022	0.8	1.788	F55G11.8	
242.7	135.8	0.013	0.8	1.787	C23H4.1	
65.4	36.6	0.006	0.8	1.787	F49E12.10	C-4 sterol methyl oxidase
33.4	18.7	0.011	0.8	1.786	C16H3.4	
79.8	44.7	0.005	0.8	1.785	E04F6.7	Ribitol dehydrogenase
20.7	11.6	0.003	0.8	1.784	F42A8.1	
99.7	55.9	0.003	0.8	1.784	F39E9.11	btb-17 - (BTB (Broad/complex/Tramtrack/Bric a brac) domain protein)
73.1	41	0.003	0.8	1.783	Y38H6C.13	
61.5	34.5	0.006	0.8	1.783	C70896	
746.2	418.7	0.02	0.8	1.782	K07E3.3	methylenetetrahydrofolate dehydrogenase
58.8	33	0.028	0.8	1.782	C29F3.6	7 transmembrane receptor (rhodopsin family)
15.5	8.7	0.015	0.8	1.782	F31B12.3	
934.1	524.7	0.011	0.8	1.780	ZK6.10	dod-19 - (Downstream Of DAF-16 (regulated by DAF-16))
40.2	22.6	0	0.8	1.779	C39E6.1	
12.8	7.2	0.037	0.8	1.778	Y111B2A.4	
361.9	203.6	0.021	0.8	1.778	Y49E10.18	
429.4	241.6	0.006	0.8	1.777	K12C11.1	peptidase D ortholog

27.9	15.7	0	0.8	1.777	T01G6.4	zinc finger protein
23.8	13.4	0.005	0.8	1.776	F14F9.2	
80.1	45.1	0.002	0.8	1.776	F11A6.1A	kpc-1 - (Kex-2 Proprotein Convertase family) Furin like serine protease
76.5	43.1	0.021	0.8	1.775	ZK550.2	
62.6	35.3	0.001	0.8	1.773	F57G4.3	
174.3	98.3	0.002	0.8	1.773	AU115660_rc	
366	207.3	0.003	0.8	1.766	C01B10.6	
66.2	37.5	0.003	0.8	1.765	Y71A12B.7	
53.3	30.2	0	0.8	1.765	H02F09.3	
549.3	311.7	0.001	0.8	1.762	Y119D3B.21	
20.6	11.7	0.015	0.8	1.761	C24B9.11	
23.4	13.3	0.008	0.8	1.759	R12E2.8	
49.4	28.1	0.009	0.8	1.758	Y39G10AR.D	
616.5	350.9	0.008	0.8	1.757	AV199878_rc	
434.5	247.5	0.003	0.8	1.756	C17H12.8	
137.8	78.6	0.001	0.8	1.753	ZK131.11	
52.9	30.2	0.034	0.8	1.752	F20G2.5	
28.2	16.1	0	0.8	1.752	Y39A3B.1	
214.9	122.8	0.036	0.8	1.750	C12D12.1	
55.1	31.5	0.03	0.8	1.749	Y37D8A.4	
125.2	71.6	0.012	0.8	1.749	AV183046_rc	
120.1	68.7	0	0.8	1.748	Y60A3A.18	
13.1	7.5	0.037	0.8	1.747	Y34D9A.H	
34.9	20	0.005	0.8	1.745	T05B4.6	
24.6	14.1	0.009	0.8	1.745	Y46H3B.2	
660.7	378.7	0	0.8	1.745	C17H12.8	
14.3	8.2	0.025	0.8	1.744	C53377_rc	
51.6	29.6	0	0.8	1.743	F40G9.5	
487.9	279.9	0.001	0.8	1.743	H34I24.2	
34.5	19.8	0.002	0.8	1.742	F07C6.2	
351.7	201.9	0.009	0.8	1.742	F46G10.6	Helix-loop-helix DNA-binding domain
126.8	72.8	0.001	0.8	1.742	C18C4.3	UDP-glucuronosyltransferase
13.4	7.7	0.035	0.8	1.740	C17A2.3	
230.3	132.5	0	0.8	1.738	F35C5.5B	clec-62 - (C-type LECtin)
14.6	8.4	0.022	0.8	1.738	F20H11.6	
17.2	9.9	0.011	0.8	1.737	T20B3.13	clec-40 - (C-type LECtin)
48.8	28.1	0	0.8	1.737	F22D6.12	
25.7	14.8	0.001	0.8	1.736	F58E1.11	

60.7	35	0.023	0.8	1.734	AV199377_rc	
13.7	7.9	0.038	0.8	1.734	Y38E10A.19	
22.7	13.1	0.006	0.8	1.733	ZK250.2	
160.1	92.4	0.007	0.8	1.733	F23H12.1	synaptobrevin like
91.3	52.7	0.006	0.8	1.732	C50F7.5	
88.7	51.2	0.008	0.8	1.732	C49A9.2	
19.4	11.2	0.034	0.8	1.732	F58E2.1	
47.8	27.6	0.006	0.8	1.732	Y43H11AL.C	Y43H11AL.2 and Y43H11AL.3 (pqn-85)
31	17.9	0	0.8	1.732	R10E8.6	
30.8	17.8	0.001	0.8	1.730	F53F4.9	7TM chemoreceptor (srd-family)
53.8	31.1	0.003	0.8	1.730	T16A9.1	
767.7	443.8	0.023	0.8	1.730	K12C11.2	smo-1 - (SUMO (ubiquitin-related) homolog)
100.5	58.1	0.001	0.8	1.730	Y47D7A.14	
172.4	99.7	0.006	0.8	1.729	C34F6.5	
78.9	45.7	0.003	0.8	1.726	R03G5.5	glutathione peroxidase
20	11.6	0.02	0.8	1.724	R03G8.3	
130	75.4	0	0.8	1.724	T04F3.4	
148.9	86.4	0.024	0.8	1.723	M28.8	
296	171.8	0.011	0.8	1.723	W04C9.1	ABC transporter
14.3	8.3	0.028	0.8	1.723	F40H7.8	
592.3	344.1	0.03	0.8	1.721	M6.1	
22.7	13.2	0.011	0.8	1.720	T21B4.9	7TM receptor
14.1	8.2	0.032	0.8	1.720	R07C3.6	
21.3	12.4	0.003	0.8	1.718	H08J19.1	
139.6	81.3	0.007	0.8	1.717	F21D5.3	laccase like copper oxidase
26.6	15.5	0.001	0.8	1.716	F09E10.1	
470.7	274.4	0.004	0.8	1.715	F40F9.9	
239.7	140	0.001	0.8	1.712	AU114038_rc	
24.3	14.2	0.002	0.8	1.711	F40G9.10	
30.8	18	0.001	0.8	1.711	B0212.5	olfactory channel
23.9	14	0.023	0.8	1.707	Y110A2AL.10	
158.3	92.8	0.007	0.8	1.706	E02H9.3	
115	67.5	0	0.8	1.704	F56D5.6	
119.6	70.2	0.041	0.8	1.704	K06A9.1A	
54.5	32	0.008	0.8	1.703	T22H6.3	
1498.7	880	0.011	0.8	1.703	K09F5.3	
59.4	34.9	0.001	0.8	1.702	C45G7.2	
32.5	19.1	0.002	0.8	1.702	T27C4.4A	

104.3	61.3	0.003	0.8	1.701	F22E5.1	
30.1	17.7	0.008	0.8	1.701	Y46H3B.2	
20.4	12	0.009	0.8	1.700	C33E10.10	
116.9	68.8	0.033	0.8	1.699	AU113632_rc	
38.2	22.5	0.019	0.8	1.698	Y41D4B.M	
39.7	23.4	0.031	0.8	1.697	F55C12.7	tag-234
54.9	32.4	0.002	0.8	1.694	F22D6.11	
29.3	17.3	0.025	0.8	1.694	C53B4.1	transport protein
64.5	38.1	0.003	0.8	1.693	F08F1.3	
299.8	177.1	0.03	0.8	1.693	C54D10.1	cdr-2 - (CaDmium Responsive) Glutathione S-transferase
104.1	61.5	0.017	0.8	1.693	K02B12.4	
467.4	276.2	0.022	0.8	1.692	C02A12.4	lys-7 - (LYSozyme)
18.6	11	0.021	0.8	1.691	C40A11.9	
21.8	12.9	0.004	0.8	1.690	Y23H5A.2	
32.6	19.3	0.011	0.8	1.689	C50F7.2	
22.6	13.4	0.007	0.8	1.687	Y38H6C.14	
26.3	15.6	0.003	0.8	1.686	F58H7.7	
25.1	14.9	0.007	0.8	1.685	R134.1	guanylate cyclase
175.7	104.3	0	0.8	1.685	F25B4.8	
214.4	127.3	0	0.8	1.684	Y50C1A.1	
33.3	19.8	0.011	0.8	1.682	F55G1.7	
33.3	19.8	0.032	0.8	1.682	Y18D10A.5	Eukaryotic protein kinase domain
89.4	53.2	0.004	0.8	1.680	C32D5.6	
21.5	12.8	0.046	0.8	1.680	Y38E10A.1	
22.5	13.4	0.003	0.7	1.679	K09F6.6	
348.4	207.5	0	0.8	1.679	Y46G5A.19	
61.1	36.4	0.002	0.8	1.679	ZK520.2	
124.7	74.3	0.04	0.8	1.678	K06A9.1B	
585.6	349	0.036	0.8	1.678	T01D3.6B	EGF-like domain, Fibrinogen beta and gamma chains, C-terminal globular domain
34.2	20.4	0	0.7	1.676	ZC204.2	homeobox protein
17.6	10.5	0.013	0.8	1.676	C31B8.3	
92	54.9	0.007	0.8	1.676	AV202093	
98.3	58.7	0.001	0.7	1.675	K03H1.5	Transmembrane and sushi domain
47.7	28.5	0.006	0.7	1.674	C36B1.6	
75.8	45.3	0.002	0.7	1.673	Y55H10B.1	
20.4	12.2	0.007	0.7	1.672	Y50E8A.15	
26.4	15.8	0.003	0.7	1.671	F15A2.2	trehalase precursor
41.1	24.6	0	0.7	1.671	C29F9.11	

15.7	9.4	0.029	0.7	1.670	Y41D4B.26	nhr-146 - (Nuclear Hormone Receptor family)
42.9	25.7	0	0.7	1.669	R10E8.1	
26.2	15.7	0.004	0.7	1.669	ZK909.2B	cAMP-dependant protein kinase
22.5	13.5	0.02	0.7	1.667	C04A11.3	serine/threonine kinase
95.8	57.5	0.005	0.7	1.666	F11A6.1B	Subtilase family of serine proteases
184.6	110.8	0.008	0.7	1.666	Y105E8B.6	
130.5	78.4	0.001	0.7	1.665	C18C4.3	UDP-glucuronosyltransferase
23.8	14.3	0.003	0.7	1.664	F55A4.4	
384.9	231.3	0	0.7	1.664	Y57G11C.24B	epidermal growth factor receptor kinase substrate
95.3	57.3	0.022	0.7	1.663	C43E11.6	
472.4	284.3	0.001	0.7	1.662	T22H2.6A	granulin precursor
334.8	201.5	0.033	0.7	1.662	F23B2.2	
222.3	133.8	0.035	0.7	1.661	C05E11.5	ammonium transporter
21.1	12.7	0.032	0.7	1.661	F10B5.4	
1032	621.2	0.022	0.7	1.661	C49F5.1	s-adenosylmethionine synthetase
32.7	19.7	0	0.7	1.660	E01G6.3	serine esterase
86.4	52.1	0	0.7	1.658	F16H6.10	
170.1	102.6	0.013	0.7	1.658	C56363_rc	
132.9	80.2	0.001	0.7	1.657	B0041.6	6-pyruvoyl tetrahydrobioterin synthase
35.6	21.5	0.003	0.7	1.656	CEK071A3R_rc	
586.8	354.4	0.001	0.7	1.656	ZK1320.3	
57.1	34.5	0.003	0.7	1.655	Y39E4B.12	
35.9	21.7	0.029	0.7	1.654	Y105C5B.10	
483.7	292.4	0.021	0.7	1.654	T05C3.5	dnj-19 - (DNaJ domain (prokaryotic heat shock protein))
56.4	34.1	0.003	0.7	1.654	T22D1.11	carboxyesterase
109.3	66.1	0.02	0.7	1.654	Y47G6A.7	
49.1	29.7	0	0.7	1.653	Y116F11B.10	
27.6	16.7	0.006	0.7	1.653	K06C4.8	G-protein coupled receptor
116.5	70.5	0.019	0.7	1.652	B0281.3	
85.2	51.6	0.002	0.7	1.651	Y39G10AL.1	
13.7	8.3	0.045	0.7	1.651	Y25C1A.3	
34	20.6	0.007	0.7	1.650	B0280.2	
168.8	102.3	0.014	0.7	1.650	F22E5.1	
80.5	48.8	0.01	0.7	1.650	CTEL54X.1	
66.9	40.6	0.002	0.7	1.648	C26E1.2	
389.8	236.6	0.001	0.7	1.648	F56F10.1	peptidase
14	8.5	0.042	0.7	1.647	Y71H2AM.15	
631	383.2	0.025	0.7	1.647	F25B5.3	

732.9	445.5	0.001	0.7	1.645	R09H10.5	EGF domains
35.2	21.4	0	0.7	1.645	Y105C5B.27	
16.6	10.1	0.021	0.7	1.644	T12B5.2	
97.6	59.4	0.002	0.7	1.643	C05D2.8	
93.1	56.7	0.012	0.7	1.642	K03H1.10	
83.5	50.9	0.043	0.7	1.640	F44E8.1	reverse transcriptase
724.6	441.9	0.001	0.7	1.640	F54D5.4	
53.1	32.4	0.03	0.7	1.639	F45E4.1	ADP-ribosylation factor
191.4	116.8	0.001	0.7	1.639	T23G5.6	
17.2	10.5	0.018	0.7	1.638	Y26D4A.5	
16.7	10.2	0.022	0.7	1.637	C16C4.2	
319.1	194.9	0.001	0.7	1.637	Y43F8C.13	Inosine-uridine preferring nucleoside hydrolase
204.1	124.8	0.001	0.7	1.635	T26C12.3	
30.4	18.6	0.004	0.7	1.634	R13D11.7	
28.1	17.2	0.019	0.7	1.634	Y39E4B.9	
60.6	37.1	0.004	0.7	1.633	T05E7.1	
514.3	315	0.036	0.7	1.633	F10F2.2	Phosphoribosylformylglycinamidine synthase
27.9	17.1	0.032	0.7	1.632	C54C8.6	lys-9 - (LYSozyme)
30	18.4	0.034	0.7	1.630	K12G11.5	G-protein coupled receptor
126.5	77.6	0.002	0.7	1.630	Y56A3A.6	
408.8	251.1	0.023	0.7	1.628	R04E5.10	
29.3	18	0.001	0.7	1.628	ZK1010.8	
201.5	123.8	0.016	0.7	1.628	C43H6.1	
30.9	19	0.031	0.7	1.626	R11G10.2	selenium-binding protein like
473.5	291.3	0.005	0.7	1.625	ZK896.7	clec-186 - (C-type LECtin)
33.8	20.8	0.024	0.7	1.625	F52D2.7	
33.1	20.4	0.001	0.7	1.623	C17E7.9	
129.8	80	0.033	0.7	1.623	W03D8.6	
79.5	49	0.024	0.7	1.622	F22A3.4	human homeotic protein PBX2
15.9	9.8	0.03	0.7	1.622	K05F6.6	
38.9	24	0.002	0.7	1.621	T27F6.5	tRNA synthetases class II (Gly, His, Pro and Ser)
49.1	30.3	0.046	0.7	1.620	M163.1	
195.1	120.4	0.005	0.7	1.620	F55E10.6	D-beta-hydroxybutyrate dehydrogenase (BDH)
743.5	459	0.01	0.7	1.620	F54F11.2	Zinc-binding metalloprotease
90.7	56	0.031	0.7	1.620	C35A11.4	sugar transporter
684.5	422.8	0.001	0.7	1.619	R11H6.1	Yeast hypothetical 52.9 KD protein like
126.1	77.9	0.042	0.7	1.619	F41E6.3	
38.2	23.6	0	0.7	1.619	Y38E10A.21	

17.8	11	0.018	0.7	1.618	ZK1037.3	
69.2	42.8	0.008	0.7	1.617	C50H11.4	
19.4	12	0.042	0.7	1.617	Y105C5B.2	
52.2	32.3	0.004	0.7	1.616	K08A2.5	nuclear hormone receptor
40.4	25	0.02	0.7	1.616	F47H4.2	
48.8	30.2	0.016	0.7	1.616	Y119D3B.G	
26.5	16.4	0.044	0.7	1.616	Y47D3B.9	
376.6	233.1	0.001	0.7	1.616	K02D7.4	
34.4	21.3	0.011	0.7	1.615	W10C8.3	
26.8	16.6	0.002	0.7	1.614	F44C8.8	zinc finger protein
20.5	12.7	0.011	0.7	1.614	B0303.8	
16.3	10.1	0.028	0.7	1.614	F07G6.1	
26.3	16.3	0.003	0.7	1.613	ZK402.2	
248.8	154.4	0.001	0.7	1.611	C05D2.7	
557.8	346.2	0.007	0.7	1.611	C08B6.10	proline rich proteoglycan
60.9	37.8	0.005	0.7	1.611	F09G2.1	
68.9	42.8	0.001	0.7	1.610	C25F9.5	
138.4	86	0.018	0.7	1.609	Y14H12A.1	
118.6	73.7	0.015	0.7	1.609	F55F1.2	
25.9	16.1	0.006	0.7	1.609	Y39G8B.3	
56.3	35	0.038	0.7	1.609	ZC395.1	
26.2	16.3	0.016	0.7	1.607	F17H10.4	
14.3	8.9	0.049	0.7	1.607	T12A2.11	
19.6	12.2	0.032	0.7	1.607	Y8A9A.6	
19.9	12.4	0.014	0.7	1.605	B0547.2	
68.5	42.7	0.036	0.7	1.604	W02D7.3	
29.5	18.4	0.001	0.7	1.603	F47H4.8	
136.2	85	0.002	0.7	1.602	F59C6.3	
18.1	11.3	0.019	0.7	1.602	F27C8.3	
24.5	15.3	0.01	0.7	1.601	C10E2.1	
1067.9	667	0.012	0.7	1.601	B0286.3	saicar synthetase/air carboxlyase
32.5	20.3	0.047	0.7	1.601	Y113G7B.8	
14.4	9	0.048	0.7	1.600	C46F9.1	
72.8	45.5	0.009	0.7	1.600	F57G4.7	
89.9	56.2	0.021	0.7	1.600	T26H10.1	nicotinic acetylcholine receptor
966.4	604.6	0.004	0.7	1.598	R151.2	Ribose-phosphate pyrophosphokinase
39.8	24.9	0	0.7	1.598	C13C4.6	
57.5	36	0.028	0.7	1.597	F17C11.1	

290	181.6	0.001	0.7	1.597	F32D8.9	
41.2	25.8	0.001	0.7	1.597	C41G6.3	
1207.8	756.4	0.001	0.7	1.597	Y22F5A.4	lys-1 - (LYSozyme)
19.8	12.4	0.012	0.7	1.597	Y73E7A.C	
447.5	280.3	0.001	0.7	1.597	T22H2.6B	
185.5	116.2	0.003	0.7	1.596	C05D2.4	aromatic-L-amino-acid decarboxylase
78.2	49	0.017	0.7	1.596	C46H11.5	
718.7	450.6	0.01	0.7	1.595	ZK1307.1	
363.8	228.1	0.001	0.7	1.595	AU114285_rc	
623.5	391	0.002	0.7	1.595	Y43D4A.2	
171.9	107.8	0.001	0.7	1.595	R12A1.4	
33.8	21.2	0.029	0.7	1.594	B0019.3	
139.5	87.5	0.029	0.7	1.594	T18D3.3	cobalt uptake like protein
37.3	23.4	0.014	0.7	1.594	W04G5.6	tyrosine-protein kinase (KIN15/KIN16 subfamily) (4 domains)
24.7	15.5	0.008	0.7	1.594	T07D1.5	
32.5	20.4	0.001	0.7	1.593	Y69A2AR.4	smf-3 - (yeast SMF (divalent cation transporter) homolog)
30.1	18.9	0.05	0.7	1.593	C27A12.4	
58.6	36.8	0.029	0.7	1.592	ZC328.2	
356.8	224.2	0.006	0.7	1.591	F18E2.1	acid phophatase like
507.5	319.1	0.001	0.7	1.590	C26B9.5	lysosomal Pro-X carboxypeptidase
551.7	346.9	0.002	0.7	1.590	F01D5.5	
433	272.3	0.002	0.7	1.590	F32A5.3	Serine carboxypeptidase
15.1	9.5	0.046	0.7	1.589	W03D2.2	
150.8	94.9	0.005	0.7	1.589	F31D4.8	
96.1	60.6	0.026	0.7	1.586	C09H10.10	
22.2	14	0.046	0.7	1.586	C41G6.1	cytochrome P450
68.8	43.4	0.012	0.7	1.585	C49G7.10	
1672.7	1055.2	0.001	0.7	1.585	F35C5.6	clec-63 - Lectin C-type domain short and long forms, von Willebrand factor type A
29	18.3	0.005	0.7	1.585	C18C4.1	
233.1	147.1	0.001	0.7	1.585	Y41D4B.16	
383.3	242	0.001	0.7	1.584	R12C12.1	Glycine dehydrogenase
24.7	15.6	0.013	0.7	1.583	Y51A2A.10	
15.2	9.6	0.047	0.7	1.583	T23B3.3	
650.3	410.8	0.002	0.7	1.583	C01B10.6	
59.2	37.4	0.007	0.7	1.583	F58H7.1	
23.9	15.1	0.03	0.7	1.583	EGAP5.1	
60.6	38.3	0.017	0.7	1.582	F46B3.9	
764.9	483.7	0.002	0.7	1.581	Y105E8B.5	Hypoxanthine-guanine phosphoribosyltransferase

90.4	57.2	0.005	0.7	1.580	Y113G7A.13	
181.9	115.2	0.008	0.7	1.579	AV180727_rc	
59.2	37.5	0.016	0.7	1.579	Y71H2AM.2	
162.6	103	0.006	0.7	1.579	F55F3.2	
17.2	10.9	0.031	0.7	1.578	F54D7.1	reverse transcriptase
46.2	29.3	0.003	0.7	1.577	ZK418.7	
15.6	9.9	0.042	0.7	1.576	R07C3.8	
15.6	9.9	0.043	0.7	1.576	B0302.3	reverse transcriptase
42.7	27.1	0.002	0.7	1.576	T13H5.6	
71.2	45.2	0.046	0.7	1.575	C49A9.3	
29.6	18.8	0.002	0.7	1.574	F36A4.11	
993.7	631.4	0.005	0.7	1.574	Y45G12C.2	
848.6	539.3	0.012	0.7	1.574	Y69H2.3A	Fibrillins and related proteins containing Ca2+-binding EGF-like domains
324.1	206	0.034	0.7	1.573	F32A5.5	
562.1	357.3	0.003	0.7	1.573	C42D4.1	
25.8	16.4	0.013	0.7	1.573	F38B7.3	PH (pleckstrin homology) domain
184	117	0.002	0.7	1.573	F21D5.3	laccase like copper oxidase
42.3	26.9	0.004	0.7	1.572	Y47D7A.1	
44.5	28.3	0.005	0.7	1.572	C18H9.3	
68.4	43.5	0.01	0.7	1.572	F44F1.6B	
72.8	46.3	0.04	0.7	1.572	F10C2.3	
79.4	50.5	0.021	0.7	1.572	Y38H6A.2	
804.3	511.7	0.01	0.7	1.572	D2085.1	glutamine-dependent carbamoyl-phosphate synthase, aspartate carbamoyltransfe
23.1	14.7	0.013	0.7	1.571	F41H10.2	
2913.3	1855.7	0.016	0.7	1.570	T11F9.9	col-157 - (COLlagen)
26.2	16.7	0.015	0.7	1.569	H41C03.2	
19.6	12.5	0.016	0.7	1.568	F20B6.4	
175.6	112	0.007	0.7	1.568	T06C12.10	
98.1	62.6	0.008	0.7	1.567	K08C7.2	
115.8	73.9	0.006	0.7	1.567	F35C11.4	
18.8	12	0.041	0.6	1.567	F42H10.5	
175.6	112.1	0.009	0.7	1.566	F08A8.3	Acyl-CoA oxidase
58.1	37.1	0.008	0.7	1.566	C47F8.4	
45.1	28.8	0.003	0.7	1.566	T07H3.2	
28.5	18.2	0.023	0.7	1.566	Y39B6B.14	pro-3 (PROximal proliferation in germline)
459.9	293.7	0.023	0.7	1.566	Y38F2AL.3	vha-11 - (Vacuolar H ATPase)
104.9	67	0.019	0.7	1.566	T01C3.4	fil-1 - (Fasting Induced Lipase)
45.4	29	0.001	0.6	1.566	Y102A5B.3	Lectin C-type domain short and long forms

256.9	164.1	0.026	0.7	1.566	C39H7.4	
26.3	16.8	0.043	0.6	1.565	ZK909.4	albumin D-binding protein like
21.6	13.8	0.011	0.6	1.565	F47C12.5	chemoreceptor
1182.8	755.7	0.022	0.7	1.565	C49F5.1	s-adenosylmethionine synthetase
52.1	33.3	0	0.6	1.565	R04E5.2	
228.7	146.2	0.006	0.7	1.564	C54G4.1	Protein kinase C terminal domain
26.1	16.7	0.004	0.6	1.563	C35D6.5	
27.8	17.8	0.003	0.6	1.562	F49H6.4	
101.2	64.8	0.01	0.6	1.562	M02H5.1	
317.1	203.1	0.017	0.6	1.561	Y71H2AM.13	Carboxylesterase and related proteins
32	20.5	0.006	0.6	1.561	Y49F6B.1	
61.5	39.4	0.009	0.6	1.561	Y22D7AR.6	Inositol polyphosphate multikinase, component of the ARGR transcription regulato
16.7	10.7	0.05	0.6	1.561	K06A1.6	diacylglycerol kinase
51.5	33	0.002	0.6	1.561	ZK1240.1	
24.5	15.7	0.011	0.6	1.561	C07D8.2	
519.3	332.8	0.006	0.6	1.560	ZK484.2	transporter protein
55.2	35.4	0.02	0.6	1.559	T27B7.6	zinc finger protein
126.3	81	0.006	0.6	1.559	C40D2.2	math-20 - (MATH (meprin-associated Traf homology) domain containing)
241.3	154.8	0.003	0.6	1.559	F39G3.5	
24	15.4	0.018	0.6	1.558	Y111B2A.8	
78.2	50.2	0.004	0.6	1.558	ZK563.1	
33.9	21.8	0.015	0.6	1.555	Y97E10B.1	
16.3	10.5	0.04	0.6	1.552	R07C3.9	
46.4	29.9	0.001	0.6	1.552	Y87G2A.14	
112.5	72.5	0.032	0.6	1.552	C49A9.4	
33.2	21.4	0.016	0.6	1.551	T07H3.3	
367.3	236.9	0.047	0.6	1.550	F42C5.10	
42.3	27.3	0.008	0.6	1.549	Y67D8C.7	
76.8	49.6	0.014	0.6	1.548	T05C12.8	
191.1	123.5	0.001	0.6	1.547	F58H7.8	
16.4	10.6	0.04	0.6	1.547	Y94A7B.7	
884.5	571.7	0.014	0.6	1.547	C07B5.5	
41.3	26.7	0.025	0.6	1.547	C31H5.1	
419.5	271.4	0.014	0.6	1.546	F10G8.5	ncs-2 neuronal calcium sensor protein
68.3	44.2	0.019	0.6	1.545	Y23H5B.4	
53	34.3	0.007	0.6	1.545	F43C9.4	LDL-receptor class repeats; C1R/C1S-like repeats
26.1	16.9	0.005	0.6	1.544	ZK1037.4	Zinc finger, C4 type (two domains)
129.1	83.6	0.002	0.6	1.544	C44B7.11	

243.3	157.6	0.006	0.6	1.544	F55G1.11	Histone
77.9	50.5	0.019	0.6	1.543	Y116A8B.3	
89.3	57.9	0.015	0.6	1.542	F15H9.2	
23.9	15.5	0.044	0.6	1.542	Y53F4B.18	
1588.3	1030.5	0.012	0.6	1.541	C52E4.1	cathepsin-like cysteine protease
24.5	15.9	0.012	0.6	1.541	C02F12.4	guanine-nucleotide releasing factor of the CDC24 family
414.3	268.9	0.01	0.6	1.541	T27A1.5	
27.1	17.6	0.023	0.6	1.540	F55A3.5	glutathione peroxidase
52.5	34.1	0.008	0.6	1.540	H20E11.2	
278.2	180.7	0.003	0.6	1.540	T27A10.7	
95.9	62.3	0.002	0.6	1.539	F09F7.5	
21.7	14.1	0.025	0.6	1.539	T09E11.11	
51.4	33.4	0.002	0.6	1.539	K02B7.2	
439	285.4	0.017	0.6	1.538	ZK418.9	possible RNA binding protein
34.3	22.3	0.002	0.6	1.538	C58956_rc	
34.3	22.3	0.038	0.6	1.538	F54G8.1	
22.3	14.5	0.042	0.6	1.538	C46F9.3	
620.2	403.4	0.034	0.6	1.537	K04E7.2	Oligopeptide transporter
57.8	37.6	0.05	0.6	1.537	T12A2.15	
113.6	73.9	0.011	0.6	1.537	Y62H9A.3	
21.2	13.8	0.015	0.6	1.536	Y71H2B.8	
432.8	281.8	0.002	0.6	1.536	R102.4A	Yeast GLY1 like
26.7	17.4	0.009	0.6	1.534	C45H4.7	
23.3	15.2	0.009	0.6	1.533	R03D7.2	helicase domain
809.4	528.1	0.004	0.6	1.533	R07E3.1	cysteine proteinase
159	103.8	0.004	0.6	1.532	F49E11.9	scl-1 - (SCP-Like extracellular protein), predicted secretory protein that is a memb
17	11.1	0.038	0.6	1.532	C50B6.11	neurotransmitter-gated ion channel
22.5	14.7	0.025	0.6	1.531	Y48C3A.11	
1344.6	878.9	0.001	0.6	1.530	K10C2.3	aspartyl protease
71.9	47	0.032	0.6	1.530	C24A11.2	
31.5	20.6	0.036	0.6	1.529	F20C5.2	klp-11 - (Kinesin-Like Protein)
47.4	31	0.005	0.6	1.529	K08E7.7	cul-6 - (CULlin)
42.8	28	0.005	0.6	1.529	F55A4.5	
24.9	16.3	0.008	0.6	1.528	R12E2.5	
24.9	16.3	0.049	0.6	1.528	W02H5.3	
264.8	173.4	0.007	0.6	1.527	F55A12.9	
20	13.1	0.036	0.6	1.527	F55G1.15	
22.9	15	0.012	0.6	1.527	F49H12.4	

337.8	221.4	0.007	0.6	1.526	F56F3.2	
138.7	91	0.015	0.6	1.524	Y38F2AR.10	
142.3	93.4	0.007	0.6	1.524	Y38E10A.7	
498.1	327.1	0.007	0.6	1.523	LLC1.3	dihydrolipoamide dehydrogenase
1217.8	800.2	0.045	0.6	1.522	F53F1.5	cuticlin
485.2	319	0.007	0.6	1.521	R09H10.3	transthyretin like
26	17.1	0.034	0.6	1.520	Y51H4A.20	
19	12.5	0.027	0.6	1.520	F37A8.5	
57.6	37.9	0.011	0.6	1.520	ZC239.16	
74.1	48.8	0.012	0.6	1.518	Y22D7AL.4	
25.8	17	0.007	0.6	1.518	C53A5.11	ring canal protein like
35.2	23.2	0.028	0.6	1.517	Y50D4C.3	
103.1	68	0.002	0.6	1.516	Y23H5B.3	
43.2	28.5	0.03	0.6	1.516	F28F5.3	
930	613.6	0.002	0.6	1.516	M04G12.2	cysteine protease
29.1	19.2	0.007	0.6	1.516	ZC204.11	
53.5	35.3	0	0.6	1.516	F54H5.5	
31.2	20.6	0.003	0.6	1.515	F46C3.2	
47.7	31.5	0.001	0.6	1.514	Y73B3A.11	
534.5	353.1	0.016	0.6	1.514	K11E8.1	unc-43 - (type II calcium/calmodulin-dependent protein kinase (CaMKII) ortholog)
28.3	18.7	0.005	0.6	1.513	F40G9.1	
17.7	11.7	0.039	0.6	1.513	Y9C9A.15	
402.5	266.3	0.015	0.6	1.511	C07G2.2A	atf-7 - (ATF (cAMP-dependent transcription factor) family)
32	21.2	0.037	0.6	1.509	Y24D9A.5	
65.5	43.4	0.019	0.6	1.509	R04D3.3	
115	76.2	0.009	0.6	1.509	K08C7.2	
16.9	11.2	0.047	0.6	1.509	W10G11.15	
17.2	11.4	0.042	0.6	1.509	Y54E10A.13	
30	19.9	0.01	0.6	1.508	E01G4.5	
31.5	20.9	0.041	0.6	1.507	F46F2.2	kin-20 - (protein KINase)
63.9	42.4	0.003	0.6	1.507	M04C3.2	
110.6	73.4	0.007	0.6	1.507	C42D8.1	
2683.4	1781	0.002	0.6	1.507	T25F10.6	calponin-like protein
34.2	22.7	0.028	0.6	1.507	C01B4.8	
161.5	107.2	0.019	0.6	1.507	F58A3.3	
57.7	38.3	0.015	0.6	1.507	Y5H2B.6	
25.6	17	0.009	0.6	1.506	F45E1.5	
27.7	18.4	0.023	0.6	1.505	T03D3.2	srj-50 - (Serpentine Receptor, class J)

47.1	31.3	0.002	0.6	1.505	F59E12.8	
31.6	21	0.004	0.6	1.505	F31F6.3	member of worm-specific protein family
48.3	32.1	0.016	0.6	1.505	T26H2.9	
98.1	65.2	0.012	0.6	1.505	Y48G1BR.A	
91	60.5	0.029	0.6	1.504	Y51H1A.1	
22.1	14.7	0.049	0.6	1.503	C28H8.8	
154.7	102.9	0.01	0.6	1.503	R05D8.8	alcohol dehydrogenase
23.3	15.5	0.02	0.6	1.503	T05F1.7	
52	34.6	0.03	0.6	1.503	ZK1307.7	
27.8	18.5	0.006	0.6	1.503	F28C6.1	transcription factor AP-2 like
626.6	417.1	0.002	0.6	1.502	F22E12.1	BPTI/KUNITZ inhibitor domain
34.7	23.1	0.038	0.6	1.502	F20D1.5	ADP-ribosylation factor
201.9	134.5	0.006	0.6	1.501	Y54F10AL.1	Predicted membrane protein
27	18	0.033	0.6	1.500	Y67H2B.B	
841.2	561	0.036	0.6	1.499	F45H10.3	NADH:ubiquinone oxidoreductase, NDUFA7/B14.5A subunit
80.8	53.9	0.005	0.6	1.499	Y69A2AR.D	
91.7	61.2	0.005	0.6	1.498	F20B4.6	
261.9	174.8	0.016	0.6	1.498	C47A4.2	
31.3	20.9	0.049	0.6	1.498	M01D7.3	
301.6	201.4	0.036	0.6	1.498	F42F12.4	
153	102.2	0.01	0.6	1.497	W05E10.4	trehalase
20.5	13.7	0.024	0.6	1.496	Y97E10AR.1	
77.5	51.8	0.012	0.6	1.496	Y57A10B.6	
561.9	375.7	0.007	0.6	1.496	Y71H2AM.L	
67	44.8	0.002	0.6	1.496	F13H8.1	
132.8	88.8	0.002	0.6	1.495	C46H11.4	
196.5	131.4	0.014	0.6	1.495	F10G7.5	
28.7	19.2	0.006	0.6	1.495	Y43F8A.3	
358.3	239.7	0.039	0.6	1.495	CEK034B7R_rc	
55	36.8	0.005	0.6	1.495	F27E5.5	
27.5	18.4	0.007	0.6	1.495	ZK402.3	
51.7	34.6	0.005	0.6	1.494	Y41C4A.3	
229.5	153.7	0.027	0.6	1.493	F10E7.2	
53.9	36.1	0.001	0.6	1.493	F44C8.11	nuclear hormone receptor
32.1	21.5	0.012	0.6	1.493	F07G11.7	
119.7	80.2	0.013	0.6	1.493	C01G6.8	protein tyrosine kinase receptor
99.7	66.8	0.019	0.6	1.493	Y55B1BR.1	
258.8	173.4	0.042	0.6	1.493	C09H10.8	

29.4	19.7	0.041	0.6	1.492	C02E7.14	reverse transcriptase
28.5	19.1	0.007	0.6	1.492	C50D2.8	
47.3	31.7	0.002	0.6	1.492	Y45F10D.10	
877	588	0.004	0.6	1.491	VW02B12L.1	vacuolar ATP synthase
40.7	27.3	0.003	0.6	1.491	W06F12.2B	
154.1	103.4	0.032	0.6	1.490	T15B7.9	
22.8	15.3	0.016	0.6	1.490	C27C12.3	member of worm-specific protein family
102.8	69	0.04	0.6	1.490	W03D8.7	
226.6	152.1	0.002	0.6	1.490	C57244	
51.1	34.3	0.015	0.6	1.490	Y57G7A.1	
48.7	32.7	0.002	0.6	1.489	K08B4.3	glucuronosyltransferase
416.2	279.5	0.005	0.6	1.489	R05A10.7	
20.1	13.5	0.028	0.6	1.489	D1065.5	chemoreceptor
440.4	295.9	0.002	0.6	1.488	F49D11.8	
31.7	21.3	0.004	0.6	1.488	F14F8.1	
37.8	25.4	0.006	0.6	1.488	H03G16.3	
57.7	38.8	0.043	0.6	1.487	Y39B6B.BB	
193.6	130.2	0.007	0.6	1.487	F55B11.1	xanthine dehydrogenase
882.2	593.6	0.003	0.6	1.486	R107.7	Glutathione S-transferase P subunit
407.3	274.2	0.017	0.6	1.485	C05D2.7	
40.4	27.2	0.047	0.6	1.485	Y53F4B.20	
50.9	34.3	0.006	0.6	1.484	C30G4.4	
41.4	27.9	0.009	0.6	1.484	T06C12.3	7TM receptor
307.6	207.3	0.02	0.6	1.484	B0285.9	choline kinase
66.9	45.1	0.01	0.6	1.483	ZK1055.6	
66	44.5	0.031	0.6	1.483	T10B5.4	
405.6	273.7	0.002	0.6	1.482	C08B11.4	
400.8	270.6	0.002	0.6	1.481	Y48B6A.12	
23.1	15.6	0.018	0.6	1.481	C52B9.6	
19.1	12.9	0.035	0.6	1.481	Y71F9AL.3	transposon
26.2	17.7	0.019	0.6	1.480	C29F3.2	wrt-8 - (WaRThog (hedgehog-like family))
26.2	17.7	0.019	0.6	1.480	T13F2.8	cav-1 - (CAVeolin)
95.6	64.6	0.004	0.6	1.480	T21C9.6	pyruvate, water dikinase
32.7	22.1	0.004	0.6	1.480	F35D11.11	
713.6	482.4	0.018	0.6	1.479	C34624_rc	
1298.2	877.6	0.006	0.6	1.479	W01A11.4	lec-10 - (gaLECtin)
56.8	38.4	0.042	0.6	1.479	K10C9.1	
27.5	18.6	0.009	0.6	1.478	T08G5.7	Zinc finger, C2H2 type (3 domains)

289.9	196.2	0.009	0.6	1.478	W02D3.2	dihydroorotate dehydrogenase
42.7	28.9	0.025	0.6	1.478	R12C12.4	
74.9	50.7	0.003	0.6	1.477	C03H12.1	
19.2	13	0.037	0.6	1.477	Y51H4A.1	
150.6	102	0.04	0.6	1.476	Y46G5A.21	
31.3	21.2	0.005	0.6	1.476	Y38C1BA.2	
27	18.3	0.015	0.6	1.475	C01B7.2	
59.6	40.4	0.036	0.6	1.475	M03E7.1	
26.7	18.1	0.01	0.6	1.475	C23H4.3	
186.6	126.5	0.012	0.6	1.475	C41D11.6	
23.6	16	0.033	0.6	1.475	K10B4.1	peroxidase
268.5	182.1	0.021	0.6	1.474	F58G6.2	
40.1	27.2	0.004	0.6	1.474	T05B4.11	
1370.6	929.8	0.023	0.6	1.474	F01F1.12	Fructose-biphosphate aldolase
1052.4	714.2	0.008	0.6	1.474	F09B12.3	
626.7	425.4	0.009	0.6	1.473	F46G10.1	
1022.8	694.4	0.023	0.6	1.473	F10C1.7B	Intermediate filament protein
27.1	18.4	0.011	0.6	1.473	Y48G1BL.B	
77.6	52.7	0.007	0.6	1.472	g5734141	
37.1	25.2	0.003	0.6	1.472	M153.3	
101.7	69.1	0.046	0.6	1.472	F56D2.5	
246.5	167.5	0.006	0.6	1.472	T02E1.4	dehydrogenase
777.4	528.3	0.003	0.6	1.472	F44F4.11	tubulin alpha-2 chain
736.3	500.6	0.004	0.6	1.471	T26C5.1	glutathione S-transferase
71.6	48.7	0.024	0.6	1.470	M01F1.7	Phosphatidylinositol transfer protein
73.2	49.8	0.003	0.6	1.470	T19D12.2	
21.9	14.9	0.035	0.6	1.470	F07H5.12	
171.8	116.9	0.012	0.6	1.470	Y39B6A.41	Uncharacterized protein
36	24.5	0.009	0.6	1.469	Y104H12D.3	
21.3	14.5	0.036	0.6	1.469	K10G6.1	
113.4	77.2	0.043	0.6	1.469	C45G3.2B	
27.9	19	0.012	0.6	1.468	ZK39.8	clec-99 - (C-type LECtin)
27.9	19	0.016	0.6	1.468	C14B4.2	
371.2	252.8	0.003	0.6	1.468	g5805381	
75.6	51.5	0.015	0.6	1.468	W07G4.6	
70	47.7	0.038	0.6	1.468	Y53G8B.1	
290.1	197.7	0.017	0.6	1.467	ZK856.10	DNA directed RNA polymerase III
96.1	65.5	0.017	0.6	1.467	Y71H2B.7	

423.1	288.4	0.023	0.6	1.467	D2096.3	
19.5	13.3	0.038	0.6	1.466	R105.2	
1412.1	963.2	0.003	0.6	1.466	M60.1	
808.5	551.6	0.048	0.6	1.466	C55F2.1	
714.5	487.6	0.003	0.6	1.465	K05C4.1	Proteasome A-type and B-type
1538.5	1050.2	0.003	0.6	1.465	W02D3.5	fatty acid-binding protein
24.9	17	0.015	0.6	1.465	F39D8.2	
70.3	48	0.042	0.6	1.465	F13E9.3	fipr-15
18.6	12.7	0.045	0.6	1.465	C55A1.10	
150.7	102.9	0.028	0.6	1.465	F35E12.8	
211.9	144.7	0.003	0.6	1.464	Y66H1A.6	
96.5	65.9	0.018	0.6	1.464	T12B5.8	
30	20.5	0.018	0.6	1.463	W09D12.2	
18	12.3	0.05	0.6	1.463	AV184387_rc	
450.7	308	0.014	0.6	1.463	F54E2.1	
138.7	94.8	0.005	0.6	1.463	T19D2.2	protein-tyrosine phosphotase
29.7	20.3	0.007	0.6	1.463	F43C11.7	
31.6	21.6	0.038	0.6	1.463	T11F1.6	
19.3	13.2	0.039	0.6	1.462	F17B5.4	
717.9	491	0.048	0.6	1.462	C16B8.3	
41.8	28.6	0.011	0.6	1.462	F45C12.4	
139.7	95.6	0.03	0.6	1.461	Y45F3A.5	
22.5	15.4	0.031	0.6	1.461	W10G11.3	
150.7	103.2	0.04	0.6	1.460	F40F12.7	
1664.2	1140	0.038	0.6	1.460	CEK107E8F	
34.3	23.5	0.005	0.6	1.460	C55A1.2	
25.1	17.2	0.015	0.6	1.459	Y23H5B.B	
558.8	383	0.006	0.6	1.459	F42D1.2	tyrosine aminotransferase
54.7	37.5	0.019	0.5	1.459	F31F7.3	
131.8	90.4	0.03	0.5	1.458	D2023.7	collagen
1304.1	894.5	0.004	0.5	1.458	Y55B1AR.1	
44.9	30.8	0.005	0.6	1.458	T27B7.4	zinc finger protein
1246.8	855.3	0.012	0.5	1.458	C55B7.4	acyl-CoA dehydrogenase
42.7	29.3	0.01	0.5	1.457	C17E7.6	nuclear hormone receptor
171.5	117.7	0.003	0.5	1.457	C14A4.6	
254.6	174.8	0.047	0.5	1.457	F49E2.2	
28.4	19.5	0.01	0.5	1.456	F13E9.10	
58.1	39.9	0.033	0.5	1.456	Y38F2AR.B	

46.3	31.8	0.004	0.5	1.456	F57C7.2	
795.8	546.6	0.012	0.5	1.456	F52E1.5	
171.5	117.8	0.039	0.5	1.456	F15H9.1	
34.5	23.7	0.005	0.5	1.456	T25E12.4B	
420.5	288.9	0.037	0.5	1.456	C53B4.7	
62	42.6	0.004	0.5	1.455	R05A10.4	
138.4	95.1	0.004	0.5	1.455	T28F12.2	unc-62 homeobox protein
84.1	57.8	0.01	0.5	1.455	F47C12.1	
37.1	25.5	0.012	0.5	1.455	M163.6	
45.8	31.5	0.013	0.5	1.454	F08A8.5	
39.1	26.9	0.003	0.5	1.454	M195.4	
27.9	19.2	0.018	0.5	1.453	F09B12.5	Low-density lipoprotein receptor domain class A (2 domains)
511.6	352.1	0.004	0.5	1.453	Y54G2A.X	
23.1	15.9	0.023	0.5	1.453	R03G5.6	
21.5	14.8	0.041	0.5	1.453	F47B7.5	
53.6	36.9	0.017	0.5	1.453	K10D11.6	
38.2	26.3	0.049	0.5	1.452	F58G11.2	ATP-dependent RNA helicase like
216.2	148.9	0.004	0.5	1.452	T02C12.1	myosin IA
27	18.6	0.038	0.5	1.452	K07F5.11_rc	
25.4	17.5	0.016	0.5	1.451	K09E2.1	
718.1	494.8	0.005	0.5	1.451	F23B2.1	
206	142	0.006	0.5	1.451	C33H5.13	
124.3	85.7	0.006	0.5	1.450	F33E2.2	Eukaryotic protein kinase domain
40.9	28.2	0.025	0.5	1.450	T10B5.1	transposase
226.1	155.9	0.018	0.5	1.450	Y37D8A.6	
77	53.1	0.005	0.5	1.450	C46C11.2	
80.9	55.8	0.027	0.5	1.450	C38C3.8	
1364.8	941.4	0.004	0.5	1.450	ZK1290.12	serine carboxypeptidase
25.8	17.8	0.02	0.5	1.449	Y22D7AL.F	
31.3	21.6	0.008	0.5	1.449	K09F6.5	
49.7	34.3	0.009	0.5	1.449	M199.4	
21.3	14.7	0.032	0.5	1.449	C46E1.1	
1720	1187.1	0.006	0.5	1.449	F58G1.4	
72.4	50	0.036	0.5	1.448	Y119C1B.H	
68.2	47.1	0.028	0.5	1.448	F43C9.1	
598.7	413.5	0.013	0.5	1.448	C34F11.3	AMP deaminase
20.7	14.3	0.035	0.5	1.448	W06A11.3	
55	38	0.004	0.5	1.447	M01G12.10	

1241.6	858.1	0.008	0.5	1.447	AU115274_rc	
3477.8	2403.8	0.012	0.5	1.447	T28C6.6	col-3 - (COLlagen)
351.5	243.1	0.031	0.5	1.446	C24G6.5	
85.3	59	0.032	0.5	1.446	H19M22.2	
35.7	24.7	0.005	0.5	1.445	M01G4.1	
1689.7	1169.1	0.019	0.5	1.445	CEK001CYR_rc	
78.6	54.4	0.004	0.5	1.445	C38H2.2	
544.2	376.7	0.004	0.5	1.445	ZK112.1	
40.3	27.9	0.039	0.5	1.444	F45C12.8	
167.8	116.2	0.007	0.5	1.444	F42A10.3	
28.3	19.6	0.019	0.5	1.444	B0491.8	chloride channel protein
27	18.7	0.014	0.5	1.444	Y18D10A.10	
243.4	168.6	0.039	0.5	1.444	F08G5.2	
461.2	319.5	0.008	0.5	1.444	W02D3.7	fatty acid-binding protein
326.8	226.4	0.03	0.5	1.443	T08B1.1	sugar transporter
697.2	483.1	0.044	0.5	1.443	Y42H9B.C	
1109.3	768.7	0.019	0.5	1.443	C15H9.6	heat shock protein
22.8	15.8	0.027	0.5	1.443	C31B8.1	
68.1	47.2	0.025	0.5	1.443	F12E12.I	
137.3	95.2	0.017	0.5	1.442	F58F9.4	
26.1	18.1	0.02	0.5	1.442	F30H5.1	P59 protein and TPR domain
23.5	16.3	0.05	0.5	1.442	ZK856.6	
165.9	115.1	0.012	0.5	1.441	W04E12.6	Lectin C-type domain short and long forms (2 domains)
84.3	58.5	0.045	0.5	1.441	Y105C5B.9	
23.2	16.1	0.025	0.5	1.441	Y55B1BR.3	
118.1	82	0.004	0.5	1.440	C57525_rc	
200.3	139.1	0.022	0.5	1.440	R01B10.3	
81.5	56.6	0.016	0.5	1.440	B0294.2	
148.6	103.2	0.036	0.5	1.440	E03G2.2	multidrug resistance-associated protein
128.4	89.2	0.01	0.5	1.439	ZK896.6	lectin (C-type)
65.9	45.8	0.047	0.5	1.439	Y71H2AM.F	
149.6	104	0.012	0.5	1.438	R09F10.1	
1052.3	731.6	0.008	0.5	1.438	F28H7.3	lipase
169	117.5	0.034	0.5	1.438	K02D7.3	cuticular collagen
1379.5	959.3	0.009	0.5	1.438	D1037.3	ferritin
19.7	13.7	0.044	0.5	1.438	T23B12.10	
96.7	67.3	0.006	0.5	1.437	F25F8.1	
624.1	434.4	0.004	0.5	1.437	C49C3.4	EGF-like domain

45.8	31.9	0.015	0.5	1.436	T23D8.5	histone H4
81.2	56.6	0.02	0.5	1.435	C44C8.6	protein kinase
161.7	112.8	0.017	0.5	1.434	H19N07.4	
177	123.5	0.012	0.5	1.433	C18A11.3	
19.2	13.4	0.05	0.5	1.433	ZK770.1	asic-1 - (Acid-sensing/Amiloride-Sensitive Ion Channel family)
24.5	17.1	0.028	0.5	1.433	C34F6.6	
368.2	257.1	0.005	0.5	1.432	F22E12.1	BPTI/KUNITZ inhibitor domain
100.8	70.4	0.018	0.5	1.432	F42G2.6	
38.5	26.9	0.014	0.5	1.431	R11.3	
24.9	17.4	0.034	0.5	1.431	C09G1.2	
341.1	238.5	0.024	0.5	1.430	ZK1320.1	
86.2	60.3	0.025	0.5	1.430	K03H6.4	
29.3	20.5	0.011	0.5	1.429	C05C12.6	
107.9	75.5	0.036	0.5	1.429	F19B10.1	
292.2	204.5	0.005	0.5	1.429	F32A7.5	claustrin like, microtubule-associated protein
1912.9	1338.8	0.047	0.5	1.429	C53A5.1	
192.5	134.8	0.006	0.5	1.428	M01A10.5	
36.7	25.7	0.017	0.5	1.428	ZK218.7	
33.7	23.6	0.011	0.5	1.428	F55E10.7	
33.7	23.6	0.02	0.5	1.428	C29F9.6	
309	216.4	0.01	0.5	1.428	F08F8.4	
413.5	289.6	0.026	0.5	1.428	T05H4.6	eukaryotic peptide chain release factor subunit 1
30.4	21.3	0.032	0.5	1.427	Y26G10.1	
816.8	572.5	0.005	0.5	1.427	C29E4.1	Cuticle collagen
1054.7	739.4	0.005	0.5	1.426	F19C7.1	
147.2	103.2	0.022	0.5	1.426	H06H21.8	
372.3	261.2	0.007	0.5	1.425	Y69F12A.2	aldehyde dehydrogenase
96.6	67.8	0.012	0.5	1.425	C24A1.3	
24.5	17.2	0.024	0.5	1.424	E02H4.5	
24.5	17.2	0.027	0.5	1.424	C12D12.6	
24.5	17.2	0.039	0.5	1.424	F49D11.5	reverse transcriptase
29.2	20.5	0.014	0.5	1.424	F53B6.6	cuticulin
411.4	288.9	0.016	0.5	1.424	F44G3.6	
705.9	495.8	0.01	0.5	1.424	AV198952_rc	
216.2	151.9	0.009	0.5	1.423	W10G6.2	Protein kinase C terminal domain
263.2	185	0.032	0.5	1.423	C04F12.1	
75.4	53	0.008	0.5	1.423	C03G6.12	C4-type zinc finger protein
213.8	150.3	0.005	0.5	1.422	C14C11.3	

45.8	32.2	0.004	0.5	1.422	W02H5.1	
29.3	20.6	0.048	0.5	1.422	C35A11.3	
187	131.5	0.012	0.5	1.422	F09A5.4A	
712.2	500.9	0.011	0.5	1.422	C18H9.6	
90.4	63.6	0.034	0.5	1.421	Y54E2A.6	Helicases conserved C-terminal domain
25	17.6	0.024	0.5	1.420	T27C5.7	
719.5	506.7	0.018	0.5	1.420	Y16B4A.2	serine carboxypeptidase
132.9	93.6	0.026	0.5	1.420	Y113G7B.2	
31.8	22.4	0.034	0.5	1.420	T23F1.5	
138.1	97.3	0.006	0.5	1.419	F09C3.1	ras GTPase-activating protein like
2606	1836.1	0.022	0.5	1.419	F30B5.1	dpy-13- collagen
133.4	94	0.011	0.5	1.419	T19H5.4	
24.4	17.2	0.026	0.5	1.419	R52.3	
722	509.1	0.005	0.5	1.418	Y24D9A.F	
3081.9	2173.2	0.026	0.5	1.418	T07H6.3	col-166 - (COLlagen)
217.4	153.3	0.037	0.5	1.418	F45H10.4	
82.1	57.9	0.005	0.5	1.418	T12A2.14	
53	37.4	0.002	0.5	1.417	F55F8.8	
206.3	145.6	0.015	0.5	1.417	C52D10.1	
2108.9	1488.5	0.01	0.5	1.417	B0222.7	
748	528	0.006	0.5	1.417	C01G8.5B	membrane protein
86.6	61.2	0.04	0.5	1.415	F56G4.1	
68.2	48.2	0.016	0.5	1.415	C38C3.2	
467.3	330.4	0.005	0.5	1.414	Y54G11A.5B	ctl-2 - (CaTaLase)
83.4	59	0.026	0.5	1.414	F23F12.1	
77.6	54.9	0.005	0.5	1.413	F02D10.5	sodium channel like
1656.2	1171.8	0.005	0.5	1.413	T25C8.2	Actins
28.4	20.1	0.016	0.5	1.413	Y47D3A.23	
211.2	149.6	0.027	0.5	1.412	Y22D7AL.B	
39.8	28.2	0.031	0.5	1.411	M03F8.4	
439.3	311.4	0.027	0.5	1.411	Y50E8A.1	
221.8	157.3	0.006	0.5	1.410	CEK112E8R_rc	
45.4	32.2	0.039	0.5	1.410	C30F2.1	collagen
46.8	33.2	0.006	0.5	1.410	C09E10.2B	diacylglycerol kinase
44.4	31.5	0.036	0.5	1.410	K07G5.5	
126.7	89.9	0.006	0.5	1.409	g8979463	
1130.6	802.4	0.006	0.5	1.409	K12H4.7	
34.8	24.7	0.009	0.5	1.409	Y76B12C.5	

1012.6	719	0.006	0.5	1.408	W04E12.8	clec-50 - (C-type LECtin)
1839.4	1306.1	0.006	0.5	1.408	H06O01.1	protein disulphide isomerase ER-60 precursor like
1101.2	782.3	0.006	0.5	1.408	C35B1.5	
29.7	21.1	0.016	0.5	1.408	K07A9.2	ser/thr protein kinase
574.1	407.9	0.039	0.5	1.407	F28A12.4	peptidase
1080.9	768	0.007	0.5	1.407	Y38F1A.6	Iron-containing alcohol dehydrogenases
23.5	16.7	0.035	0.5	1.407	T28H11.3	chemoreceptor
791.1	562.2	0.007	0.5	1.407	C18B2.5	
32.5	23.1	0.047	0.5	1.407	T09D3.1	chemoreceptor
24.9	17.7	0.028	0.5	1.407	F34D6.5	chemoreceptor
54.3	38.6	0.028	0.5	1.407	ZK678.1	lin-15A - (abnormal cell LINeage)
104.2	74.1	0.006	0.5	1.406	T25G12.7	dehydrogenase
47.8	34	0.042	0.5	1.406	F40H6.4	
137.2	97.6	0.006	0.5	1.406	E03D2.2	
36.4	25.9	0.013	0.5	1.405	Y27F2A.7	chemoreceptor
121.8	86.7	0.016	0.5	1.405	F45E1.7	
23.6	16.8	0.035	0.5	1.405	Y37H9A.2	
30.9	22	0.014	0.5	1.405	Y71G10AL.A	
32.3	23	0.012	0.5	1.404	W03B1.2	
29.2	20.8	0.017	0.5	1.404	ZK616.A	
44.5	31.7	0.013	0.5	1.404	F43C11.9	
599	426.9	0.006	0.5	1.403	T02G5.7	acetoacetyl CoA thiolase
36.9	26.3	0.038	0.5	1.403	C18D11.3	
67.2	47.9	0.049	0.5	1.403	EEED8.14	
31.7	22.6	0.013	0.5	1.403	Y116A8C.10	
34.5	24.6	0.034	0.5	1.402	W08F4.9	
871.5	621.5	0.01	0.5	1.402	C23H5.8B	
34.6	24.7	0.03	0.5	1.401	F42G9.9A	
254.5	181.7	0.021	0.5	1.401	ZK945.1	esterase
45.1	32.2	0.005	0.5	1.401	ZC84.5	
780.1	557	0.03	0.5	1.401	T02C5.1	
75.2	53.7	0.04	0.5	1.400	T22F7.1	
90.6	64.7	0.043	0.5	1.400	C35B8.2	vav-1 - (mammalian VAV (oncogene) related)
319.1	227.9	0.014	0.5	1.400	Y106G6H.1	
2333	1666.4	0.012	0.5	1.400	T08B2.10	40S ribosomal protein S17
2186.1	1562.7	0.033	0.5	1.399	F41F3.3	cuticlin
355.1	253.9	0.006	0.5	1.399	CEK133E3R_rc	
57.2	40.9	0.047	0.5	1.399	C14E2.3	

37.9	27.1	0.025	0.5	1.399	T06G6.4	
707.8	506.2	0.007	0.5	1.398	F01F1.9	
26	18.6	0.041	0.5	1.398	F33H1.5	
387.6	277.5	0.007	0.5	1.397	C02E11.1	
108.8	77.9	0.01	0.5	1.397	C07A12.3	nhr-35 - (Nuclear Hormone Receptor family)
104.6	74.9	0.017	0.5	1.397	C53C11.1	
24.7	17.7	0.03	0.5	1.395	T06G6.5	
230.8	165.4	0.014	0.5	1.395	ZK525.2	
141.9	101.7	0.016	0.5	1.395	C16D9.4	
1354.6	971.1	0.007	0.5	1.395	Y57G11C.15	protein transport protein SEC61 alpha subunit
79.5	57	0.034	0.5	1.395	T10G3.2	
1128.2	809	0.047	0.5	1.395	VC5.3	
592.8	425.1	0.008	0.5	1.394	Y53F4B.29	glutathione-S-transferase
25.1	18	0.047	0.5	1.394	ZK993.1	homeobox protein
208	149.2	0.028	0.5	1.394	K02A11.1	gfi-2 - (GEI-4 (Four) Interacting protein), myosin phosphatase
2590.7	1858.8	0.013	0.5	1.394	W05B2.5	
866	621.4	0.014	0.5	1.394	C07D8.6	aldehyde reductase
277.7	199.3	0.027	0.5	1.393	F54F3.1	nid-1 - (NIDogen (basement membrane protein)
32.6	23.4	0.032	0.5	1.393	F36D1.5	
40.4	29	0.037	0.5	1.393	Y95B8A.10	phosphodiesterase
1455.8	1045.6	0.047	0.5	1.392	C53B4.5	collagen
1455.8	1045.6	0.047	0.5	1.392	C53B4.5	collagen
49.7	35.7	0.005	0.5	1.392	F14B8.1	NA(+)/H(+) antiporter
56.8	40.8	0.03	0.5	1.392	F59D6.2	aspartyl protease
59.3	42.6	0.03	0.5	1.392	M04D8.3	ins-23
87.4	62.8	0.03	0.5	1.392	C17H11.6	
71.1	51.1	0.014	0.5	1.391	Y116A8C.6	
108.1	77.7	0.012	0.5	1.391	F36H1.2	ankyrin like
91.4	65.7	0.016	0.5	1.391	Y116A8C.29	
248.6	178.7	0.008	0.5	1.391	C18E9.9	
42.7	30.7	0.006	0.5	1.391	C37A2.2	
57.7	41.5	0.02	0.5	1.390	F19H6.6	
33.5	24.1	0.021	0.5	1.390	C50H2.4	
41.7	30	0.007	0.5	1.390	Y111B2A.23	
199.6	143.6	0.038	0.5	1.390	Y67D8C.C	
912.4	656.8	0.01	0.5	1.389	C05D11.11	
94.6	68.1	0.015	0.5	1.389	Y55B1BM.1	
148.6	107	0.016	0.5	1.389	Y55H10B.2	

38.3	27.6	0.036	0.5	1.388	F47C10.6	
53	38.2	0.006	0.5	1.387	F13B9.6	
46.2	33.3	0.046	0.5	1.387	C08F11.5	
594.2	428.4	0.007	0.5	1.387	Y71F9AL.9	
23.3	16.8	0.043	0.5	1.387	ZK39.3	Lectin C-type domain
171	123.3	0.019	0.5	1.387	F14B8.6	
59.6	43	0.023	0.5	1.386	R08E3.2	similar to human tenascin-X
300	216.5	0.012	0.5	1.386	C28C12.5	
145.2	104.8	0.045	0.5	1.385	Y43H11AL.D	
189.2	136.6	0.024	0.5	1.385	AV199244_rc	
152.2	109.9	0.046	0.5	1.385	H10E21.1	
167.7	121.1	0.011	0.5	1.385	R173.1	
51.9	37.5	0.015	0.5	1.384	M02F4.9	
158.7	114.7	0.027	0.5	1.384	F21F8.6	protease
1031.2	745.3	0.026	0.5	1.384	F32D1.5	GMP reductase
114	82.4	0.039	0.5	1.383	C30G7.1	histone H1 like
41.9	30.3	0.008	0.5	1.383	W10G11.12	
1193.3	863.1	0.009	0.5	1.383	F20H11.3	malate dehydrogenase
120.4	87.1	0.023	0.5	1.382	C34B2.6	protease
53.6	38.8	0.004	0.5	1.381	C38D9.5	
23.2	16.8	0.044	0.5	1.381	Y41D4A.A	
327.8	237.4	0.01	0.5	1.381	C47D2.2	cytidine deaminase
43.2	31.3	0.034	0.5	1.380	T13F3.1	7TM receptor
54.1	39.2	0.041	0.5	1.380	H22K11.2	
330.8	239.7	0.023	0.5	1.380	W06H8.1C	
41.8	30.3	0.009	0.5	1.380	F07C6.1	LIM domains
108	78.3	0.044	0.5	1.379	CEK035BXR_rc	
900.8	653.3	0.017	0.5	1.379	Y50D7A.7	
2178.6	1580.7	0.022	0.5	1.378	Y38H6C.1	
59.4	43.1	0.031	0.5	1.378	C50H11.15	cytochrome P450
2272.8	1649.3	0.009	0.5	1.378	H28O16.1	ATP synthase alpha and beta subunits, ATP synthase Alpha chain, C terminal
29.9	21.7	0.045	0.5	1.378	Y41D4B.P	
1642.1	1192.4	0.038	0.5	1.377	F38A3.2	ram-2 - (abnormal RAy Morphology) collagen
956.4	694.5	0.009	0.5	1.377	B0336.2	arf-1.2 - (ADP-Ribosylation Factor related)
488	354.4	0.008	0.5	1.377	R08E3.1	
149.8	108.8	0.018	0.5	1.377	R12E2.4	
36.2	26.3	0.013	0.5	1.376	F39C12.2	
174.9	127.1	0.018	0.5	1.376	W09D6.5	

37.7	27.4	0.012	0.5	1.376	F23F12.11	
409.5	297.7	0.009	0.5	1.376	F25B4.1	aminomethytransferase
45.8	33.3	0.021	0.5	1.375	Y6E2A.6	
78.1	56.8	0.008	0.5	1.375	M05D6.4	esterase
39.6	28.8	0.01	0.5	1.375	ZK455.6	Zinc finger, C4 type (two domains)
52.8	38.4	0.025	0.5	1.375	Y34B4A.A	
161	117.1	0.019	0.5	1.375	AV176402_rc	
153.3	111.5	0.027	0.5	1.375	AV181961_rc	
41.1	29.9	0.019	0.5	1.375	F19C6.1	G protein-coupled receptor kinase
41.5	30.2	0.013	0.5	1.374	T17A3.2	
627.9	457	0.03	0.5	1.374	Y111B2A.18	
2307.1	1679.4	0.011	0.5	1.374	F54C9.5	60S ribosomal protein L5
2764.8	2012.6	0.035	0.5	1.374	C66975	
1011.1	736.2	0.02	0.5	1.373	F07H5.5	
40.1	29.2	0.018	0.5	1.373	B0303.14	Giant secretory protein I-C, related to Sc adaptin-like subunit of the clathrin associ
127.3	92.7	0.009	0.5	1.373	T05H4.4	NADH-cytochrome B5 reductase
48.2	35.1	0.017	0.5	1.373	F32B4.4	RNA recognition motif. (aka RRM, RBD, or RNP domain)
44.9	32.7	0.044	0.5	1.373	D1009.2	
31.3	22.8	0.019	0.5	1.373	C50F2.8	
1311.7	955.7	0.009	0.5	1.373	C07A12.4	protein disulfide isomerase
2624.7	1912.9	0.009	0.5	1.372	F56F3.5	Ribosomal protein S3a (human) homolog.
911.4	664.5	0.009	0.5	1.372	C08F11.8	UDP-glucoronosyl and UDP-glucosyl transferases
150.3	109.6	0.034	0.5	1.371	C06G8.3	
195	142.2	0.009	0.5	1.371	AU116285_rc	
48.4	35.3	0.021	0.5	1.371	ZC404.1	
735.1	536.2	0.013	0.5	1.371	T24B8.5	
282.9	206.4	0.01	0.5	1.371	Y54E10A.16	
42.9	31.3	0.013	0.5	1.371	Y82E9BL.M	
423	308.7	0.011	0.5	1.370	T02E1.5	3-oxoacyl-[acyl-carrier protein] reductase
552.2	403.1	0.026	0.5	1.370	Y17G7B.7	Triosephosphate isomerase
38.9	28.4	0.015	0.5	1.370	F11C1.2	
1611.2	1176.6	0.01	0.5	1.369	F57F4.4	
1924.3	1405.3	0.023	0.5	1.369	Y57G11C.16	ribosomal protein S13
37.9	27.7	0.028	0.5	1.368	W10G11.9	chemoreceptor
65.8	48.1	0.024	0.5	1.368	C07A4.2	Defense-related protein containing SCP domain
52.8	38.6	0.006	0.5	1.368	Y53F4B.2	
160.1	117.1	0.012	0.5	1.367	R05D7.3	
569.4	416.5	0.026	0.5	1.367	C42D8.8	

33.9	24.8	0.02	0.5	1.367	ZK380.4	
1046.4	765.6	0.009	0.5	1.367	F01G10.1	transketolase
80.5	58.9	0.009	0.5	1.367	C01H6.4	
48.5	35.5	0.007	0.5	1.366	K08E4.3	
610.3	446.9	0.028	0.5	1.366	C29F5.1	
2491.4	1824.4	0.011	0.5	1.366	C32E8.2	ribosomal protein L13
32.5	23.8	0.019	0.5	1.366	CEK042A5R_rc	
52.7	38.6	0.006	0.5	1.365	Y71H2AM.M	
31.8	23.3	0.02	0.5	1.365	B0280.4	
53.5	39.2	0.042	0.5	1.365	C34F6.7	
565	414	0.026	0.5	1.365	R74.3	xbp-1- (X box DNA binding protein-1)
91.3	66.9	0.01	0.5	1.365	F45H7.2	
3252.1	2383.2	0.014	0.5	1.365	C29F4.1	col-125 - (COLlagen)
80.5	59	0.011	0.5	1.364	F43B10.2	
101.1	74.1	0.012	0.5	1.364	F21F8.11	
33.7	24.7	0.024	0.5	1.364	C39F7.1	
98.5	72.2	0.01	0.5	1.364	C07G3.9	
25.1	18.4	0.04	0.5	1.364	R04E5.2	
83.6	61.3	0.01	0.5	1.364	Y45F10D.11	
25.5	18.7	0.042	0.4	1.364	Y46H3C.2	
199.7	146.6	0.012	0.5	1.362	C05E11.1	
490.5	360.2	0.028	0.5	1.362	F56F10.1	peptidase
50.5	37.1	0.031	0.5	1.361	Y37A1B.11	potassium channel protein
63.7	46.8	0.03	0.5	1.361	C02H6.2	
73.9	54.3	0.041	0.5	1.361	C55A1.9	
41.1	30.2	0.04	0.5	1.361	Y48E1B.6	
62.6	46	0.032	0.4	1.361	F37E3.1	ncbp-1 - (Nuclear Cap Binding Protein)
115.4	84.8	0.033	0.5	1.361	C07H6.6	
784.7	576.7	0.011	0.4	1.361	T12A2.2	
41.5	30.5	0.031	0.4	1.361	Y54F10AR.B_rc	
94.5	69.5	0.032	0.4	1.360	F47A4.2	
103.6	76.2	0.028	0.4	1.360	T25G12.6	
137.7	101.3	0.038	0.4	1.359	Y56A3A.12B	
725.3	533.6	0.01	0.4	1.359	T08H10.1	aldose reductase
59.8	44	0.037	0.4	1.359	F41B5.9	nuclear hormone receptor
144.6	106.4	0.013	0.4	1.359	K04A8.1	
40.9	30.1	0.014	0.4	1.359	B0361.7	Acid phosphatase
240.5	177	0.041	0.4	1.359	M03A1.6	

29.2	21.5	0.031	0.4	1.358	F59B2.13	possible G protein coupled receptor
1925	1417.5	0.011	0.4	1.358	F22A3.6	
38.7	28.5	0.015	0.4	1.358	W01B6.3	tetracyclin resistance protein like
3355.7	2471.8	0.012	0.4	1.358	B0222.8	
101.9	75.1	0.021	0.4	1.357	K07C11.4	esterase
96.6	71.2	0.01	0.4	1.357	F40F8.3	
1894.2	1396.4	0.041	0.4	1.356	F36A4.10	col-34 - (COLlagen)
1251	922.5	0.049	0.4	1.356	AV176170_rc	
386.6	285.3	0.011	0.4	1.355	F35C8.5	
2453.9	1813.6	0.041	0.4	1.353	F11G11.10	col-17- (COLlagen)
123.8	91.5	0.018	0.4	1.353	F07D3.1	
48.7	36	0.046	0.4	1.353	W08E12.6	
357.4	264.2	0.049	0.4	1.353	W04G3.5	ribose-phosphate pyrophosphokinase
71.9	53.2	0.023	0.4	1.352	Y113G7B.19	
52.3	38.7	0.011	0.4	1.351	F18A11.4	
72.7	53.8	0.032	0.4	1.351	W05H7.4	
27.7	20.5	0.036	0.4	1.351	Y53F4B.1	
83.2	61.6	0.013	0.4	1.351	C37E2.5	Homeobox domain
2604.9	1929.9	0.013	0.4	1.350	F13B10.2	60S ribosomal protein L3
49.4	36.6	0.023	0.4	1.350	Y57G11C.22	perinuclear binding protein
38.6	28.6	0.016	0.4	1.350	F25C8.2	
30.9	22.9	0.033	0.4	1.349	T27B7.1	
137.9	102.2	0.028	0.4	1.349	Y77E11A.7	
71.1	52.7	0.017	0.4	1.349	Y73B6BL.H	
983.8	729.4	0.045	0.4	1.349	Y45G12C.2	
37.9	28.1	0.017	0.4	1.349	M01D1.7	
94.8	70.3	0.042	0.4	1.349	T02H6.9	
95.8	71.1	0.039	0.4	1.347	ZK154.4	
140.8	104.5	0.032	0.4	1.347	F42H10.4	
641.5	476.2	0.035	0.4	1.347	Y43C5A.2	Fibrinogen beta and gamma chains, C-terminal globular domain
2420.6	1797.1	0.013	0.4	1.347	Y73B3A.D	
618.2	459	0.024	0.4	1.347	F35E12.6	
403.7	299.8	0.014	0.4	1.347	F46G10.5	ptr-24 - (PaTched Related family)
28.8	21.4	0.04	0.4	1.346	F27E11.1	nucleoside transporter
221.9	164.9	0.021	0.4	1.346	F25D1.1	protein phosphatase
734	545.5	0.012	0.4	1.346	K05C4.2	
99.7	74.1	0.016	0.4	1.345	F54B8.6	
285.1	211.9	0.047	0.4	1.345	T10B10.2	ubiquinol-cytochrome c reductase complex core protein 2

30	22.3	0.048	0.4	1.345	F18E2.4	
33.9	25.2	0.025	0.4	1.345	B0524.4	
1328	987.2	0.026	0.4	1.345	F46F11.5	vacuolar ATPase G subunit
227.7	169.3	0.03	0.4	1.345	F35F10.12	
400.5	297.8	0.046	0.4	1.345	F45D3.5	sel-1 - (Suppressor/Enhancer of Lin-12)
47.2	35.1	0.012	0.4	1.345	F47H4.10	
36.7	27.3	0.019	0.4	1.344	Y6B3B.9	
75	55.8	0.039	0.4	1.344	Y111B2A.11	
2021.6	1504.4	0.012	0.4	1.344	K02F2.2	ahcy-1 - (S-AdenosylhomoCysteine HYdrolase homolog)
776.4	577.8	0.021	0.4	1.344	C54G4.8	cytochrome C1, heme protein
386	287.3	0.023	0.4	1.344	Y71G12B.T	
131.6	98	0.013	0.4	1.343	B0379.5	
28.2	21	0.036	0.4	1.343	F18C5.8	
81.1	60.4	0.014	0.4	1.343	C49C3.10A	serine/threonine kinase
3273.4	2438	0.013	0.4	1.343	T10E10.1	col-168 - (COLlagen)
236.4	176.1	0.035	0.4	1.342	F08A8.1	Acyl-CoA oxidase
914.1	681.1	0.014	0.4	1.342	F22F7.1	
69.1	51.5	0.043	0.4	1.342	F28H6.1	akt-2 - (AKT kinase family)
44	32.8	0.038	0.4	1.341	C06E2.5	
707.8	527.7	0.012	0.4	1.341	Y38F2AR.E	
34.2	25.5	0.023	0.4	1.341	M110.2	
209.1	156	0.015	0.4	1.340	ZK418.5	
3267.5	2437.9	0.014	0.4	1.340	T10E10.2	col-167 - (COLlagen)
1718.6	1282.5	0.014	0.4	1.340	Y45F10D.12	Eukaryotic ribosomal protein L18
86.7	64.7	0.014	0.4	1.340	Y41D4B.Y	
751.1	560.6	0.016	0.4	1.340	T19B4.3	adenine phosphoribosyltransferase
90.8	67.8	0.04	0.4	1.339	Y51H7C.6A	
42.3	31.6	0.016	0.4	1.339	R06C1.3	
2300	1718.8	0.014	0.4	1.338	F31E3.5	Elongation factor 1-alpha
38.4	28.7	0.041	0.4	1.338	T27B7.3	zinc finger protein
78.4	58.6	0.049	0.4	1.338	Y34B4A.G	
198.4	148.3	0.018	0.4	1.338	R13A5.5	
384.8	287.7	0.046	0.4	1.338	C26C9.2	
2070.2	1547.9	0.014	0.4	1.337	ZK1010.1	UBQ-2 ubiquitin; 60S Ribosomal protein L40
2191.7	1639.5	0.015	0.4	1.337	F39B2.6	40S ribosomal protein S26
1692.8	1266.6	0.014	0.4	1.336	F01G10.1	transketolase
1960.2	1468.3	0.016	0.4	1.335	D1007.6	40S ribosomal protein S10
26.7	20	0.047	0.4	1.335	Y48G1C.C	

1134.6	849.9	0.031	0.4	1.335	B0365.3	Na(+)/K(+) ATPase alpha subunit
748.5	560.7	0.031	0.4	1.335	C29F3.7	
374.3	280.4	0.029	0.4	1.335	F11G11.3	Glutathione S-transferase
34.3	25.7	0.025	0.4	1.335	Y49C4A.1	
84.4	63.3	0.018	0.4	1.333	F57G9.1	
27.6	20.7	0.048	0.4	1.333	Y43F11A.1	
2777.4	2084	0.022	0.4	1.333	W05B2.1	
66.5	49.9	0.015	0.4	1.333	T16G1.1	
1828.1	1371.9	0.015	0.4	1.333	C09D4.5	60S ribosomal protein L19
439.9	330.3	0.015	0.4	1.332	F26E4.12	glutathione peroxidase
28.5	21.4	0.041	0.4	1.332	T01G6.2	zinc finger protein
2016.2	1514.3	0.016	0.4	1.331	CEK082GZR_rc	
621	466.6	0.016	0.4	1.331	Y105C5B.28	
33.8	25.4	0.036	0.4	1.331	K05B2.2	
83.7	62.9	0.021	0.4	1.331	Y47C4A.A	
757	569	0.016	0.4	1.330	AV202808	
2114	1589	0.016	0.4	1.330	T01C3.6	40S ribosomal protein S16
270.8	203.6	0.028	0.4	1.330	g3047192	
1468.1	1103.8	0.021	0.4	1.330	C55576_rc	
64.6	48.6	0.049	0.4	1.329	F13E9.7	reverse transcriptase
2745	2065.6	0.022	0.4	1.329	F15H10.1	collagen
28.7	21.6	0.043	0.4	1.329	F10A3.8	
2536.5	1909.3	0.016	0.4	1.328	B0250.1	Ribosomal Proteins L2
2026.5	1525.5	0.021	0.4	1.328	Y39B6B.G	
1917.6	1443.6	0.017	0.4	1.328	Y106G6H.2	RNA recognition motif. (aka RRM, RBD, or RNP domain) ; Poly-adenylate binding
1030.2	775.6	0.015	0.4	1.328	F55H2.2	Membrane-associated atpase gamma chain
59.5	44.8	0.04	0.4	1.328	F41E6.1	
392.7	295.8	0.033	0.4	1.328	F53F10.4	RAS-related protein
28.4	21.4	0.042	0.4	1.327	F52E4.4	
960.3	723.8	0.016	0.4	1.327	C07G2.3	TCP-1 like chaperonin
53.2	40.1	0.012	0.4	1.327	R11.4	
2118.7	1597	0.016	0.4	1.327	F28D1.7	ribosomal protein S23
152.7	115.1	0.047	0.4	1.327	F07A11.4	ubiquitin carboxyl-terminal hydrolase
1830.5	1380	0.018	0.4	1.326	F25H5.4	Elongation factor Tu family (contains ATP/GTP binding P-loop)
1406.2	1060.6	0.019	0.4	1.326	R07H5.8	adenosine kinase
149	112.4	0.036	0.4	1.326	F55A11.3	Zinc finger, C3HC4 type (RING finger)
839.8	634	0.036	0.4	1.325	Y18D10A.19C	FKBP-type peptidyl-prolyl cis-trans isomerases
96.8	73.1	0.044	0.4	1.324	C15C7.1	

555.6	419.6	0.038	0.4	1.324	T22B11.5	2-oxoglutarate dehydrogenase
157.8	119.2	0.038	0.4	1.324	F10E9.6	
31.9	24.1	0.035	0.4	1.324	T06A1.2	chemoreceptor
629.3	475.8	0.017	0.4	1.323	C33H5.16	protein-tyrosine phophatase
41.1	31.1	0.049	0.4	1.322	T10B10.3	
815.5	617.1	0.019	0.4	1.322	ZK455.1	iron-responsive element-binding like protein
51.8	39.2	0.013	0.4	1.321	F56A11.3	
241.8	183	0.041	0.4	1.321	B0286.4	
891.4	674.7	0.045	0.4	1.321	CEK094H4R_rc	
2234.6	1691.5	0.017	0.4	1.321	H06I04.4	ubl-1 - (UBiquitin-Like family)
38.7	29.3	0.024	0.4	1.321	C53130_rc	
65.9	49.9	0.017	0.4	1.321	C56A3.7	cav-2 - (CAVeolin)
508.7	385.2	0.023	0.4	1.321	C47E12.5	ubiquitin-activating enzyme
1815.3	1374.6	0.018	0.4	1.321	B0513.3	60S ribosomal protein L29
862.6	653.2	0.015	0.4	1.321	R01E6.3	
78.3	59.3	0.038	0.4	1.320	Y67D8A.G	
1559.2	1180.9	0.018	0.4	1.320	R10E11.2	Vacuolar ATP synthase subunit
297.7	225.5	0.017	0.4	1.320	E04A4.5	
1918.6	1453.6	0.017	0.4	1.320	Y38A10A.5	calreticulin precursor
729.5	552.7	0.042	0.4	1.320	K02F3.4	
1500.8	1137.2	0.018	0.4	1.320	C47E8.5	daf-21 - (abnormal DAuer Formation), HSP90
33.1	25.1	0.036	0.4	1.319	Y39A3A.4	protein-tyrosine phosphatase
1187.2	900.4	0.019	0.4	1.319	Y39A1C.3	'Cold-shock' DNA-binding domain
85.7	65	0.025	0.4	1.318	CEC2531_rc	
2310.5	1753.2	0.017	0.4	1.318	Y71F9AL.13	
2221	1685.3	0.019	0.4	1.318	C49H3.11	
699.2	530.6	0.018	0.4	1.318	LLC1.2	
1489.3	1130.4	0.018	0.4	1.317	F07D10.1	ribosomal protein
743.9	564.8	0.018	0.4	1.317	Y38F2AR.M	
1916.8	1455.4	0.02	0.4	1.317	CEK080H1R	
1095.2	832	0.017	0.4	1.316	C32D5.2	sma-6 - (SMAll), type I TGF-beta receptor for DBL-1
1095.2	832	0.017	0.4	1.316	C17G10.5	lys-8 - (LYSozyme)
72.5	55.1	0.019	0.4	1.316	C32H11.13	
72.1	54.8	0.02	0.4	1.316	C33E10.5	
2028.9	1542.4	0.017	0.4	1.315	C54C6.1	rpl-37 - (Ribosomal Protein, Large subunit), 60S ribosomal protein L37
114.7	87.2	0.028	0.4	1.315	M02A10.3	sli-1 - (Suppressor of LIneage defect), zinc-finger protein
163.1	124	0.027	0.4	1.315	F39H11.2	transcription factor TFIID like
1270.8	966.7	0.019	0.4	1.315	H36L18.2	

34.7	26.4	0.035	0.4	1.314	C34B2.1	
31.8	24.2	0.043	0.4	1.314	K11D2.3	clathrin coat assembly protein
2167.3	1649.6	0.019	0.4	1.314	T05E11.1	40S ribosomal protein S5
239.9	182.6	0.019	0.4	1.314	T26C5.3A	Yeast YB70 like
187.2	142.5	0.018	0.4	1.314	Y60A3A.7	
1697.9	1292.5	0.02	0.4	1.314	F54C9.1	initiation factor 5A
53.2	40.5	0.027	0.4	1.314	ZC196.5	
893.4	680.4	0.018	0.4	1.313	VW06B3R.1	Insulinase (proteinase M16)
133	101.3	0.038	0.4	1.313	F22E5.13	
2583.4	1967.7	0.02	0.4	1.313	F52B11.4	col-133 - (COLlagen)
1951.8	1486.9	0.019	0.4	1.313	E04A4.7	cytochrome C
29.4	22.4	0.047	0.4	1.313	M02B7.5	
481.7	367.3	0.042	0.4	1.311	D2096.2	
1863.3	1421	0.029	0.4	1.311	C34E10.6	atp-2 - (ATP synthase subunit)
39.6	30.2	0.049	0.4	1.311	K06B4.10	Zinc finger, C4 type (two domains) (2 domains)
47.2	36	0.02	0.4	1.311	C29640_rc	
3051.2	2327.4	0.025	0.4	1.311	F55C10.2	collagen
40.9	31.2	0.025	0.4	1.311	C53D5.1	
132.4	101	0.032	0.4	1.311	C10G8.8	
50.6	38.6	0.025	0.4	1.311	C24G6.4	nhr-47 zinc finger protein
240.4	183.4	0.031	0.4	1.311	C03A3.2	
297.1	226.7	0.019	0.4	1.311	Y39E4A.3	Transketolase
964.7	736.4	0.019	0.4	1.310	C08H9.2	high-density lipoprotein-binding protein
124.4	95	0.046	0.4	1.309	Y55F3BR.C	
34.3	26.2	0.037	0.4	1.309	F59G1.4	
1336.5	1020.9	0.02	0.4	1.309	W07B8.5	thiol protease
172	131.4	0.022	0.4	1.309	M176.2	glutathione synthase
893.2	682.6	0.021	0.4	1.309	F01F1.8	
690.9	528	0.021	0.4	1.309	F57B9.3	phi-2 Eukaryotic initiation factor 4A
1923.5	1470.1	0.031	0.4	1.308	F42C5.8	40S ribosomal protein S8
124.9	95.5	0.034	0.4	1.308	C41D11.7	DNA-binding protein
1197.5	915.7	0.043	0.4	1.308	K11D9.2B	E1-E2 ATPases
71.4	54.6	0.039	0.4	1.308	C33G3.4	beta-mannosidase
52.3	40	0.025	0.4	1.308	F16B4.1	zinc finger protein
52.3	40	0.047	0.4	1.308	T05C1.6	
265.2	202.9	0.021	0.4	1.307	K10D2.6	NADPH-cytochrome P450
2386	1826.2	0.02	0.4	1.307	D1007.12	60S ribosomal protein L24
236.7	181.2	0.034	0.4	1.306	C14F11.3	

122.9	94.1	0.045	0.4	1.306	R11F4.1	Glycerol kinase
233.8	179.1	0.024	0.4	1.305	C06H5.6	
48.3	37	0.032	0.4	1.305	C08G5.3	
61.2	46.9	0.03	0.4	1.305	F59F5.1	
334.7	256.5	0.047	0.4	1.305	C17H12.1	
1603.8	1229.4	0.022	0.4	1.305	F17C11.9	elongation factor 1-gamma
955.9	732.8	0.029	0.4	1.304	C03B1.12	
2816.7	2159.9	0.029	0.4	1.304	F15H10.2	collagen
161.4	123.8	0.021	0.4	1.304	F56A11.5	
151.6	116.3	0.034	0.4	1.304	F56C9.8	
702.9	539.3	0.03	0.4	1.303	F34H10.1	ubiquitin/ribosomal protein
37.4	28.7	0.033	0.4	1.303	B0454.9	
86	66	0.028	0.4	1.303	Y48E1B.3	geranylgeranyl transferase beta subunit
681.2	522.8	0.042	0.4	1.303	ZK1307.8	protein kinase C substrate
838.7	643.8	0.019	0.4	1.303	C56G2.6	
53.8	41.3	0.018	0.4	1.303	D1005.4	
2208.6	1695.5	0.022	0.4	1.303	F25H2.5	nucleoside diphosphate kinase
35.3	27.1	0.039	0.4	1.303	F53B7.2	G-protein coupled receptor
1278	981.7	0.023	0.4	1.302	D2096.8	
1687.1	1296	0.025	0.4	1.302	M03F4.2	actin
1866.9	1434.2	0.024	0.4	1.302	F25H2.11	TCTP protein
589.4	452.8	0.031	0.4	1.302	ZK1320.2	
132.5	101.8	0.031	0.4	1.302	C28G1.3	
953.8	732.9	0.021	0.4	1.301	Y57G11C.12	NADH-ubiquinone oxidoreductase
683.6	525.3	0.021	0.4	1.301	T07C12.7	Transthyretin-like family
51.4	39.5	0.02	0.4	1.301	C24A3.4	E. coli L-carnitine dehydratase
2033.5	1563.3	0.023	0.4	1.301	F53G12.10	ribosomal protein
117.7	90.5	0.035	0.4	1.301	F28B3.1	
1078.5	829.4	0.02	0.4	1.300	F52D10.3	ftt-2- (14-3-3 protein)
1570	1207.4	0.038	0.4	1.300	F54D11.1	pmt-2- Phosphoethanolamine MethylTransferase
76.7	59	0.024	0.4	1.300	D2085.7	
65.1	50.1	0.048	0.4	1.299	C34D1.5	Basic region plus leucine zipper transcription factors
1875.9	1444	0.023	0.4	1.299	F02A9.3	
62.2	47.9	0.044	0.4	1.299	H25P06.1	Hexokinases
45.3	34.9	0.045	0.4	1.298	M03F4.8	
1060.5	817.2	0.022	0.4	1.298	F28B4.3	EGF-like repeat
82.4	63.5	0.03	0.4	1.298	W09G10.1	collagen
1310	1009.6	0.025	0.4	1.298	F25B5.7	

93.4	72	0.045	0.4	1.297	Y53H1C.1	
32.3	24.9	0.048	0.4	1.297	C39E9.5	scl-7 testis-specific protein TPX-1 like -ECM Defense-related protein containing SC
45.4	35	0.027	0.4	1.297	R07E4.1	
391.4	301.8	0.036	0.4	1.297	ZK632.6	Calnexin
1769.2	1364.5	0.024	0.4	1.297	F37C12.9	Ribosomal protein S14
1736.5	1339.5	0.03	0.4	1.296	F10B5.1	ribosomal protein L10 (QM protein)
118.1	91.1	0.031	0.4	1.296	Y39A1C.2	HECT-domain (ubiquitin-transferase).
322	248.4	0.026	0.4	1.296	T24H10.2	BZIP protein
35	27	0.042	0.4	1.296	Y45F10A.4	
428.3	330.5	0.025	0.4	1.296	K07D4.3	
774.8	598	0.026	0.4	1.296	M7.1	ubiquitin-conjugating enzyme E2-17 KD
88.1	68	0.039	0.4	1.296	F17E5.1A	lin-2 -MAGUK protein, calcium calmodulin dependent protein kinase
253.9	196.1	0.028	0.4	1.295	AU115072	
2295.7	1773.6	0.025	0.4	1.294	C23G10.3	Ribosomal protein S3
38.7	29.9	0.036	0.4	1.294	K04F1.10	
69.1	53.4	0.027	0.4	1.294	F42G2.2	
1646.6	1272.5	0.043	0.4	1.294	F46H5.3	arginine kinase
42.7	33	0.044	0.4	1.294	F53B1.6	
1407.1	1087.7	0.023	0.4	1.294	T05E11.3	endoplasmin precursor (GRP94)
2548.9	1970.5	0.033	0.4	1.294	F57B1.4	collagen
749.6	579.6	0.026	0.4	1.293	T05F1.1	
2275.7	1759.8	0.026	0.4	1.293	F25H2.10	rpa-0 - (Replication Protein A homolog)
1286.8	995.1	0.024	0.4	1.293	T20D3.2	
83.9	64.9	0.024	0.4	1.293	F08B6.5	
307.6	238	0.043	0.4	1.292	C52E4.5	alpha 1,2, mannosidase
2222.2	1719.5	0.028	0.4	1.292	K11H12.2	60S ribosomal protein L15
319.3	247.1	0.026	0.4	1.292	ZK484.3	
40.3	31.2	0.035	0.4	1.292	C14B4.1	
582.1	450.7	0.024	0.4	1.292	C47B2.4	Proteasome A-type and B-type
52.3	40.5	0.023	0.4	1.291	ZK1248.2	
99.4	77	0.042	0.4	1.291	F38A6.2	WD domain, G-beta repeat
307	237.9	0.033	0.4	1.290	Y71H10B.1	
53.8	41.7	0.044	0.4	1.290	F17C11.5	
390.4	302.7	0.026	0.4	1.290	F48E3.3	killer toxin-resistance protein (S. cerevisiae)
794.3	616	0.023	0.4	1.289	B0491.5	
373.8	289.9	0.033	0.4	1.289	R12E2.9	
1320.9	1024.6	0.026	0.4	1.289	T20G5.2	Citrate synthase
1002.7	777.8	0.025	0.4	1.289	C30F8.2	

1621.6	1258.2	0.027	0.4	1.289	ZK829.4	glutamate dehydrogenase
1024.5	795	0.026	0.4	1.289	F53F4.10	NADH-ubiquinone dehydrogenase 24 KD subunit
2126.7	1650.3	0.029	0.4	1.289	Y24D9A.D	
1782.5	1383.3	0.026	0.4	1.289	AV199132_rc	
318	246.8	0.026	0.4	1.288	B0416.5	
1745.7	1354.9	0.029	0.4	1.288	F26D10.3	HSP-1 heat shock 70kd protein A
2263.1	1756.6	0.027	0.4	1.288	W09C5.6B	Ribosomal protein L31e
1693.8	1314.8	0.025	0.4	1.288	F55D10.2	Ribosomal protein L23
39.8	30.9	0.044	0.4	1.288	Y39A3CL.2	
1546.9	1201.2	0.028	0.4	1.288	F01G4.6	phosphate carrier protein precursor
272.1	211.3	0.025	0.4	1.288	F18F11.1	
1748.5	1358	0.029	0.4	1.288	ZC434.2	40S ribosomal protein S7
88.3	68.6	0.034	0.4	1.287	D2013.10	
54.7	42.5	0.033	0.4	1.287	K09A9.4	ubiquitin C-terminal hydrolase
54.3	42.2	0.032	0.4	1.287	Y23B4A.2	
493.7	383.7	0.049	0.4	1.287	W08E3.3	GTP-binding protein like
588.9	457.7	0.024	0.4	1.287	C23H3.4	aminotransferase
1991.2	1547.9	0.029	0.4	1.286	K04D7.1	guanine nucleotide-binding protein
1366.7	1063.1	0.03	0.4	1.286	T14B4.6	dpy-2- cuticular collagen
2315.1	1801.2	0.028	0.4	1.285	E04A4.8	ribosomal protein
1481	1152.3	0.028	0.4	1.285	C14B9.7	Ribosomal protein L21
2249.1	1750	0.027	0.4	1.285	C23G10.3	Ribosomal protein S3
767.2	597	0.03	0.4	1.285	B0403.4	protein disulfide-isomerase
2169.6	1688.3	0.027	0.4	1.285	R13A5.8	Ribosomal protein L9
1430.5	1113.6	0.028	0.4	1.285	F21F8.3	asp-5- protease
1668.9	1299.4	0.03	0.4	1.284	H22K11.1	asp-3- aspartyl protease
755.2	588	0.027	0.4	1.284	C30868_rc	pyp-1 - (inorganic PYroPhosphatase)
107.1	83.4	0.027	0.4	1.284	T20F7.6	
239.6	186.6	0.029	0.4	1.284	Y110A7A.6	phosphofructokinase
167.8	130.7	0.029	0.4	1.284	C09F5.2	
2073.4	1615.9	0.028	0.4	1.283	F53A3.3	40S ribosomal protein
2280.2	1777.3	0.035	0.4	1.283	C36E6.B	
1185.4	924	0.027	0.4	1.283	F13D12.6	serine carboxypeptidase
67.2	52.4	0.037	0.4	1.282	D1054.5	
44.5	34.7	0.044	0.4	1.282	F16B12.7	serine/threonine kinase
783.4	611	0.03	0.4	1.282	R05F9.12	
2019.1	1574.9	0.032	0.4	1.282	T27E9.1	ADP/ATP carrier protein
1890.6	1474.9	0.029	0.4	1.282	M01F1.2	L13P family ribosomal protein

895.4	698.7	0.026	0.4	1.282	AV200549_rc	
1828.3	1426.8	0.032	0.4	1.281	B0393.1	40S ribosomal protein
275.2	214.8	0.046	0.4	1.281	F45D3.5	Yeast hypothetical gene L8167.5 like
2073.4	1618.4	0.027	0.4	1.281	F40F11.1	ribosomal protein S11
1645.4	1284.4	0.028	0.4	1.281	R11D1.8	ribsomal protein L28 like
699.4	546	0.036	0.4	1.281	F11E6.5	GNS1/SUR4 family
633.3	494.4	0.032	0.4	1.281	C38C3.5A	actin depolymerizing factor
68.4	53.4	0.04	0.4	1.281	Y113G7B.11	
1502.7	1173.4	0.029	0.4	1.281	F20B6.2	vacuolar ATP synthase (strong)
2047.4	1598.8	0.028	0.4	1.281	C37A2.7	
207.7	162.2	0.05	0.4	1.281	F36G3.2	
2360.2	1843.6	0.03	0.4	1.280	Y106G6H.3	
364.2	284.5	0.031	0.4	1.280	C46803	
2190	1711.2	0.03	0.4	1.280	T04C12.5	actin
160.2	125.2	0.041	0.4	1.280	Y32F6A.3	pap-1 - (Poly-A Polymerase)
2311.7	1807	0.03	0.4	1.279	Y71A12B.1	40S ribosomal protein S6
1233.2	964.2	0.03	0.4	1.279	g7716071	
2141.2	1674.6	0.028	0.4	1.279	C06B8.8	
930.2	727.7	0.028	0.4	1.278	F41H10.7	elo-5 - (fatty acid ELOngation), polyunsaturated fatty acid (PUFA) elongase
107.5	84.1	0.03	0.4	1.278	C45G9.5	
289.9	226.8	0.03	0.4	1.278	Y62E10A.11B	
182	142.4	0.034	0.4	1.278	C01F6.4	sex-determining protein (FEM-3)- in herm and male spermatids
914.2	715.3	0.032	0.4	1.278	K01C8.10	cct-4 - (Chaperonin Containing TCP-1)
578.2	452.7	0.028	0.4	1.277	F56C9.7	
2467.4	1932	0.03	0.4	1.277	T08B2.10	40S ribosomal protein S17
108.8	85.2	0.046	0.4	1.277	F54D10.3	
629.3	492.8	0.036	0.4	1.277	R10H10.3	CUB domain, von Willebrand factor type A domain
1943.6	1522.1	0.031	0.4	1.277	C26F1.4	ribosomal protein/ubiquitin-like protein
1017.6	797	0.03	0.4	1.277	T03F1.3	phosphoglycerate kinase
60	47	0.031	0.4	1.277	C17F3.2	
1392.3	1090.7	0.03	0.4	1.277	F31C3.1	cyclophillin
143.6	112.5	0.039	0.4	1.276	g7963656	
47.1	36.9	0.035	0.4	1.276	Y105C5B.13	
441.5	345.9	0.03	0.4	1.276	R155.1	
1391.5	1091	0.033	0.4	1.275	R05G6.7	
1257.7	986.1	0.031	0.4	1.275	F44E5.1	
146.4	114.8	0.044	0.4	1.275	K07G5.1	Leucine Rich Repeat (2 copies) (4 domains)
1998.5	1567.4	0.032	0.4	1.275	T05F1.3	Ribosomal protein S19e

123.5	96.9	0.031	0.4	1.275	T03G11.6	
137	107.5	0.039	0.4	1.274	D1044.1	
38.1	29.9	0.048	0.4	1.274	ZC21.6	
1396.6	1096.1	0.034	0.4	1.274	Y105E8B.S	
476.4	373.9	0.04	0.4	1.274	Y37D8A.16	
1455.4	1142.3	0.033	0.4	1.274	Y65B4BR.5	
1951.9	1533.1	0.036	0.4	1.273	F09F7.2	
58.3	45.8	0.035	0.4	1.273	Y5H2B.2	
2101.6	1651.1	0.033	0.4	1.273	C68152	
1965.1	1543.9	0.034	0.4	1.273	F10B5.1	ribosomal protein L10 (QM protein)
1216.3	955.6	0.031	0.4	1.273	C17H12.14	ATPase
722	567.3	0.032	0.4	1.273	T05C12.7	T-complex protein
512	402.4	0.05	0.4	1.272	W08D2.6	collagen
1330.2	1045.5	0.034	0.4	1.272	T18H9.2	asp-2 protease
66.9	52.6	0.034	0.4	1.272	R07C3.1	
2039.4	1604.1	0.032	0.4	1.271	C09H10.2	60S ribosomal protein
1238.6	974.5	0.046	0.4	1.271	F32D1.2	ATP synthase epsilion chain
1588	1249.4	0.035	0.4	1.271	F26E4.9	cytochrome C oxidase
511.9	402.8	0.032	0.4	1.271	ZK1321.3	
620.1	488.1	0.034	0.4	1.270	C47E12.4	inorganic pyrophosphatase
137.9	108.6	0.038	0.3	1.270	Y38C1AA.J	
334.3	263.3	0.032	0.3	1.270	ZK945.2	proteasome component (A-type)
58.4	46	0.039	0.4	1.270	F57C7.1B	female sterile homeotic protein (Bromodomain protein)
1976.3	1557.1	0.034	0.3	1.269	C26F1.9	ribosomal protein L39
107.6	84.8	0.049	0.3	1.269	T27A3.6	
39.7	31.3	0.05	0.3	1.268	F35E8.7	
231.6	182.6	0.047	0.3	1.268	C27H5.4	
1374.2	1084.2	0.037	0.3	1.267	F25B5.4	
1225	966.5	0.035	0.3	1.267	F57B9.6	
2058.9	1624.5	0.039	0.3	1.267	F02A9.2	far-1- (fatty acid/retinol binding protein)
1927.3	1521.6	0.035	0.3	1.267	Y41D4B.W	
1910.5	1508.7	0.034	0.3	1.266	F28D1.7	rps-23 - (Ribosomal Protein, Small subunit)
168.4	133	0.043	0.3	1.266	F56G4.5	png-1 - (PNG (Peptide:N-Glycanase) homolog)
521.6	412.1	0.036	0.3	1.266	T10B5.5	cct-7 - (Chaperonin Containing TCP-1)
2202.1	1739.9	0.034	0.3	1.266	C27A2.2	ribosomal protein L22
614.9	486.3	0.035	0.3	1.264	K07A3.1	fructose-bisphosphatase
1396.1	1105	0.037	0.3	1.263	F36A2.7	
1714.1	1358.1	0.038	0.3	1.262	F54D8.2	Cytochrome C oxidase

1899.7	1505.3	0.037	0.3	1.262	F54E7.2	
1168.5	926.1	0.036	0.3	1.262	F32D8.6	protein transport protein SEC61 gamma subunit
53.1	42.1	0.037	0.3	1.261	Y71G12B.N	
1027.9	815.3	0.047	0.3	1.261	F57H12.1	GTP-binding protein
1231.5	976.9	0.037	0.3	1.261	F56H11.4	Yeast YJT6 like
517.4	410.5	0.039	0.3	1.260	T25B9.9	6-phosphogluconate dehydrogenase
2124.1	1685.3	0.039	0.3	1.260	K02B2.5	ribosomal protein
1429.7	1135.2	0.038	0.3	1.259	C59877_rc	
2175.3	1727.4	0.041	0.3	1.259	F40F8.10	ribosomal protein S9
1500.4	1191.8	0.04	0.3	1.259	Y65B4BR.5	
311.7	247.6	0.039	0.3	1.259	K02F3.10	
433.8	344.6	0.04	0.3	1.259	R05F9.10	sgt-1 - (Small Glutamine-rich Tetratrico repeat protein), TPR domain repeats
66.8	53.1	0.046	0.3	1.258	F43D2.2	
724.6	576	0.038	0.3	1.258	Y19D2B.1	
412.6	328.1	0.044	0.3	1.258	R12E2.13	
397.8	316.4	0.041	0.3	1.257	B0280.3	
1006.8	800.8	0.041	0.3	1.257	T25C12.3	EGF-repeats
1439	1144.6	0.041	0.3	1.257	C36E8.5	beta tubulin
736.2	585.6	0.037	0.3	1.257	K08C9.7	Ubiquitin family
2578.9	2051.7	0.039	0.3	1.257	F52B5.6	60S ribosomal protein
135.6	107.9	0.042	0.3	1.257	ZK470.5	
1429.9	1138	0.04	0.3	1.257	C53B7.4	ATP synthase
53.4	42.5	0.048	0.3	1.256	T12D8.3	acbp-5 - (Acyl-Coenzyme A Binding Protein)
1537.3	1223.8	0.039	0.3	1.256	Y71H2AM.S	
1355.7	1079.7	0.039	0.3	1.256	T05G5.6	Enoyl-CoA hydratase
782	622.9	0.043	0.3	1.255	R11A8.6	irs-1 isoleucyl-tRNA synthetase
446.9	356	0.042	0.3	1.255	F46C5.9	G-protein
146.8	117	0.046	0.3	1.255	M01A8.1	
942.9	751.5	0.038	0.3	1.255	Y71F9AM.A	
1077.8	859.1	0.045	0.3	1.255	F59B1.2	
251.4	200.5	0.043	0.3	1.254	T04G9.3	similar to mammalian lectin VIP36
1517.5	1210.7	0.043	0.3	1.253	C26F1.4	ribosomal protein/ubiquitin-like protein
94.1	75.1	0.042	0.3	1.253	F53A10.2	
933.6	745.2	0.045	0.3	1.253	g533166	
267.3	213.4	0.047	0.3	1.253	C11D2.4	
1133.5	905	0.042	0.3	1.252	F28C6.7A	ribosomal protein L26 like
2009.4	1604.4	0.042	0.3	1.252	F36A2.6	40S ribosomal protein S15
54.1	43.2	0.047	0.3	1.252	C54E10.6	

719	574.2	0.044	0.3	1.252	F27D4.1	electron transfer flavoprotein alpha-subunit
2126.7	1698.6	0.043	0.3	1.252	AV191236	
70.6	56.4	0.05	0.3	1.252	F20D1.6	Human mRNA KIAA0066 predicted protein like
345.7	276.3	0.044	0.3	1.251	Y66H1B.4	
110.6	88.4	0.05	0.3	1.251	AU116387_rc	
265.1	211.9	0.045	0.3	1.251	T04B8.5	
1969.1	1574.4	0.043	0.3	1.251	T04C12.6	actin
96.3	77	0.045	0.3	1.251	Y53F4B.6	
1453.9	1162.9	0.048	0.3	1.250	Y75B12B.2	Peptidyl-prolyl cis-trans isomerases
988.8	791	0.048	0.3	1.250	C46F11.2	pyridine nucleotide-disulphide oxidoreductases class-I
59.5	47.6	0.041	0.3	1.250	K10H10.6	Alcohol other dehydrogenases, short chain type
2026.9	1621.8	0.043	0.3	1.250	ZK652.4	60S ribosomal protein L35
984.4	788.3	0.043	0.3	1.249	F36H1.1	FK506-binding protein
1009.4	808.4	0.045	0.3	1.249	K01G5.4	GTP-binding protein
298.3	239	0.05	0.3	1.248	ZK353.6	Leucine aminopeptidase
529	423.9	0.049	0.3	1.248	F28H1.3	aminoacyl-tRNA synthetase
192.8	154.6	0.046	0.3	1.247	Y113G7B.16	
2515	2018.3	0.045	0.3	1.246	F10E7.7	60s ribosomal protein L35A
1479.1	1187.3	0.049	0.3	1.246	C56C10.8	Transcription factor BTF3 (human)
346.5	278.2	0.044	0.3	1.246	T20B12.7	
51.3	41.2	0.05	0.3	1.245	F43H9.1	enoyl-coA hydratase
1683.3	1352.2	0.046	0.3	1.245	R11A5.4	phosphoenolpyruvate carboxykinase
1681.6	1352.1	0.048	0.3	1.244	F37C12.9	Ribosomal protein S14
949	763.1	0.046	0.3	1.244	Y38F2AR.D_rc	
543.9	437.4	0.046	0.3	1.243	K12H4.4	Signal peptidase
1871.5	1505.1	0.042	0.3	1.243	F37C12.11	
600.8	484.6	0.048	0.3	1.240	F20G2.2	dehydrogenase
725.6	585.4	0.05	0.3	1.239	C39F7.4	RAS-related protein
68.4	55.2	0.05	0.3	1.239	T06E6.3	7 transmembrane receptor (rhodopsin family)
2000.8	1616.1	0.046	0.3	1.238	C53H9.1	60S ribosomal protein L27
2112.9	1707.3	0.049	0.3	1.238	AV200380_rc	
1698.4	1373.8	0.05	0.3	1.236	CEK100E6F	
145.3	179.5	0.048	-0.3	0.809	H12I13.4	RNA-binding protein
112.9	139.9	0.049	-0.3	0.807	R08D7.2	
104.8	130.2	0.049	-0.3	0.805	CEK053F1R_rc	
65.5	81.4	0.043	-0.3	0.805	K01A11.2	Yeast YIL-0 like protein
68.3	84.9	0.046	-0.3	0.804	F36H2.2	
133.3	165.7	0.05	-0.3	0.804	C36B1.3	DNA directed RNA polymerase II

76.5	95.1	0.05	-0.3	0.804	F52C12.2A	
41.9	52.1	0.048	-0.3	0.804	AV201725	
92.2	114.8	0.046	-0.3	0.803	CEK119E6R_rc	
127.6	158.9	0.045	-0.3	0.803	ZK686.4	
163.9	204.2	0.048	-0.3	0.803	C59705_rc	
66.8	83.3	0.049	-0.3	0.802	Y106G6H.16	
98.3	122.6	0.044	-0.3	0.802	D1007.7	RNA-binding protein
71.1	88.7	0.048	-0.3	0.802	EEED8.9	
141.7	177	0.047	-0.3	0.801	F42H10.2	
69.8	87.2	0.05	-0.3	0.800	F22B5.1	GTP-binding ADP-ribosylation factor
360.6	450.7	0.05	-0.3	0.800	T26A5.3	NADH-ubiquinone oxidoreductase
71.2	89	0.043	-0.3	0.800	F57B9.4	
143.9	179.9	0.044	-0.3	0.800	g5731102	
89.1	111.4	0.044	-0.3	0.800	C41G7.1	Human muscular atrophy protein like
92.2	115.3	0.044	-0.3	0.800	C32F10.2	
120.5	150.7	0.046	-0.3	0.800	T20H4.4	
99	124	0.042	-0.3	0.798	C02C2.6	
248.7	311.6	0.045	-0.3	0.798	CEK006BXR_rc	
58	72.7	0.05	-0.3	0.798	C27A12.9	
155.1	194.5	0.043	-0.3	0.797	W04D2.4	Zinc finger, C2H2 type
71.2	89.3	0.041	-0.3	0.797	C16C10.6	
71.9	90.2	0.045	-0.3	0.797	C56476_rc	
137.4	172.4	0.041	-0.3	0.797	CEK020F3R_rc	
110.7	138.9	0.04	-0.3	0.797	Y47D3A.26	
102.8	129	0.044	-0.3	0.797	F52B5.5	cep-1/p53
37.2	46.7	0.047	-0.3	0.797	F08H9.1	
145.2	182.5	0.042	-0.3	0.796	CEK086E5R_rc	
61.5	77.3	0.046	-0.3	0.796	T07F10.3	polyadenylate-binding protein like
76.5	96.2	0.037	-0.3	0.795	C35D10.7A	
70.2	88.4	0.043	-0.3	0.794	Y71F9B.6	
39.2	49.4	0.038	-0.3	0.794	Y50D4A.J	
77.5	97.7	0.042	-0.3	0.793	F08B4.5	
78.6	99.1	0.045	-0.3	0.793	F25B4.4	
112.5	141.9	0.039	-0.3	0.793	AV184696_rc	
56.1	70.8	0.05	-0.3	0.792	R74.6	DOM34 protein
119.4	150.7	0.048	-0.3	0.792	T03G11.8	human leukocyte surface protein (weak)
54.5	68.8	0.037	-0.3	0.792	AV198775_rc	
65.4	82.6	0.034	-0.3	0.792	T21C9.1	discs large like repeats

174.8	221.1	0.034	-0.3	0.791	CEK044A9R_rc	
47.5	60.1	0.041	-0.3	0.790	F43E2.3	
64.8	82	0.036	-0.3	0.790	C29H12.1	arginyl-tRNA synthetase
58.7	74.3	0.038	-0.3	0.790	C27A12.2	zinc finger protein
31.2	39.5	0.048	-0.3	0.790	C30B5.4	Probable RNA binding protein
152.4	193	0.032	-0.3	0.790	CEK008FZR_rc	
78.9	100	0.035	-0.3	0.789	F59A6.6	ribonuclease H
45.6	57.8	0.039	-0.3	0.789	Y43B11AR.C	
102.2	129.6	0.045	-0.3	0.789	C54160_rc	
196.5	249.3	0.033	-0.3	0.788	CEK006CZR_rc	
153.3	194.5	0.039	-0.3	0.788	ZK970.3	
78.8	100.1	0.034	-0.4	0.787	F21H12.5	
225.2	286.2	0.036	-0.4	0.787	C34561_rc	
74.1	94.2	0.034	-0.4	0.787	T02E1.2	
44.4	56.5	0.032	-0.4	0.786	Y75B8A.16	
77.7	98.9	0.033	-0.4	0.786	R02D3.7	
162.3	206.6	0.032	-0.4	0.786	Y23H5A.3	
64.4	82	0.035	-0.4	0.785	R10D12.13B	
67.3	85.7	0.035	-0.4	0.785	CEK019A4R_rc	
186.6	238	0.033	-0.4	0.784	F23H11.1	bra-2 - (BMP Receptor Associated protein family)
107.6	137.3	0.045	-0.4	0.784	D1014.8	
44.8	57.2	0.037	-0.4	0.783	ZK593.8	
132.8	169.6	0.041	-0.4	0.783	K01G5.2C	hpl-2 - (HP1 Like (heterochromatin protein))
70.7	90.3	0.033	-0.4	0.783	Y65B4A.J	
362.8	463.9	0.048	-0.4	0.782	g6635138	
112.6	144	0.041	-0.4	0.782	CEK131FXR_rc	
37.6	48.1	0.032	-0.4	0.782	ZK1058.5	
158.7	203.1	0.032	-0.4	0.781	T09E8.2	him-17 - (High Incidence of Males (increased X chromosome loss))
39.2	50.2	0.029	-0.4	0.781	C03D6.3	mRNA capping enzyme
62.9	80.6	0.032	-0.4	0.780	F33G12.3	
131.6	168.7	0.03	-0.4	0.780	AU115194_rc	
200.6	257.2	0.027	-0.4	0.780	F58A4.2	
96.9	124.3	0.041	-0.4	0.780	AU115882_rc	
49.1	63	0.028	-0.4	0.779	Y105E8B.X	
148.9	191.1	0.04	-0.4	0.779	C39E9.13	Human activator 1 38 KD subunit like
43.3	55.6	0.029	-0.4	0.779	C06A5.3	
52.8	67.8	0.028	-0.4	0.779	F37D6.1	mus-101 - (MUS (Drosophila mutagen sensitive) related), BRCA1 C Terminus (BR
197.6	253.9	0.028	-0.4	0.778	K07A1.1	

51.9	66.7	0.039	-0.4	0.778	AU116236_rc	
58.9	75.7	0.025	-0.4	0.778	ZK632.11	
81.9	105.3	0.026	-0.4	0.778	K03B8.4	
127.9	164.6	0.026	-0.4	0.777	CEK078D4R_rc	
63	81.1	0.046	-0.4	0.777	C18E3.2	
41.3	53.2	0.042	-0.4	0.776	AU111466	
119.3	153.7	0.028	-0.4	0.776	C33201_rc	
101.4	130.7	0.026	-0.4	0.776	T27E9.5	phosphatidylserine synthase I
35.8	46.2	0.042	-0.4	0.775	CE7801	
63.4	81.9	0.024	-0.4	0.774	Y10G11A.B	
372.5	481.2	0.032	-0.4	0.774	D2030.6	
178.4	230.5	0.024	-0.4	0.774	F20A1.9	
224.2	289.8	0.042	-0.4	0.774	F20D12.1	
131.4	169.9	0.05	-0.4	0.773	CEK044D5R_rc	
110.9	143.4	0.025	-0.4	0.773	ZK1067.3	
35.1	45.4	0.024	-0.4	0.773	C45G9.2	
38.8	50.2	0.03	-0.4	0.773	K02B2.4	
101.2	131	0.026	-0.4	0.773	F44E7.5	
133.7	173.1	0.024	-0.4	0.772	C48E7.2	
77.9	101	0.023	-0.4	0.771	CEK011E7R_rc	
171.4	222.4	0.031	-0.4	0.771	T28D9.4	
94.1	122.2	0.029	-0.4	0.770	CEC280_rc	
106.1	137.8	0.046	-0.4	0.770	ZC477.5	
32.8	42.6	0.045	-0.4	0.770	K09C4.8	sulfatase
93.7	121.7	0.03	-0.4	0.770	F18A1.8	
100.5	130.6	0.024	-0.4	0.770	D1054.14	
117.9	153.3	0.024	-0.4	0.769	AU116717_rc	
188.8	245.5	0.022	-0.4	0.769	g2677835	
67.1	87.3	0.031	-0.4	0.769	C09H6.3	
27.9	36.3	0.035	-0.4	0.769	Y71F9AR.A	
72	93.7	0.023	-0.4	0.768	Y41D4A.D	
461.2	600.4	0.022	-0.4	0.768	R13H4.2	eggshell protein like
133.7	174.1	0.025	-0.4	0.768	K01G5.2B	hpl-2 - (HP1 Like (heterochromatin protein))
24.4	31.8	0.048	-0.4	0.767	T28A8.5	
125.6	163.9	0.021	-0.4	0.766	K03H1.2	RNA helicase (DEAH subfamily)
100.2	130.8	0.02	-0.4	0.766	F43G9.10	mfap-1 - (MicroFibrillar-Associated Protein homolog)
61.8	80.7	0.023	-0.4	0.766	JC8.7	
117.4	153.4	0.022	-0.4	0.765	T09A5.8	chromosomal organisation modifier protein

79.2	103.5	0.023	-0.4	0.765	T20D4.11	
131.4	171.9	0.02	-0.4	0.764	F10B5.2	
85	111.2	0.021	-0.4	0.764	F58A4.6	
164.1	214.7	0.021	-0.4	0.764	CEK105DYR_rc	
303.5	397.4	0.035	-0.4	0.764	M60.3	
38.9	51	0.021	-0.4	0.763	W03G11.3	alpha-L-fucosidase precursor
53.2	69.8	0.024	-0.4	0.762	C39D10.3	B. subtilis glycerol-3-phosphate cytidylyltransferase
47.4	62.2	0.018	-0.4	0.762	D1007.8	
90.9	119.3	0.034	-0.4	0.762	R07B7.2	
78.4	102.9	0.042	-0.4	0.762	F02E9.6	WD domain, G-beta repeat
128	168.1	0.033	-0.4	0.761	T04A8.10	non-histone chromosomal protein HMG-1
72.4	95.1	0.023	-0.4	0.761	Y75B8A.25	
100.3	131.8	0.018	-0.4	0.761	C09G9.2	
134	176.1	0.018	-0.4	0.761	F59C6.4	
85.3	112.1	0.02	-0.4	0.761	K10D2.5	
33.1	43.5	0.019	-0.4	0.761	C13G3.2	
87.8	115.4	0.019	-0.4	0.761	g1006800	
49.6	65.2	0.02	-0.4	0.761	C32D5.10	
57.7	75.9	0.042	-0.4	0.760	ZC376.6	
29.8	39.2	0.023	-0.4	0.760	Y54H5A.4	
78.6	103.4	0.018	-0.4	0.760	AV185445_rc	
114.4	150.5	0.036	-0.4	0.760	C53D6.4	angiotensin receptor like
158.9	209.1	0.027	-0.4	0.760	F42A9.6	
29.1	38.3	0.024	-0.4	0.760	F46F11.8	
36	47.4	0.015	-0.4	0.759	Y59A8B.11	
24	31.6	0.046	-0.4	0.759	T05A8.7	
28.1	37	0.025	-0.4	0.759	K08F9.4	
50.3	66.3	0.032	-0.4	0.759	Y39G10AL.A	
37.4	49.3	0.017	-0.4	0.759	T09F3.4	
124.5	164.2	0.021	-0.4	0.758	F38H4.10	Yeast hypothetical protein YIK6 like
32.6	43	0.032	-0.4	0.758	C44F1.2	
128.8	169.9	0.019	-0.4	0.758	AU116146_rc	
135.4	178.8	0.036	-0.4	0.757	CE1241	
26.3	34.8	0.047	-0.4	0.756	F23C8.9	
24.1	31.9	0.033	-0.4	0.755	H34C03.1	
145.8	193.1	0.035	-0.4	0.755	F54C9.9	
85.6	113.4	0.031	-0.4	0.755	K01G5.1	Zinc finger, C3HC4 type (RING finger)
74.4	98.6	0.042	-0.4	0.755	R06C7.9	Zinc finger, C2H2 type (5 domains)

56.4	74.8	0.041	-0.4	0.754	ZK1307.9	YJU2 protein
24.2	32.1	0.042	-0.4	0.754	F39B2.7	
87.3	115.8	0.016	-0.4	0.754	F45E4.9	
69.1	91.7	0.016	-0.4	0.754	C14B1.6	
58.7	77.9	0.026	-0.4	0.754	Y45F10D.9	
84	111.5	0.017	-0.4	0.753	C56A3.4	Zinc finger, C3HC4 type (RING finger)
151.1	200.7	0.017	-0.4	0.753	T23G7.5	tyrosine specific protein phosphatase
36.2	48.1	0.047	-0.4	0.753	F39H2.1	
99.7	132.5	0.016	-0.4	0.752	B0207.6	
158.4	210.6	0.016	-0.4	0.752	C46A5.6	
116.1	154.5	0.016	-0.4	0.751	CEK031D1R_rc	
192.2	255.8	0.05	-0.4	0.751	C36A4.5	claustrin like
80.3	106.9	0.037	-0.4	0.751	C06A5.9	rnf-1 - (RiNg Finger protein)
121.2	161.5	0.016	-0.4	0.750	F10B5.6	cell division control protein
137.1	182.7	0.031	-0.4	0.750	R53.6	
57.9	77.2	0.049	-0.4	0.750	C02F5.3	GTP-binding protein
104.9	139.9	0.032	-0.4	0.750	CEK011H2R_rc	
210.3	280.6	0.029	-0.4	0.749	C34B2.7	sdha-1- mitochondrial flavoprotein
130.9	174.7	0.044	-0.4	0.749	C48B6.2	ribosomal protein
86	114.8	0.019	-0.4	0.749	C02F5.1	
39.6	52.9	0.014	-0.4	0.749	F23H11.2	
75.3	100.6	0.017	-0.4	0.749	R08D7.1	
21.7	29	0.035	-0.4	0.748	F52C6.10	
49.3	65.9	0.035	-0.4	0.748	CEK045A2R_rc	
78.1	104.4	0.015	-0.4	0.748	T07E3.5	
51.5	68.9	0.027	-0.4	0.747	F28C1.1	
164.9	220.7	0.04	-0.4	0.747	C33037_rc	
55.6	74.5	0.036	-0.4	0.746	F25B3.6	
60.6	81.2	0.015	-0.4	0.746	B0252.4	Cyclophilin
157.9	211.6	0.039	-0.4	0.746	C09H10.6	histone binding protein
101.1	135.5	0.038	-0.4	0.746	F29F11.3	
26.3	35.3	0.031	-0.4	0.745	Y38F2AR.N	
33.6	45.1	0.026	-0.4	0.745	ZK287.6	Zinc finger, C2H2 type (3 domains)
80	107.4	0.014	-0.4	0.745	F56D1.1	Zinc finger protein
75.1	100.9	0.012	-0.4	0.744	C33F10.4	
69.4	93.3	0.025	-0.4	0.744	F36H2.2	
245.1	329.6	0.035	-0.4	0.744	W05H9.1	
173.2	233	0.012	-0.4	0.743	T24D1.2	

179.6	241.7	0.013	-0.4	0.743	C56447_rc	
88.2	118.7	0.045	-0.4	0.743	C63302	
28	37.7	0.041	-0.4	0.743	T28A8.7	DNA mismatch repair protein
106.7	143.7	0.031	-0.4	0.743	Y39A1A.12	ATPases associated with various cellular activities (AAA)
54.2	73	0.014	-0.4	0.742	F25H5.5	
38	51.2	0.033	-0.4	0.742	R06C7.2	
101.3	136.5	0.013	-0.4	0.742	C26B2.1	
62.4	84.1	0.012	-0.4	0.742	Y52D3.1	serine/threonine kinase
59.9	80.8	0.048	-0.4	0.741	C33016_rc	
33.4	45.1	0.04	-0.4	0.741	C33H5.6	
53.2	72	0.012	-0.4	0.739	F33G12.2	
76.9	104.1	0.034	-0.4	0.739	CEK087F5R_rc	
46.9	63.5	0.018	-0.4	0.739	T04H1.5	
34.1	46.2	0.036	-0.4	0.738	CEK111B7R_rc	
240.2	325.5	0.012	-0.4	0.738	H14N18.3	
186.4	252.7	0.039	-0.4	0.738	T21B10.4	
112.1	152	0.049	-0.4	0.738	CEK003H4R_rc	
82.8	112.3	0.032	-0.4	0.737	Y39G10AR.3	nekl-1 Serine/threonine protein kinase
111.4	151.1	0.011	-0.4	0.737	C40H1.5	Transthyretin-like family
156.8	212.7	0.013	-0.4	0.737	C54848_rc	
102.3	139	0.011	-0.4	0.736	F25B5.5	
36.5	49.6	0.017	-0.4	0.736	Y39B6B.R	
158.8	215.8	0.03	-0.4	0.736	B0432.2	
83.7	113.8	0.01	-0.4	0.736	VC5.4	
63.6	86.5	0.037	-0.5	0.735	W10D5.3C	
62.4	84.9	0.011	-0.5	0.735	Y113G7B.5	
38.5	52.4	0.029	-0.5	0.735	M02B7.1	
43.7	59.5	0.039	-0.5	0.734	C39B5.6	amidotransferase
43.3	59	0.011	-0.5	0.734	C43E11.2	
128.3	174.9	0.011	-0.5	0.734	CEK011DZR_rc	
23.3	31.8	0.02	-0.5	0.733	AU116611_rc	
72.9	99.5	0.037	-0.5	0.733	g2911175	
80.4	109.8	0.011	-0.5	0.732	K06H7.5	
123.5	168.8	0.036	-0.5	0.732	CEK034G7R_rc	
136.8	187	0.009	-0.5	0.732	ZK856.9	
152.5	208.5	0.015	-0.5	0.731	T04A8.8	
50.6	69.2	0.024	-0.5	0.731	g9965902	
52.7	72.1	0.009	-0.5	0.731	E02H1.2	GTP binding protein

130.3	178.3	0.021	-0.5	0.731	C14A4.5	Prokaryotic ribonuclease PH
60.2	82.4	0.02	-0.5	0.731	T23B12.1	
21.6	29.6	0.022	-0.5	0.730	Y43F11A.6	
131.1	179.7	0.027	-0.5	0.730	M7.2	kinesin
54.7	75	0.013	-0.5	0.729	Y106G6H.6	
158.6	217.5	0.01	-0.5	0.729	CEC280_rc	
189.4	259.8	0.014	-0.5	0.729	g706923	
97.1	133.3	0.009	-0.5	0.728	T12A2.7	
82.6	113.4	0.027	-0.5	0.728	C34021_rc	
65.4	89.8	0.026	-0.5	0.728	Y48G1BL.H	
72.6	99.7	0.02	-0.5	0.728	C27H6.3	
102.5	140.8	0.009	-0.5	0.728	D2096.7	
120.9	166.1	0.012	-0.5	0.728	AV180510_rc	
115.5	158.7	0.03	-0.5	0.728	CEK003E3R_rc	
52.1	71.6	0.01	-0.5	0.728	F10G8.7	
132.5	182.1	0.016	-0.5	0.728	CEK009H2R_rc	
73.6	101.2	0.017	-0.5	0.727	W05F2.7	
26.1	35.9	0.022	-0.5	0.727	F22D3.5	
147.9	203.5	0.033	-0.5	0.727	C35D10.13	
147.4	202.9	0.008	-0.5	0.726	CEK127C9R_rc	
715.4	985.4	0.024	-0.5	0.726	C15C8.3	cathepsin-like protease
47.6	65.6	0.021	-0.5	0.726	C30732_rc	
137.4	189.4	0.017	-0.5	0.725	Y14H12B.1	zinc-finger protein
47.8	65.9	0.014	-0.5	0.725	F55G1.5	carrier protein
88.3	121.9	0.031	-0.5	0.724	AV179650_rc	
300.9	415.4	0.023	-0.5	0.724	ZK381.4	
126	174	0.008	-0.5	0.724	CEK002G4R_rc	
80.5	111.2	0.028	-0.5	0.724	C49C3.7	
48.5	67	0.009	-0.5	0.724	F57C2.1	
221.6	306.3	0.009	-0.5	0.723	g5762324	
64.8	89.6	0.046	-0.5	0.723	C13G5.2	
60.8	84.1	0.019	-0.5	0.723	W07E11.2	FMRFamide neuropeptide repeats
43.3	59.9	0.011	-0.5	0.723	Y95B8A.11	
122.2	169.2	0.008	-0.5	0.722	T01E8.4	WD domain, G-beta repeats
34.2	47.4	0.004	-0.5	0.722	ZK177.5	cytochrome p450
97.6	135.3	0.009	-0.5	0.721	B0547.1	
88.2	122.3	0.031	-0.5	0.721	B0511.7	
56.1	77.8	0.008	-0.5	0.721	F57B10.6	DNA repair protein

141.6	196.4	0.008	-0.5	0.721	g7417256	
99.2	137.6	0.008	-0.5	0.721	W05B10.2	
32.5	45.1	0.027	-0.5	0.721	F31D5.2	
33.4	46.4	0.005	-0.5	0.720	Y48G1C.E	
44.6	62	0.018	-0.5	0.719	Y54G11A.1	
88.4	122.9	0.042	-0.5	0.719	AV178862_rc	
30.7	42.7	0.03	-0.5	0.719	F22D6.8	
132.5	184.4	0.008	-0.5	0.719	g2795932	
76.3	106.2	0.008	-0.5	0.718	F44E2.7	
22.7	31.6	0.014	-0.5	0.718	T20D4.9	
59.2	82.5	0.008	-0.5	0.718	Y39A3CR.1	
38	53	0.007	-0.5	0.717	Y57A10A.8	
49.4	68.9	0.015	-0.5	0.717	CEC748_rc	
151.5	211.7	0.012	-0.5	0.716	CEK005GXR_rc	
54.4	76.1	0.006	-0.5	0.715	C04E12.2	
81.4	113.9	0.024	-0.5	0.715	B0025.2	
31.3	43.8	0.004	-0.5	0.715	CD4.8	
137.5	192.6	0.021	-0.5	0.714	F18A1.5	replication factor A
527	738.3	0.007	-0.5	0.714	F19B6.1A	uridine kinase
74.8	104.8	0.006	-0.5	0.714	F37A4.9	
55.6	77.9	0.007	-0.5	0.714	M18.3	
228.1	319.6	0.02	-0.5	0.714	CEK038E4R_rc	
32.4	45.4	0.022	-0.5	0.714	Y53C10A.7	
82.9	116.2	0.023	-0.5	0.713	F58G11.5	
98.3	137.8	0.018	-0.5	0.713	F55A12.1	
30.6	42.9	0.022	-0.5	0.713	R144.9	
76.1	106.7	0.019	-0.5	0.713	g473870	
46.7	65.5	0.033	-0.5	0.713	AV181657_rc	
38	53.3	0.006	-0.5	0.713	F25H2.12	
78.2	109.7	0.007	-0.5	0.713	C41G7.4	set-32 - (SET (trithorax/polycomb) domain containing), Histone H3 (Lys9) methyltr
41.9	58.8	0.006	-0.5	0.713	E02H1.3	Yeast DEG-1 protein homolog
65.2	91.5	0.034	-0.5	0.713	F35G12.1	
19.3	27.1	0.02	-0.5	0.712	Y102A5C.18	
98.4	138.2	0.028	-0.5	0.712	B0464.6	
110.4	155.2	0.006	-0.5	0.711	T20B12.2	
60.2	84.7	0.025	-0.5	0.711	C18E3.8	7 trans-membrane protein
22.6	31.8	0.011	-0.5	0.711	F01D4.5	
47.9	67.4	0.031	-0.5	0.711	Y17G9B.3	cytochrome P450

79	111.4	0.036	-0.5	0.709	CEK005DXR_rc	
516.3	728.2	0.015	-0.5	0.709	F35G2.4	prolyl 4-hydroxylase alpha subunit
139.1	196.2	0.006	-0.5	0.709	F42G10.1	
58.9	83.1	0.006	-0.5	0.709	Y113G7B.7	
81	114.4	0.016	-0.5	0.708	F47B8.2	
66.3	93.7	0.006	-0.5	0.708	F56D2.2	
34	48.1	0.005	-0.5	0.707	C04F5.9	
57.8	81.8	0.006	-0.5	0.707	F13G3.6	B.subtilis teichoic acids biosynthesis protein GGAB like- (glycosyltransferase)
47.9	67.8	0.009	-0.5	0.706	R07E5.11	
118.3	167.5	0.008	-0.5	0.706	g4558870	
139.3	197.3	0.043	-0.5	0.706	C51624_rc	
81.8	115.9	0.048	-0.5	0.706	T19B10.7	nucleoprotein interactor 1 like
117.3	166.4	0.005	-0.5	0.705	T02G5.12	
64.9	92.1	0.008	-0.5	0.705	Y49A3A.3	
66.2	94	0.043	-0.5	0.704	C32071_rc	
48.7	69.2	0.005	-0.5	0.704	F32B6.3	Human HPRP18 protein like
94	133.6	0.018	-0.5	0.704	Y110A7A.15	
30.8	43.8	0.003	-0.5	0.703	R07E5.1	
140.7	200.3	0.027	-0.5	0.702	C38491_rc	
49.1	69.9	0.017	-0.5	0.702	D1054.13	
56.4	80.3	0.018	-0.5	0.702	F11A10.3	Zinc finger, C3HC4 type (RING finger)
36.1	51.4	0.017	-0.5	0.702	Y32B12B.4	
220.6	314.1	0.021	-0.5	0.702	T01C3.2	
103.9	148	0.022	-0.5	0.702	F25H8.1	Yeast protein 00926 like
78.5	111.9	0.015	-0.5	0.702	C06G4.1	possible RNA binding protein
18.8	26.8	0.016	-0.5	0.701	F43H9.3	
23	32.8	0.008	-0.5	0.701	Y50D4A.D	
54.2	77.3	0.038	-0.5	0.701	Y48G1C.G	
38.2	54.5	0.03	-0.5	0.701	C32090_rc	
60.2	85.9	0.01	-0.5	0.701	AU115890_rc	
68.7	98.1	0.033	-0.5	0.700	Y50E8A.4	
41.8	59.7	0.025	-0.5	0.700	Y47G6A.25	
42.2	60.3	0.005	-0.5	0.700	T12F5.1	
180.4	257.8	0.007	-0.5	0.700	EEED8.1	
450.4	643.8	0.014	-0.5	0.700	AV188776	
75.1	107.4	0.005	-0.5	0.699	T05H4.14	
20.2	28.9	0.041	-0.5	0.699	Y46E12BL.E	
37.3	53.4	0.035	-0.5	0.699	Y45G5AM.I	

75.3	107.9	0.005	-0.5	0.698	R11A8.2	
39.3	56.4	0.004	-0.5	0.697	C10B5.1	
120.1	172.5	0.046	-0.5	0.696	CEK014B2R_rc	
46.2	66.4	0.025	-0.5	0.696	T04A8.15	glucose-6-phosphate 1-dehydrogenase
73	105	0.005	-0.5	0.695	C33596_rc	
60.9	87.6	0.035	-0.5	0.695	g8886067	
41.6	59.9	0.05	-0.5	0.694	C54D2.2	
83.1	119.7	0.005	-0.5	0.694	W02A2.6	rec-8 meiosis-specific cohesin complex subunit
20.4	29.4	0.008	-0.5	0.694	F19F10.11A	
21.3	30.7	0.023	-0.5	0.694	K12H6.2	
17.9	25.8	0.015	-0.5	0.694	Y37H9A.1	
43	62	0.004	-0.5	0.694	F21H12.1	
85.8	124.2	0.004	-0.5	0.691	Y71F9AL.8	
53.9	78.1	0.01	-0.5	0.690	F57C2.2	btb-19 - (BTB (Broad/complex/Tramtrack/Bric a brac) domain protein)
87.7	127.1	0.006	-0.5	0.690	C52802_rc	
85.9	124.5	0.004	-0.5	0.690	g10242309	
20.9	30.3	0.008	-0.5	0.690	D2096.10	
70.2	101.8	0.007	-0.5	0.690	C24B5.2	
177.3	257.2	0.005	-0.5	0.689	AV177145_rc	
42.1	61.1	0.004	-0.5	0.689	K02B12.5	ankyrin motif
13.5	19.6	0.037	-0.5	0.689	W04D2.3	Ogre family
17.8	25.9	0.013	-0.5	0.687	Y57A10A.5	
65.4	95.2	0.004	-0.5	0.687	Y97E10AR.3	
54.3	79.1	0.008	-0.5	0.686	F43D9.5	
144	209.8	0.004	-0.5	0.686	CEK041E5R_rc	
54.9	80	0.003	-0.5	0.686	Y73B6A.4	
47.9	69.8	0.05	-0.5	0.686	K04F10.6	
95.1	138.6	0.008	-0.5	0.686	CEK002D8R_rc	
38.6	56.3	0.012	-0.6	0.686	Y75B8A.22	
25.7	37.5	0.01	-0.6	0.685	AU114925_rc	
101.7	148.5	0.007	-0.6	0.685	CEK114H7R_rc	
86	125.6	0.004	-0.6	0.685	B0336.7	
96.2	140.5	0.003	-0.6	0.685	C53344_rc	
43.4	63.4	0.012	-0.6	0.685	C63639	
163.7	239.2	0.011	-0.6	0.684	C51641_rc	
38.1	55.7	0.01	-0.6	0.684	Y110A7A.1	
108.4	158.5	0.031	-0.6	0.684	CEK037G6R_rc	
45.5	66.6	0.037	-0.6	0.683	C58475_rc	

20.7	30.3	0.014	-0.6	0.683	C16A3.2	protein-tyrosine phosphatase
112.5	164.7	0.017	-0.6	0.683	B0361.9	
54.5	79.8	0.006	-0.6	0.683	C30435_rc	
107.7	157.7	0.006	-0.6	0.683	Y69A2AR.C	
28.4	41.6	0.002	-0.6	0.683	R10E4.5	endonuclease
60.2	88.2	0.006	-0.6	0.683	T05E7.3	
133.5	195.6	0.006	-0.6	0.683	T03F1.9	
137.6	201.7	0.026	-0.6	0.682	C56157_rc	
67.1	98.4	0.005	-0.6	0.682	R11A8.2	
69.2	101.7	0.018	-0.6	0.680	CEK129C2R_rc	
38.5	56.6	0.048	-0.6	0.680	Y53C10A.6	
18.7	27.5	0.008	-0.6	0.680	F10G8.9	
31.4	46.2	0.008	-0.6	0.680	C36A4.8	
57.9	85.2	0.02	-0.6	0.680	C58619_rc	
68.6	101.1	0.003	-0.6	0.679	AU114749_rc	
25.1	37	0.002	-0.6	0.678	C50F4.4	
46.4	68.4	0.038	-0.6	0.678	ZC410.3	Man(9)-alpha-mannosidase, N-linked glycosylation site
75.9	111.9	0.01	-0.6	0.678	T19B10.3	beta-galactosidase
79	116.5	0.014	-0.6	0.678	CEK016E7R_rc	
127	187.6	0.002	-0.6	0.677	C32E8.5	
82.1	121.3	0.006	-0.6	0.677	CEK027D1R_rc	
33.9	50.1	0.032	-0.6	0.677	AU114019_rc	
29.5	43.6	0.001	-0.6	0.677	T28A8.3	
70.7	104.5	0.048	-0.6	0.677	C35D10.6	Aldose reductase
37.4	55.3	0.029	-0.6	0.676	DY3.4	Reverse transcriptase (RNA-dependent DNA polymerase)
178	263.5	0.01	-0.6	0.676	C54668_rc	
92.8	137.4	0.003	-0.6	0.675	g2795932	
68.8	101.9	0.008	-0.6	0.675	CEK113B7R_rc	
86.5	128.2	0.015	-0.6	0.675	C32F10.5	single-strand recognition protein
42.3	62.7	0.042	-0.6	0.675	D2085.6	phosphatidylinositol biosynthetic protein
72.3	107.2	0.007	-0.6	0.674	F42A9.8	
39.3	58.3	0.01	-0.6	0.674	Y47G6A.14	zinc finger protein
135.2	200.6	0.022	-0.6	0.674	F42A6.3	
40.1	59.6	0.002	-0.6	0.673	CEC8693_rc	
11.5	17.1	0.05	-0.6	0.673	R11D1.1B	WD domain, G-beta repeat
15.8	23.5	0.027	-0.6	0.672	Y105E8B.G	
27.7	41.2	0.004	-0.6	0.672	Y60A3A.8	
69.3	103.1	0.034	-0.6	0.672	B0001.2	

111.6	166.1	0.004	-0.6	0.672	g6478469	cki-2 - (CKI family (Cyclin-dependent Kinase Inhibitor))
52.7	78.5	0.038	-0.6	0.671	W02D9.4	
24.3	36.2	0.003	-0.6	0.671	Y32B12B.2	
102.5	152.7	0.034	-0.6	0.671	T23E7.2D	
64.9	96.8	0.046	-0.6	0.670	F26A1.1	
30.2	45.1	0.042	-0.6	0.670	Y54G9A.5	
84	125.5	0.006	-0.6	0.669	C53851_rc	
28.7	42.9	0.006	-0.6	0.669	C33F10.2	Cell division control protein
40.8	61	0.01	-0.6	0.669	R04B5.3	nhr-205 - (Nuclear Hormone Receptor family)
13.7	20.5	0.023	-0.6	0.668	C17H12.6	
33.8	50.6	0.018	-0.6	0.668	C53A5.2	Neurospora met-10 protein like
68.3	102.3	0.024	-0.6	0.668	CEK097E8R_rc	
28.1	42.1	0.002	-0.6	0.667	Y105E8B.T	
64.5	96.7	0.01	-0.6	0.667	AV178599_rc	
28.2	42.3	0.004	-0.6	0.667	AU114949_rc	
55.3	83	0.002	-0.6	0.666	F59E12.2	
93.2	139.9	0.006	-0.6	0.666	F08A8.7	
48.6	73	0.003	-0.6	0.666	Y43F11A.2	
126.6	190.2	0.009	-0.6	0.666	g440174	
92.4	138.9	0.016	-0.6	0.665	F07A5.1B	inx-14 - (INneXin)
14.5	21.8	0.016	-0.6	0.665	C06H2.3	Acetyltransferase (GNAT) family
40.1	60.3	0.029	-0.6	0.665	g7542552	
115.6	173.9	0.002	-0.6	0.665	C06A8.4	
32.3	48.6	0.009	-0.6	0.665	CD4.7	
95.5	143.7	0.011	-0.6	0.665	D2030.7	
148.7	224.3	0.004	-0.6	0.663	CEK010B3R_rc	
18.8	28.4	0.004	-0.6	0.662	K09E10.1	
60.2	91	0.024	-0.6	0.662	AU116131_rc	
29.2	44.2	0.001	-0.6	0.661	F26H9.7	ubiquitin-conjugating enzyme
102	154.4	0.01	-0.6	0.661	R06C7.8	Eukaryotic protein kinase domain
62.9	95.3	0.018	-0.6	0.660	g5031477	
13.2	20	0.031	-0.6	0.660	C49H3.4	
35.9	54.4	0.041	-0.6	0.660	g2343264	
103.2	156.5	0.004	-0.6	0.659	F02A9.6	glp-1 - (abnormal Germ Line Proliferation),LIN-12/Notch family receptor
43.4	65.9	0.015	-0.6	0.659	C56E6.3	
136.6	207.5	0.007	-0.6	0.658	C52573_rc	
14.8	22.5	0.013	-0.6	0.658	F59A1.9	
63.4	96.6	0.007	-0.6	0.656	AV184943_rc	

121.7	185.7	0.01	-0.6	0.655	C38669_rc	
74.3	113.4	0.007	-0.6	0.655	C56324_rc	
28.5	43.5	0.025	-0.6	0.655	K10B4.6	
33.5	51.2	0.003	-0.6	0.654	R12C12.8	
73.8	112.8	0.002	-0.6	0.654	AV183883_rc	
47.1	72	0.004	-0.6	0.654	AV181329_rc	
38.1	58.3	0.001	-0.6	0.654	M04B2.2	
19	29.1	0.003	-0.6	0.653	Y39B6A.H	
48.3	74	0.038	-0.6	0.653	T04H1.2	GTP-binding protein
63.1	96.7	0.002	-0.6	0.653	T01B11.3	syntaxin
55.1	84.5	0.03	-0.6	0.652	AV182621_rc	
69.5	106.9	0.001	-0.6	0.650	F12F6.7	DNA polymerase delta, regulatory subunit 55
25.8	39.7	0.034	-0.6	0.650	Y52B11A.10	
18.3	28.2	0.007	-0.6	0.649	F41E7.2	
412.4	635.6	0.001	-0.6	0.649	F19B6.2A	
52.4	80.8	0.018	-0.6	0.649	CE440	
97.9	151	0.02	-0.6	0.648	M03E7.5	
114.4	176.9	0.003	-0.6	0.647	AU114875_rc	
108	167.1	0.014	-0.6	0.646	C48B4.7	
24.8	38.4	0.006	-0.6	0.646	C29F9.12	
35.2	54.6	0.041	-0.6	0.645	C55A6.3	dehydrogenase
84.6	131.3	0.021	-0.6	0.644	C52262_rc	
65.3	101.4	0.008	-0.6	0.644	AU114499_rc	
96	149.1	0.015	-0.6	0.644	C32152_rc	
55.3	86.1	0.011	-0.6	0.642	T10E9.1	
67.5	105.3	0.003	-0.6	0.641	Y37D8A.11	
14.1	22	0.009	-0.6	0.641	C17F4.6	guanylate cyclase
28.6	44.7	0	-0.7	0.640	C33C12.9	
90.9	142.2	0.033	-0.6	0.639	CEC7193_rc	
49.5	77.5	0.002	-0.7	0.639	AU115852_rc	
22.8	35.7	0.001	-0.7	0.639	Y55D9A.2A	
47.8	75	0.032	-0.7	0.637	ZC328.4	
53.2	83.6	0.001	-0.7	0.636	C34824_rc	
188.8	296.9	0.05	-0.7	0.636	CEK033G2R_rc	
115.4	181.7	0.002	-0.7	0.635	Y51A2D.10	Transthyretin-like family
20	31.5	0.004	-0.7	0.635	M01E11.3	
57	89.9	0.002	-0.7	0.634	Y56A3A.4	
38.8	61.3	0.05	-0.7	0.633	AV182333_rc	

44.1	69.7	0.001	-0.7	0.633	F59H6.11	
24.6	38.9	0.037	-0.7	0.632	AU114474_rc	
31.9	50.5	0.039	-0.7	0.632	ZK328.4	
43.4	68.8	0.01	-0.7	0.631	ZK675.2	DNA repair protein (REV1)
27.3	43.3	0	-0.7	0.630	C46A5.5	
24.3	38.6	0.001	-0.7	0.630	D1007.10	
18.8	29.9	0.003	-0.7	0.629	M04F3.2	
60.9	97	0.002	-0.7	0.628	H26D21.1	
38.6	61.5	0.019	-0.7	0.628	AV180884_rc	
54.9	87.6	0.032	-0.7	0.627	F55A11.8	
37.2	59.4	0.002	-0.7	0.626	F42F12.2	zig-2 - (2 (Zwei) IG domain protein)
41.5	66.4	0.022	-0.7	0.625	CEK081D3R_rc	
70.1	112.2	0.004	-0.7	0.625	C57167_rc	
35.1	56.3	0.004	-0.7	0.623	C02F5.6	
23.8	38.2	0.014	-0.7	0.623	Y59A8A.2	
11.4	18.3	0.033	-0.7	0.623	R06A10.1	
46.9	75.5	0.025	-0.7	0.621	F08F1.9	
75.9	122.5	0.004	-0.7	0.620	F10B5.5	ATP binding protein
9.4	15.2	0.034	-0.7	0.618	T05B4.2	nhr-57 - (Nuclear Hormone Receptor family)
39.5	64	0.009	-0.7	0.617	M01F1.8	
94.6	153.4	0.012	-0.7	0.617	C05D2.5	
30.4	49.3	0.01	-0.7	0.617	T27D1.1	cyn-9 - (CYclophyliN)
29.9	48.6	0.017	-0.7	0.615	C35E7.8	
16.1	26.2	0.002	-0.7	0.615	F07F6.4	
69.6	113.5	0.012	-0.7	0.613	CEK088D6R_rc	
118.4	193.2	0.01	-0.7	0.613	H21P03.2	
61.9	101.2	0.001	-0.7	0.612	C17G10.4B	
16.5	27	0.014	-0.7	0.611	T02G5.6	
29.1	47.7	0.002	-0.7	0.610	Y57A10A.25	
60.9	99.9	0.036	-0.7	0.610	C50C3.1	
46.2	75.8	0.043	-0.7	0.609	F10E7.11	
14.5	23.8	0.005	-0.7	0.609	ZK1128.2	
33.1	54.4	0.014	-0.7	0.608	Y56A3A.17A	
61.8	101.8	0.039	-0.7	0.607	W06A7.4	
16.5	27.2	0.046	-0.7	0.607	AV175982_rc	
40.5	66.8	0.001	-0.7	0.606	Y39A1A.13	
22.4	37	0.024	-0.7	0.605	K10C8.1	
12.2	20.2	0.012	-0.7	0.604	C47E8.8	SET domain

9.3	15.4	0.044	-0.7	0.604	C53424_rc	
43.5	72.1	0.002	-0.7	0.603	T26A5.6	
29.5	49	0.028	-0.7	0.602	g6010656	
44.7	74.5	0.039	-0.7	0.600	T02E1.3B	gla-3 - (Germ Line Apoptosis abnormal), contains two CCCH-like zinc-finger doma
87.4	146	0.001	-0.7	0.599	C03B8.2	
127.4	212.9	0.002	-0.7	0.598	C38D4.4	
14.5	24.3	0.032	-0.8	0.597	C34D1.4	
9.9	16.6	0.016	-0.8	0.596	K08E4.2	
18.3	30.7	0.029	-0.8	0.596	B0205.1	
35.4	59.4	0	-0.8	0.596	AU114268_rc	
33	55.4	0.015	-0.8	0.596	F31C3.5	
24	40.3	0	-0.8	0.596	Y71F9AL.10	
7.8	13.1	0.05	-0.8	0.595	C18H9.7	postsynaptic protein
11.9	20	0.009	-0.7	0.595	H43I07.1	
17	28.8	0.01	-0.8	0.590	R02D1.1	
50.6	86.5	0.008	-0.8	0.585	AU113843_rc	
76.2	130.3	0.043	-0.8	0.585	CEK025B1R_rc	
26	44.5	0.029	-0.8	0.584	K11B4.2	
15.1	25.9	0.038	-0.8	0.583	R11D1.10	
39	66.9	0.001	-0.8	0.583	F20B10.1	neurexin III like
25.3	43.4	0	-0.8	0.583	T10E9.2	
15.6	26.8	0.001	-0.8	0.582	Y54E10BR.A	
7.8	13.4	0.036	-0.8	0.582	F59H6.10	
14.9	25.6	0.014	-0.8	0.582	K06A9.3	
11.4	19.6	0.02	-0.8	0.582	Y39B6A.E	
15.4	26.5	0.001	-0.8	0.581	F59H6.9	
33.4	57.6	0.001	-0.8	0.580	R08D7.5	Caltractin
46.2	80.2	0.049	-0.8	0.576	F58G1.2	Zinc finger, C2H2 type (6 domains)
11	19.1	0.015	-0.8	0.576	Y53C10A.13	
32.7	57	0.001	-0.8	0.574	F52C6.11	
71.8	125.2	0.006	-0.8	0.573	C42C1.7	
19.1	33.4	0.001	-0.8	0.572	T06C12.4	
188.4	329.6	0.001	-0.8	0.572	F48D6.4	
259.1	455.1	0	-0.8	0.569	CEK016A9R_rc	
32.7	57.5	0.002	-0.8	0.569	C29H12.5	
30.5	53.8	0.01	-0.8	0.567	Y54E10A.12	
21.6	38.2	0.017	-0.8	0.565	C55494_rc	
21.5	38.1	0.022	-0.8	0.564	W06D11.4	

29.3	52.2	0.02	-0.8	0.561	AU113125_rc	
46.9	83.8	0.009	-0.8	0.560	Y54E5B.3	
8.6	15.4	0.036	-0.8	0.558	H12D21.4	Rhodanese-like domain
15.4	27.6	0.026	-0.8	0.558	B0336.1	
27	48.6	0.002	-0.9	0.556	R08H2.1	dehydrogenase
30	54	0.006	-0.9	0.556	g459698	
30.1	54.6	0.039	-0.9	0.551	F43G6.3	predicted pseudogene
201.5	366.8	0	-0.9	0.549	g3372863	
18.1	33	0.016	-0.9	0.548	C34C12.2	
59.3	108.3	0.004	-0.9	0.548	F09G2.2	
251.6	463	0	-0.9	0.543	C42C1.5	guanyltransferase
11.5	21.2	0.005	-0.9	0.542	Y116A8C.3	
12.8	23.6	0.033	-0.9	0.542	F48C1.8	
12.6	23.3	0.038	-0.9	0.541	W03A3.2	DNA polymerase
29.5	54.8	0.001	-0.9	0.538	C16C8.5	
22.3	41.7	0.01	-0.9	0.535	C33H5.3	
14.2	26.6	0.001	-0.9	0.534	R06F6.4	
11.9	22.3	0.002	-0.9	0.534	K01A2.6	
304.7	574.3	0	-0.9	0.531	F35G2.2	E.coli YCAC like
11.1	21	0.013	-0.9	0.529	W04B5.5	protein kinase
31.1	58.9	0.033	-0.9	0.528	ZK550.4	
10.8	20.5	0.001	-0.9	0.527	C55187_rc	
28.1	53.5	0.001	-0.9	0.525	C49C3.6	
252	479.9	0	-0.9	0.525	C42C1.12	
11.6	22.2	0.017	-0.9	0.523	3R5.1	
24	46.1	0	-0.9	0.521	Y110A2AL.13	
15.7	30.3	0.038	-1	0.518	F22B7.9	
38.2	74.3	0	-1	0.514	AV180585_rc	
32.7	63.7	0.006	-1	0.513	AU116571_rc	
6.2	12.1	0.039	-1	0.512	Y52E8A.1	
23.2	45.3	0.001	-1	0.512	Y111B2A.1	
26.6	52	0.01	-1	0.512	F22E5.9	
37.9	74.3	0.001	-1	0.510	CEK116F3R_rc	
42.3	83.1	0	-1	0.509	F58G11.3	
19	37.4	0.034	-1	0.508	C44B9.3	
5.5	10.9	0.04	-1	0.505	Y53C12A.2	excitatory amino acid transporter
255.6	508.2	0	-1	0.503	C42C1.11	peptidase
45.8	91.1	0.002	-1	0.503	CEK005AYR_rc	

6.3	12.6	0.05	-1	0.500	F14F8.8	
10.4	20.9	0	-1	0.498	F59H6.12	
8.7	17.6	0.014	-1	0.494	CEK131G9R_rc	
8.5	17.2	0.017	-1	0.494	Y110A2AL.9	
8.3	16.8	0.019	-1	0.494	R11E3.3	reverse transcriptase
9.1	18.5	0.023	-1	0.492	F40F9.10	
8	16.3	0.004	-1	0.491	M01F1.1	alpha-1,3-mannosyl-glycoprotein beta-1,2-N-acetylglucosaminyltransferase
25.7	52.7	0.01	-1	0.488	F14D2.1	
17.7	36.3	0	-1	0.488	C36C5.12	
12.6	25.9	0.046	-1	0.486	ZC84.1	Serine protease inhibitor, Kunitz type
13.7	28.2	0.011	-1	0.486	F19F10.10	
17	35.1	0.001	-1	0.484	F58E6.7	
93.2	192.7	0.001	-1.1	0.484	C42C1.4	
25.8	54.6	0	-1.1	0.473	T06D10.2	
34	72	0.046	-1.1	0.472	Y40B1B.6	
34.8	73.9	0.001	-1.1	0.471	B0238.1	carboxylesterase
9.4	20	0.026	-1.1	0.470	T10C6.8	Uncharacterized protein
9.1	19.7	0.004	-1.1	0.462	F41G4.5	Uncharacterized protein
13	28.2	0.002	-1.1	0.461	ZK1127.11	him-14 - (High Incidence of Males (increased X chromosome loss))
13.5	29.3	0.049	-1.1	0.461	T22G5.6	lbp-8 - (Lipid Binding Protein)
9.9	21.5	0	-1.1	0.460	F36H12.2	Uncharacterized protein
13.1	28.6	0.007	-1.1	0.458	F22B7.6	polk-1 - (POLK (DNA polymerase kappa) homolog)
17.9	39.1	0.005	-1.1	0.458	C33H5.15	sgo-1 - ((S. pombe chromosome segregation protein) homolog)
22.2	48.5	0.01	-1.1	0.458	T23D8.7	Uncharacterized protein
43.6	95.3	0.004	-1.1	0.458	F32H5.3	Uncharacterized protein
56.6	124.3	0	-1.1	0.455	F16A11.3	ppfr-1 - (Protein Phosphatase Four Regulatory subunit)
174	384.5	0	-1.1	0.453	C42C1.10	Mitochondrial solute carrier protein
41.6	92.1	0.002	-1.2	0.452	ZC302.1	mre-11 - (yeast MRE recombination/repair homolog)
28.9	65.3	0	-1.2	0.443	Y39C12A.2	predicted pseudogene
70.7	159.8	0	-1.2	0.442	C42C1.13	Uncharacterized protein
463.9	1050.8	0	-1.2	0.441	F21F8.4	Aspartyl protease
30.6	69.7	0.001	-1.2	0.439	Y113G7B.4	ftr-1 - (Fog Two (fog-2) Related), F-box protein
7.8	18	0.027	-1.2	0.433	R05C11.1	Uncharacterized protein
36.2	83.7	0.024	-1.2	0.432	Y73C8C.2	clec-210 - (C-type LECtin)
26.2	60.6	0.001	-1.2	0.432	T04D3.8	Uncharacterized protein
22.2	51.7	0.043	-1.2	0.429	K08E7.3	let-99 - (LEThal), a protein containing a DEP domain (Domain found in Dishevelle
16.9	39.4	0.042	-1.2	0.429	Y97E10AR.4	Uncharacterized protein
16.5	38.5	0	-1.2	0.429	B0238.13	Carboxylesterase and related protein

16	37.6	0.005	-1.2	0.426	Y47H9C.8	Small secreted protein with conserved cysteines
13.7	32.2	0.04	-1.2	0.425	T26A5.2	Uncharacterized protein
9.1	21.6	0.042	-1.3	0.421	F52C6.8	bath-4 - (BTB and MATH domain containing)
22.6	53.8	0.018	-1.3	0.420	Y108G3AL.2	Putative RNA binding protein
14.1	33.9	0.001	-1.3	0.416	K09B11.2	nol-9 - (NucleOLar protein), similar to ATP/GTP-binding protein
12.3	29.8	0	-1.3	0.413	F57C9.7	Uncharacterized protein
52.5	128	0.004	-1.3	0.410	K07C6.3	cyp-35B2 - (CYtochrome P450 family)
20.1	49.8	0.048	-1.3	0.404	R08C7.10	wapl-1 - (WAPL (Drosophila Wings APart-Like cohesin interactor))
5.6	13.9	0.002	-1.3	0.403	T19C4.6	gpa-1 - (G Protein, Alpha subunit)
82.4	204.6	0.007	-1.3	0.403	Y105E8B.3	riok-2 - (RIO Kinase homolog)
7.9	19.7	0.021	-1.3	0.401	T07C4.10	Uncharacterized protein
48.4	120.7	0.001	-1.3	0.401	F59A7.2	Uncharacterized protein
74.2	185.5	0	-1.3	0.400	C42C1.8	Permease of the major facilitator superfamily
23.6	59	0.011	-1.3	0.400	C08B11.6	Actin-related protein - Arp6p
5.3	13.3	0.042	-1.3	0.398	Y53H1A.2	Uncharacterized protein
11.7	29.5	0	-1.3	0.397	C18A3.1	Transcriptional activator, adenine-specific DNA methyltransferase
12.3	31.2	0.001	-1.3	0.394	ZK402.5	Uncharacterized protein
71.6	183.6	0	-1.4	0.390	Y39C12A.1	Uncharacterized protein
5.1	13.1	0.025	-1.4	0.389	C10F3.1	cpg-4 - (Chondroitin ProteoGlycan)
32.7	86.2	0	-1.4	0.379	Y39C12A.9	Uncharacterized protein
109.9	290.8	0	-1.4	0.378	F44A2.1	tag-153 - predicted transcriptional regulator
9.2	24.5	0.047	-1.4	0.376	C02C6.1	dyn-1 - (DYNamin related)
6.2	16.7	0.016	-1.4	0.371	ZK673.3	THAP-like domain protein
8.3	22.5	0	-1.4	0.369	T20D4.8	neprilysin, a thermolysin-like zinc metallopeptidase
20	54.3	0	-1.4	0.368	F20B10.1	nlr-1 - (Neurexin Like receptor)
20.8	57.2	0.001	-1.5	0.364	Y46C8AL.D	clec-70 - (C-type LECtin)
13.5	37.2	0	-1.5	0.363	C34E10.8	Uncharacterized protein
19.6	54.6	0.003	-1.5	0.359	Y47G6A.1	inx-21 - (INneXin), an integral transmembrane channel protein
10.4	29.7	0.023	-1.5	0.350	Y52E8A.2	Predicted E3 ubiquitin ligase
3.1	9.1	0.047	-1.5	0.341	Y62H9A.2	Uncharacterized protein
63.7	187.4	0	-1.6	0.340	R02E12.6	hrg-1 - (Heme Responsive Gene)
211.1	622.3	0.027	-1.6	0.339	K02B2.1	Fructose-6-phosphate 2-kinase/fructose-2,6-biphosphatase
16.4	48.6	0.027	-1.6	0.337	T06A10.4	Chromatin remodeling protein, contains PHD Zn-finger
2.8	8.3	0.031	-1.6	0.337	Y38C1AB.2	Uncharacterized protein
15.1	45.5	0	-1.6	0.332	F28D1.6	Uncharacterized protein
20.7	63.2	0	-1.6	0.328	Y39C12A.8	dnj-26 - (DNaJ domain (prokaryotic heat shock protein))
3.8	11.7	0.012	-1.6	0.325	Y53G8AR.2	PHD finger protein
30.9	96.8	0	-1.7	0.319	Y69H2.9	Uncharacterized protein

6.8	21.4	0.004	-1.7	0.318	ZC443.5	ugt-18 - (UDP-GlucuronosylTransferase)
16.5	52	0.026	-1.7	0.317	H12I13.4	fbf-1 - (Fem-3 mRNA Binding Factor), translational repressor Pumilio/PUF3 and re
14.3	46.1	0.001	-1.7	0.310	T24D1.5	Uncharacterized protein
2.7	8.8	0.047	-1.7	0.307	C23H4.2	Carboxylesterase and related protein
6.1	20.1	0.049	-1.7	0.303	H01G02.1	Uncharacterized protein
4	14	0.036	-1.8	0.286	Y71H2AM.7	Uncharacterized protein
20.3	71.9	0.014	-1.8	0.282	C25D7.6	mcm-3 - (yeast MCM (licensing factor) related)
6.2	22.3	0.007	-1.8	0.278	Y46H3A.4	Predicted lipase
4	14.6	0.001	-1.9	0.274	C52A10.2	Carboxylesterase and related protein
4.9	18.1	0.002	-1.9	0.271	C34D4.10	Uncharacterized protein
12.7	47	0.001	-1.9	0.270	F55B12.4	tRNA nucleotidyltransferase/poly(A) polymerase
56.9	213.9	0	-1.9	0.266	F44A2.1	tag-153 - predicted transcriptional regulator
6.4	24.3	0.005	-1.9	0.263	C14C6.3	Predicted glycosyltransferase
3.3	12.9	0.023	-2	0.256	F54G2.2	Uncharacterized protein
24.1	95	0.017	-2	0.254	F01G10.3	ech-9 - (Enoyl-CoA Hydratase)
3.4	13.5	0.034	-2	0.252	Y70D2A.2	hex-5 - (HEXosaminidase)
5	21	0	-2.1	0.238	ZK616.2	Uncharacterized protein
3.2	13.7	0.002	-2.1	0.234	K09E10.2	oac-58 - (O-ACyltransferase homolog)
2.4	10.3	0.02	-2.1	0.233	F59H6.1	bath-19 - (BTB and MATH domain containing)
6	25.9	0.002	-2.1	0.232	C38D4.9	Predicted RNA methylase
9.6	42.2	0	-2.1	0.227	T28A8.4	Uncharacterized protein
5.2	23.8	0.004	-2.2	0.218	F20B10.2	predicted pseudogene
3.9	22.6	0	-2.5	0.173	C08B11.2	hda-2 - (Histone DeAcetylase)
8.6	50.6	0.047	-2.6	0.170	C33H5.19	tag-321 - uncharacterized protein
16	99.9	0.005	-2.6	0.160	T10H10.2	FAD-dependent sulfhydryl oxidase/quiescin and related protein
2.8	17.5	0.011	-2.7	0.160	B0403.3	Uncharacterized protein
1.4	8.8	0.006	-2.7	0.159	C49C8.3	Uncharacterized protein
7.3	46	0.001	-2.7	0.159	Y37H2A.1	TatD-related DNase
2.8	21.3	0.007	-3	0.131	M01E11.1	Farnesyl cysteine-carboxyl methyltransferase
2.1	16.1	0.002	-2.9	0.130	Y18H1A.6	pif-1 - (PIF1p DNA helicase (yeast) homolog)
18.6	143.7	0	-3	0.129	F44A2.1	tag-153 - predicted transcriptional regulator
1.7	14.9	0.004	-3.2	0.114	T13G4.1	GYF domain containing protein
2.1	18.6	0.015	-3.2	0.113	ZK1236.1	Elongation factor-type GTP-binding protein
4	38.8	0	-3.3	0.103	F26A3.7	Uncharacterized protein
22.8	225.5	0.002	-3.3	0.101	T25C12.2	spp-9 - (SaPosin-like Protein family)
1.6	16.1	0	-3.3	0.099	Y17G9B.4	Cyclophilin type peptidyl-prolyl cis-trans isomerase
2	21.1	0.02	-3.4	0.095	Y74C9A.3	Hydroxyindole-O-methyltransferase and related SAM-dependent methyltransferase
1.5	18.8	0	-3.6	0.080	C33C12.8	Uncharacterized protein

0.7	9.2	0.015	-3.8	0.076	C47B2.7B	selb-1 - (SELB (SelB homolog) translation factor for selenocysteine incorporation)
2.5	38.2	0	-3.9	0.065	F46G11.3	tag-257 - ARK protein kinase family
0.5	7.8	0.004	-4	0.064	T22H2.1	sri-12 - (Serpentine Receptor, class I)
1.5	27.2	0.017	-4.2	0.055	K04C2.4	brd-1 - (BaRD homolog (tumor suppressor gene Bard1))
2	39	0.003	-4.3	0.051	F58A4.4	pri-1 - (DNA PRImase homolog)
1.6	40	0.004	-4.6	0.040	F01D4.8	Cystathionine beta-synthase and related enzyme
0.6	26.3	0.007	-5.6	0.023	F56F11.4	26S proteasome regulatory complex, ATPase RPT6
0.2	10.3	0.002	-5.5	0.019	C34F11.1	Uncharacterized protein
0.6	31.2	0.002	-5.7	0.019	ZK507.4	dos-1 - (Delta and OSM-11-like)
0.3	22.2	0	-6	0.014	B0304.2	Uncharacterized protein
