# Supplementary material for: Regulation of genes affecting body size and innate immunity by the DBL-1/BMP-like pathway in Caenorhabditis elegans
Source: BMC Dev Biol. 2010 Jun 7;10:61. doi: 10.1186/1471-213X-10-61 (PMC2894779; doi:10.1186/1471-213X-10-61)
Supplement: Additional file 3 — Protein synthesis and degradation genes highly regulated at 95% confidence or above. A summary list of protein synthesis and degradation genes regulated by the Sma/Mab pathway at the 95% confidence level. [file 1471-213X-10-61-S3.PDF]

Factor	P-value	Gene	Function
Protein degradation			
0.4	0.033	K09A9.4	ubiquitin C-terminal hydrolase
0.4	0.031	Y39A1C.2	HECT-domain ubiquitin transferase
0.6	0.005	K08E7.7	LIN-19-like protein, *cul-6*-cullin family
0.4	0.031	C26F1.4	ubiquitin-like protein
0.3	0.043	C26F1.4	ubiquitin-like protein
0.4	0.017	H06I04.4	ubiquitin
1	0.032	F45H11.2	ubiquitin
0.4	0.047	F07A11.4	ubiquitin carboxyl-terminal hydrolase
0.3	0.037	K08C9.7	ubiquitin family
0.4	0.03	F34H10.1	ubiquitin/ribosomal protein
0.4	0.023	C47E12.5	ubiquitin-activating enzyme
-0.6	0.001	F26H9.7	ubiquitin-conjugating enzyme
0.4	0.026	M7.1	ubiquitin-conjugating enzyme E2-17 KD
0.4	0.014	ZK1010.1	UBQ-2 ubiquitin; 60S ribosomal protein L40
0.9	0.032	E03H4.8	beta' coatomer protein like
0.6	0.019	T13F2.8	caveolin
0.4	0.017	C56A3.7	caveolin
0.4	0.043	K11D2.3	clathrin coat assembly protein
-3.6	0	C33C12.8	glucosylceramidase
1.1	0.001	ZC190.1	*cln-3.3*/lysosomal protein
1	0.017	F19C7.2	lysosomal carboxypeptidase
1.1	0.001	F19C7.4	lysosomal carboxypeptidase
1	0.001	F23B2.11	lysosomal carboxypeptidase
0.7	0.001	C26B9.5	lysosomal Pro-X carboxypeptidase
0.8	0.007	F23H12.1	synaptobrevin like
Protein synthesis			
0.4	0.032	B0393.1	40S ribosomal protein
0.4	0.028	F53A3.3	40S ribosomal protein
0.4	0.016	D1007.6	40S ribosomal protein S10
0.3	0.042	F36A2.6	40S ribosomal protein S15
0.4	0.016	T01C3.6	40S ribosomal protein S16
0.4	0.03	T08B2.10	40S ribosomal protein S17
0.5	0.012	T08B2.10	40S ribosomal protein S17
0.4	0.015	F39B2.6	40S ribosomal protein S26
0.4	0.019	T05E11.1	40S ribosomal protein S5
0.4	0.03	Y71A12B.1	40S ribosomal protein S6
0.4	0.029	ZC434.2	40S ribosomal protein S7
0.4	0.031	F42C5.8	40S ribosomal protein S8
0.4	0.032	C09H10.2	60S ribosomal protein
0.3	0.039	F52B5.6	60S ribosomal protein

0.4	0.028	K11H12.2	60S ribosomal protein L15
0.4	0.015	C09D4.5	60S ribosomal protein L19
0.4	0.02	D1007.12	60S ribosomal protein L24
0.3	0.046	C53H9.1	60S ribosomal protein L27
0.4	0.018	B0513.3	60S ribosomal protein L29
0.4	0.013	F13B10.2	60S ribosomal protein L3
0.3	0.043	ZK652.4	60S ribosomal protein L35
0.3	0.045	F10E7.7	60s ribosomal protein L35A
0.4	0.017	C54C6.1	60S ribosomal protein L37
0.5	0.011	F54C9.5	60S ribosomal protein L5
0.6	0.038	F20D1.5	ADP-ribosylation factor
0.7	0.03	F45E4.1	ADP-ribosylation factor
0.4	0.018	F07D10.1	ribosomal protein
0.4	0.028	E04A4.8	ribosomal protein
0.3	0.039	K02B2.5	ribosomal protein
-0.4	0.044	C48B6.2	ribosomal protein
0.4	0.023	F53G12.10	ribosomal protein
0.4	0.03	F10B5.1	ribosomal protein L10 (QM protein)
0.4	0.034	F10B5.1	ribosomal protein L10 (QM protein)
0.5	0.011	C32E8.2	ribosomal protein L13
0.4	0.028	C14B9.7	ribosomal protein L21
0.3	0.034	C27A2.2	ribosomal protein L22
0.4	0.025	F55D10.2	ribosomal protein L23
0.3	0.042	F28C6.7A	ribosomal protein L26 like
0.4	0.027	W09C5.6B	ribosomal protein L31e
0.3	0.034	C26F1.9	ribosomal protein L39
0.4	0.027	R13A5.8	ribosomal protein L9
0.4	0.027	F40F11.1	ribosomal protein S11
0.5	0.023	Y57G11C.16	ribosomal protein S13
0.3	0.048	F37C12.9	ribosomal protein S14
0.4	0.024	F37C12.9	ribosomal protein S14
0.4	0.032	T05F1.3	ribosomal protein S19e
0.3	0.034	F28D1.7	ribosomal protein S23
0.4	0.016	F28D1.7	ribosomal protein S23
0.4	0.025	C23G10.3	ribosomal protein S3
0.4	0.027	C23G10.3	ribosomal protein S3
0.5	0.009	F56F3.5	ribosomal protein S3a (human) homolog
0.3	0.041	F40F8.10	ribosomal protein S9
0.4	0.016	B0250.1	ribosomal protein L2
0.4	0.028	R11D1.8	ribosomal protein L28 like
