# Supplementary material for: Regulation of genes affecting body size and innate immunity by the DBL-1/BMP-like pathway in Caenorhabditis elegans
Source: BMC Dev Biol. 2010 Jun 7;10:61. doi: 10.1186/1471-213X-10-61 (PMC2894779; doi:10.1186/1471-213X-10-61)
Supplement: Additional file 4 — Structural Genes highly regulated at 95% confidence or above. A summary list of structural genes regulated by the Sma/Mab pathway at the 95% confidence level. [file 1471-213X-10-61-S4.PDF]

Factor	P-value	Gene	Function
0.4	0.025	M03F4.2	actin
0.4	0.03	T04C12.5	actin
0.3	0.043	T04C12.6	actin
0.4	0.032	C38C3.5A	actin depolymerizing factor
0.5	0.005	T25C8.2	Actins
0.5	0.028	K02A11.1	ankyrin motifs, Myosin phosphatase
0.5	0.009	B0336.2	*arf-1.2*/ ADP-ribosylation factor
0.3	0.041	C36E8.5	beta tubulin
1.1	0.035	F15A4.8	chitinase
1	0.017	R09D1.11	chitinase
0.5	0.005	F32A7.5	claustrin-like microtubule-associated protein
0.5	0.039	C30F2.1	collagen
0.5	0.026	T07H6.3	collagen
0.4	0.013	T10E10.1	collagen
0.4	0.014	T10E10.2	collagen
0.5	0.03	D2023.7	collagen
0.4	0.022	F15H10.1	collagen
0.4	0.029	F15H10.2	collagen
0.4	0.025	F55C10.2	collagen
0.4	0.033	F57B1.4	collagen
0.5	0.014	C29F4.1	collagen
0.5	0.047	C53B4.5	collagen
0.4	0.05	W08D2.6	collagen
0.5	0.038	F38A3.2	collagen
0.4	0.03	W09G10.1	collagen
0.7	0.016	T11F9.9	*col-157*/collagen
0.5	0.012	T28C6.6	cuticle collagen
0.5	0.005	C29E4.1	Cuticle collagen
0.4	0.02	F52B11.4	*col-34*/collagen
0.5	0.033	F41F3.3	cuticlin
0.6	0.045	F53F1.5	cuticlin
0.4	0.041	F36A4.10	cuticular collagen
0.5	0.034	K02D7.3	cuticular collagen
0.9	0.015	K01A2.7_rc	cuticular collagen
0.5	0.014	F53B6.6	cuticulin
0.4	0.041	F11G11.10	*col-17*/collagen
0.4	0.03	T14B4.6	*dpy-2*/collagen
0.5	0.022	F30B5.1	*dpy-13*/collagen
0.4	0.035	Y43C5A.2	Fibrinogen beta and gamma chains, C-terminal globular domain

1.2	0.018	F37B4.2	intermediate filament protein
0.6	0.023	F10C1.7B	intermediate filament protein
-0.5	0.027	M7.2	kinesin
0.5	0.004	T02C12.1	myosin IA
1.8	0.01	K04C1.4	myosin light chain
0.5	0.027	F54F3.1	*nid-1*/nidogen like
1.3	0.018	T28D6.2	tubulin alpha subunit
0.6	0.003	F44F4.11	tubulin alpha-2 chain
