# Supplementary material for: Regulation of genes affecting body size and innate immunity by the DBL-1/BMP-like pathway in Caenorhabditis elegans
Source: BMC Dev Biol. 2010 Jun 7;10:61. doi: 10.1186/1471-213X-10-61 (PMC2894779; doi:10.1186/1471-213X-10-61)
Supplement: Additional file 5 — Comparisons with similar microarray experiments. A summary list of comparisons among similar microarray experiments. Mochii et al refers to reference [36], Liang et al refers to reference [37], and Mallo et al refers to reference [5]. [file 1471-213X-10-61-S5.PDF]

Gene	Factor	P-value	up-regulated in Mochii et al	up-regulated in Liang et al	up-regulated in Mallo et al
K01A2.3	1.7	0.014	Yes	Yes	
K01A2.3	1.7	0.006			
F35C5.6/clec-63	0.7	0.001	Yes		
F35C5.8/clec-65	1.2	0	Yes	Yes	
F35C5.9/clec-66	2.6	0	Yes		
K10C2.1/ lysosomal cathepsin A	0.5	0.004	Yes		
R09H10.5	0.7	0.001	Yes		
C08E3.13	1.2	0.013	Yes	Yes	
F55G11.4/DUF141	2.5	0			Yes
W04E12.8/lectin domain	0.5	0.006			Yes
ZK6.7/lipase	0.9	0.002			Yes
Lysozyme genes					
lys-1	0.7	0.001			Yes
lys-2	1.2	0			
lys-7	0.8	0.022			Yes
lys-8	0.4	0.017	Yes		Yes
lys-9	0.7	0.032			
